# Burden of Neurological Disorders Across the US From 1990-2017: A Global Burden of Disease Study

**DOI:** 10.1001/jamaneurol.2020.4152

**Published:** 2020-11-02

**Authors:** GBD 2017 US Neurological Disorders Collaborators, Valery L. Feigin, Theo Vos, Fares Alahdab, Arianna Maever L. Amit, Till Winfried Bärnighausen, Ettore Beghi, Mahya Beheshti, Prachi P. Chavan, Michael H. Criqui, Rupak Desai, Samath Dhamminda Dharmaratne, E. Ray Dorsey, Arielle Wilder Eagan, Islam Y. Elgendy, Irina Filip, Simona Giampaoli, Giorgia Giussani, Nima Hafezi-Nejad, Michael K. Hole, Takayoshi Ikeda, Catherine Owens Johnson, Rizwan Kalani, Khaled Khatab, Jagdish Khubchandani, Daniel Kim, Walter J. Koroshetz, Vijay Krishnamoorthy, Rita V. Krishnamurthi, Xuefeng Liu, Warren David Lo, Giancarlo Logroscino, George A. Mensah, Ted R. Miller, Salahuddin Mohammed, Ali H. Mokdad, Maziar Moradi-Lakeh, Shane Douglas Morrison, Veeresh Kumar N. Shivamurthy, Mohsen Naghavi, Emma Nichols, Bo Norrving, Christopher M. Odell, Elisabetta Pupillo, Amir Radfar, Gregory A. Roth, Azadeh Shafieesabet, Aziz Sheikh, Sara Sheikhbahaei, Jae Il Shin, Jasvinder A. Singh, Timothy J. Steiner, Lars Jacob Stovner, Mitchell Taylor Wallin, Jordan Weiss, Chenkai Wu, Joseph Raymond Zunt, Jaimie D. Adelson, Christopher J. L. Murray

**Affiliations:** 1 Faculty of Health and Environmental Sciences, Auckland University of Technology School of Public Health and Psychosocial Studies, Auckland, New Zealand; 2 Institute for Health Metrics and Evaluation, University of Washington, Seattle; 3 Research Center of Neurology, Moscow, Russia; 4 Department of Health Metrics Sciences, University of Washington School of Medicine, Seattle; 5 Mayo Evidence-Based Practice Center, Mayo Clinic Foundation for Medical Education and Research, Rochester, Minnesota; 6 Department of Epidemiology and Biostatistics, University of the Philippines Manila, Manila, Philippines; 7 Johns Hopkins University School of Public Health, Baltimore, Maryland; 8 Heidelberg Institute of Global Health, Heidelberg University, Heidelberg, Germany; 9 Harvard University T.H. Chan School of Public Health, Boston, Massachusetts; 10 Department of Neuroscience, Mario Negri Institute for Pharmacological Research, Milan, Italy; 11 Department of Physical Medicine and Rehabilitation, New York University, New York; 12 Department of Epidemiology and Environmental Health, the University of Buffalo, Buffalo, New York; 13 Department of Family Medicine and Public Health, University of California, San Diego, La Jolla; 14 Division of Cardiology, Atlanta Veterans Affairs Medical Center, Decatur, Georgia; 15 Department of Community Medicine, University of Peradeniya, Peradeniya, Sri Lanka; 16 University of Rochester, Rochester, New York; 17 Department of Global Health and Social Medicine, Harvard University, Boston, Massachusetts; 18 Department of Social Services, Tufts Medical Center, Boston, Massachusetts; 19 Division of Cardiology, Massachusetts General Hospital, Boston; 20 Division of Cardiology, Harvard University, Boston, Massachusetts; 21 Psychiatry Department, Kaiser Permanente, Fontana, California; 22 A.T. Still University School of Osteopathic Medicine in Arizona, Arizona School of Health Sciences, Mesa, Arizona; 23 Department of Cardiovascular Endocrine-Metabolic Diseases and Aging, Istituto Superiore di Sanità (Italian National Institute of Health), Rome, Italy; 24 Laboratory of Neurological Disorders, Mario Negri Institute for Pharmacological Research, Milan, Italy; 25 Department of Radiology and Radiological Science, Johns Hopkins University, Baltimore, Maryland; 26 Tehran University of Medical Sciences School of Medicine, Tehran, Iran; 27 Department of Pediatrics, The University of Texas, Austin, Austin; 28 Department of Biostatistics and Epidemiology, Auckland University of Technology, Auckland, New Zealand; 29 Department of Neurology, University of Washington, Seattle; 30 Faculty of Health and Wellbeing, Sheffield Hallam University, Sheffield, United Kingdom; 31 Ohio University College of Arts and Sciences, Zanesville; 32 Department of Nutrition and Health Science, Ball State University, Muncie, Indiana; 33 Department of Health Sciences, Northeastern University, Boston, Massachusetts; 34 National Institutes of Neurological Disorders and Stroke, National Institute of Health, Bethesda, Maryland; 35 Department of Anesthesiology, Duke University, Durham, North Carolina; 36 Department of Anesthesiology, University of Washington, Seattle; 37 Department of Systems, Populations, and Leadership, University of Michigan, Ann Arbor; 38 Department of Pediatrics, Ohio State University, Columbus; 39 Department of Pediatric Neurology, Nationwide Children’s Hospital, Columbus, Ohio; 40 Department of Basic Medical Sciences, Neuroscience and Sense Organs, University of Bari Aldo Moro, Bari, Italy; 41 Department of Clinical Research in Neurology, Fondazione Cardinale Giovanni Panico Hospital, Tricase, Italy; 42 Center for Translation Research and Implementation Science, National Institutes of Health, Bethesda, Maryland; 43 Department of Medicine, University of Cape Town, Cape Town, South Africa; 44 Pacific Institute for Research & Evaluation, Calverton, Maryland; 45 School of Public Health, Curtin University, Perth, Australia; 46 Department of Biomolecular Sciences, University of Mississippi, Oxford; 47 Department of Pharmacy, Mizan-Tepi University, Mizan, Ethiopia; 48 Preventive Medicine and Public Health Research Center, Iran University of Medical Sciences, Tehran, Iran; 49 Section of Plastic Surgery, University of Michigan, Ann Arbor; 50 Department of Neurology, Emory University, Atlanta, Georgia; 51 Department of Clinical Sciences, Lund University, Lund, Sweden; 52 University of Central Florida College of Medicine, Orlando; 53 Division of Cardiology, University of Washington, Seattle; 54 Department of Cardiology, Charité Medical University Berlin, Berlin, Germany; 55 Center for Stroke Research Berlin, Berlin, Germany; 56 Centre for Medical Informatics, University of Edinburgh, Edinburgh, United Kingdom; 57 Division of General Internal Medicine, Harvard University, Boston, Massachusetts; 58 Yonsei University College of Medicine, Seoul, South Korea; 59 The University of Alabama at Birmingham School of Medicine, Birmingham; 60 Medicine Service, US Department of Veterans Affairs, Birmingham, Alabama; 61 Department of Neuromedicine and Movement Science, Norwegian University of Science and Technology, Trondheim, Norway; 62 Division of Brain Sciences, Imperial College London, London, United Kingdom; 63 Department of Neurology and Clinical Neurophysiology, St Olavs Hospital, Trondheim, Norway; 64 Department of Neurology, George Washington University, Washington, DC; 65 University of Maryland School of Medicine, Baltimore; 66 Department of Demography, University of California, Berkeley, Berkeley; 67 Global Health Research Center, Duke Kunshan University, Kunshan, China; 68 Duke Global Health Institute, Duke University, Durham, North Carolina

## Abstract

**Importance:**

Accurate and up-to-date estimates on incidence, prevalence, mortality, and disability-adjusted life-years (burden) of neurological disorders are the backbone of evidence-based health care planning and resource allocation for these disorders. It appears that no such estimates have been reported at the state level for the US.

**Objective:**

To present burden estimates of major neurological disorders in the US states by age and sex from 1990 to 2017.

**Design, Setting, and Participants:**

This is a systematic analysis of the Global Burden of Disease (GBD) 2017 study. Data on incidence, prevalence, mortality, and disability-adjusted life-years (DALYs) of major neurological disorders were derived from the GBD 2017 study of the 48 contiguous US states, Alaska, and Hawaii. Fourteen major neurological disorders were analyzed: stroke, Alzheimer disease and other dementias, Parkinson disease, epilepsy, multiple sclerosis, motor neuron disease, migraine, tension-type headache, traumatic brain injury, spinal cord injuries, brain and other nervous system cancers, meningitis, encephalitis, and tetanus.

**Exposures:**

Any of the 14 listed neurological diseases.

**Main Outcome and Measure:**

Absolute numbers in detail by age and sex and age-standardized rates (with 95% uncertainty intervals) were calculated.

**Results:**

The 3 most burdensome neurological disorders in the US in terms of absolute number of DALYs were stroke (3.58 [95% uncertainty interval [UI], 3.25-3.92] million DALYs), Alzheimer disease and other dementias (2.55 [95% UI, 2.43-2.68] million DALYs), and migraine (2.40 [95% UI, 1.53-3.44] million DALYs). The burden of almost all neurological disorders (in terms of absolute number of incident, prevalent, and fatal cases, as well as DALYs) increased from 1990 to 2017, largely because of the aging of the population. Exceptions for this trend included traumatic brain injury incidence (−29.1% [95% UI, −32.4% to −25.8%]); spinal cord injury prevalence (−38.5% [95% UI, −43.1% to −34.0%]); meningitis prevalence (−44.8% [95% UI, −47.3% to −42.3%]), deaths (−64.4% [95% UI, −67.7% to −50.3%]), and DALYs (−66.9% [95% UI, −70.1% to −55.9%]); and encephalitis DALYs (−25.8% [95% UI, −30.7% to −5.8%]). The different metrics of age-standardized rates varied between the US states from a 1.2-fold difference for tension-type headache to 7.5-fold for tetanus; southeastern states and Arkansas had a relatively higher burden for stroke, while northern states had a relatively higher burden of multiple sclerosis and eastern states had higher rates of Parkinson disease, idiopathic epilepsy, migraine and tension-type headache, and meningitis, encephalitis, and tetanus.

**Conclusions and Relevance:**

There is a large and increasing burden of noncommunicable neurological disorders in the US, with up to a 5-fold variation in the burden of and trends in particular neurological disorders across the US states. The information reported in this article can be used by health care professionals and policy makers at the national and state levels to advance their health care planning and resource allocation to prevent and reduce the burden of neurological disorders.

## Key Points

**Question:**

What is the current burden of neurological disorders in the US by states, and what are the temporal trends (from 1990 to 2017)?

**Findings:**

Systematic analysis of the Global Burden of Disease study shows that, in 2017, the 3 most burdensome neurological disorders in the US were stroke, Alzheimer disease and other dementias, and migraine. The burden of individual neurological disorders varied moderately to widely by states (a 1.2-fold to 7.5-fold difference), and the absolute numbers of incident, prevalent, and fatal cases and disability-adjusted life-years of neurological disorders (except for traumatic brain injury incidence; spinal cord injury prevalence; meningitis prevalence, deaths, and disability-adjusted life-years; and encephalitis disability-adjusted life-years) across all US states increased from 1990 to 2017.

**Meaning:**

A large and increasing number of people have various neurological disorders in the US, with significant variation in the burden of and trends in neurological disorders across the US states, and the reasons for these geographic variations need to be explored further.

## Teaser

This analysis of the Global Burden of Disease 2017 study presents burden estimates of major neurological disorders in the US states by age and sex from 1990 to 2017.

## Introduction

According to Gooch et al,^1^ in 2011, nearly 100 million Americans were affected by at least 1 of the more than 1000 neurological disorders. This translates into an overall cost of $765 billion for the more prevalent conditions,^1^ including Alzheimer disease (AD) and other dementias, chronic low back pain, stroke, migraine, epilepsy, traumatic brain injury (TBI), and Parkinson disease (PD). Although accurate data on incidence, prevalence, mortality, and disability from neurological disorders and their trends are important for evidence-based health care planning and resource allocation, there is a lack of national and state-level epidemiological data in the US, as well as their synthesis for use by health care planners.

Previous Global Burden of Diseases, Injuries, and Risk Factors (GBD) Study articles have reported burden of diseases, injuries, and risk factors among US states^2^ and global, regional, and country-specific estimates of the burden from neurological disorders,^3,4^ but no estimates for a comprehensive list of neurological disorders have been reported for the US states from this study. Globally, regionally, and at the country level of analysis, the burden of all noncommunicable neurological disorders, in terms of the absolute number of people who had, remained disabled by, or died from them, has increased significantly across all countries in the world, but the burden from communicable neurological disorders, such as tetanus, meningitis, and encephalitis, has decreased from 1990 to 2016.^5,6,7,8,9,10,11,12,13,14^ This study provides estimates for noncommunicable and communicable neurological disorders at the state level from 1990 to 2017.

## Methods

Details on the methodology of the GBD study overall^15^ and in association with the burden of neurological disorders have been published elsewhere.^5,6,7,8,9,10,11,12,13,14^ In brief, using statistical modeling techniques, the GBD study allows use of all available epidemiological data (both published and unpublished) and routinely collected data (eg, vital registration, hospitalizations, medical claims) within and just outside of the country, region, or state of interest to provide the most accurate estimates of the disease burden, even for regions or states with no accurate epidemiological data.^15^ The burden estimates are presented in terms of age-adjusted annual rates per 100 000 people and numbers of prevalence, incidence, disability-adjusted life-years (DALYs), and deaths by cause (in millions), with corresponding 95% uncertainty intervals (UIs), and their trends or changes from 1990 to 2017.

Nonfatal estimates were obtained from systematic reviews, surveys, administrative health records, registries, and disease surveillance systems. We used available surveys and databases of claims information for US private and public insurance schemes, corrected for bias in health service encounters, as described elsewhere.^2,16,17^ Data sources used for quantifying nonfatal outcomes are available online in the GBD results tool.^18^ Fatal estimates were obtained from vital registration data (death records from the National Center for Health Statistics and population counts from the US Census Bureau). The single cause of death was determined using the *International Classification of Diseases, Ninth Revision, Ninth Revision, Clinical Modification; International Statistical Classification of Diseases and Related Health Problems, Tenth Revision*; and *International Statistical Classification of Diseases, Tenth Revision, Clinical Modification*, with redistributions from less precise codes (such as ill-defined disease [R99] or injuries of undetermined intent [Y21-Y33]) to more specific diseases and injuries using regression methods of the subset of vital registration data for which we have multiple causes of death information. Under *International Classification of Diseases* rules, injury deaths are classified by the cause of injury (eg, fall or road injury) and not the nature of injury, such as TBI and spinal cord injuries (SCI). Thus, we report on incidence, prevalence, and years lived with disability for these causes, but not mortality. Causes of death data were analyzed using the Cause of Death Ensemble model (CODEm,^19^ with corrections for changes in coding practices for underlying causes of death as explained in detail elswhere^20^), and nonfatal data were analyzed using DisMod-MR version 2.1 (World Health Organization),^21^ a bayesian meta-regression tool that adjusts data points for variations in study methods among different data sources and enforces consistency between prevalence, incidence, and mortality. For each disease and injury, years lived with disability for mutually exclusive sequelae (ie, disabling outcomes) were quantified as the product of prevalence and a weighting for severity (with the GBD disability weights). Values for disability weights have been derived from population surveys in 9 countries and an open-access internet survey.^22^ To account for co-occurrence of disease and injury outcomes, years lived with disability were corrected for comorbidity, assuming a multiplicative rather than additive function of disability weights.^23,24^

To allow comparison with the GBD estimates on the global burden of neurological disorders,^3^ 14 neurological disorders that are quantified as part of GBD are included in this report. These are stroke, AD and other dementias, PD, idiopathic epilepsy (ie, epilepsy that is not secondary to any of the other GBD causes), multiple sclerosis (MS), motor neuron disease (MND), migraine, tension-type headache (TTH), TBI, SCI, brain and other central nervous system (CNS) cancers, meningitis, encephalitis, and tetanus.

## Results

### Burden of Neurological Disorders in the US

Among the neurological disorders, the 5 most prevalent were TTH (121.6 [95% UI, 110-133] million people), migraine (68.5 [95% UI, 64-73] million people), stroke (7.8 [95% UI, 7.4-8.2] million people), AD and other dementias (2.9 [95% UI, 2.6-3.2] million people), and SCI (2.2 [95% UI, 2.0-2.3] million people) (Table 1), while the most burdensome in terms of DALYs were stroke (3.6 [95% UI, 3.3-3.9] million DALYs), AD and other dementias (2.6 [95% UI, 2.4-2.7] million DALYs), migraine (2.4 [95% UI, 1.5-3.4] million DALYs), idiopathic epilepsy (0.4 [95% UI, 0.3-0.6] million people), and PD (0.4 [95% UI, 0.3-0.4] million people). The 5 leading causes of death from neurological disorders were from AD and other dementias (258 600 [95% UI, 254 000-263 000] deaths), stroke (172 000 [95% UI, 166 000-178 000] deaths), PD (30 000 [95% UI, 24 000-31 000] deaths), MND (8400 [95% UI, 8000-9000] deaths), and MS (4000 [95% UI, 3000-4000] deaths). The highest incidence was of new-onset TTH (44.5 [95% UI, 40.0-48.8] million cases per year) followed by migraine (5.0 [95% UI, 4.6-5.5] million cases per year), TBI (0.96 [95% UI, 0.8-1.2] million cases per year), stroke (0.60 [95% UI, 0.55-0.65] million cases per year), and AD and other dementias (0.48 [0.47-0.57] million cases per year).

**Table 1.** Millions of Incident, Prevalent, and Fatal Cases and Disability-Adjusted Life-Years (DALYs) of Neurological Disorders in the US in 1990 and 2017, With Percentage Changes From 1990 to 2017

Disease	Incident cases (95% UI)			Prevalent cases (95% UI)			Mortality rates (95% UI)			DALY cases (95% UI)		
	1990	2017	Change, %	1990	2017	Change, %	1990	2017	Change, %	1990	2017	Change, %
Stroke	0.435 (0.404 to 0.467)	0.601 (0.554 to 0.653)	38.2 (37.1 to 39.8)	4.72 (4.465 to 4.984)	7.778 (7.386 to 8.209)	64.8 (64.7 to 65.4)	0.145 (0.143 to 0.148)	0.172 (0.166 to 0.178)	18.8 (16.5 to 20.6)	2.913 (2.712 to 3.11)	3.584 (3.251 to 3.922)	23.1 (19.8 to 26.1)
Alzheimer disease and other dementias	0.347 (0.302 to 0.399)	0.516 (0.471 to 0.565)	48.4 (41.5 to 55.8)	1.933 (1.663 to 2.222)	2.884 (2.637 to 3.149)	49.2 (41.7 to 58.5)	0.125 (0.123 to 0.127)	0.259 (0.254 to 0.263)	106.4 (105.8 to 108)	1.477 (1.39 to 1.572)	2.553 (2.43 to 2.679)	72.8 (70.4 to 74.8)
Parkinson disease	0.036 (0.029 to 0.044)	0.072 (0.063 to 0.082)	98.3 (85.3 to 114.2)	0.29 (0.234 to 0.356)	0.55 (0.481 to 0.615)	89.9 (72.8 to 105.8)	0.013 (0.013 to 0.016)	0.03 (0.024 to 0.031)	123 (88 to 94.6)	0.204 (0.188 to 0.238)	0.412 (0.346 to 0.442)	102 (84.7 to 85.6)
Idiopathic epilepsy	0.099 (0.07 to 0.129)	0.147 (0.101 to 0.191)	48 (43.9 to 48.7)	0.929 (0.653 to 1.219)	1.415 (0.992 to 1.851)	52.3 (51.8 to 51.8)	0.002 (0.002 to 0.002)	0.002 (0.002 to 0.002)	37.1 (33.6 to 38.8)	0.304 (0.2 to 0.44)	0.416 (0.252 to 0.625)	36.8 (25.9 to 41.9)
Multiple sclerosis	0.008 (0.007 to 0.009)	0.01 (0.009 to 0.01)	23.9 (19.3 to 27.8)	0.244 (0.221 to 0.272)	0.392 (0.368 to 0.417)	60.4 (53.6 to 66.8)	0.002 (0.002 to 0.002)	0.004 (0.003 to 0.004)	126.3 (69.7 to 89.4)	0.115 (0.096 to 0.137)	0.204 (0.168 to 0.235)	76.6 (71.8 to 75)
Motor neuron disease	0.005 (0.005 to 0.005)	0.01 (0.009 to 0.01)	86.8 (85.7 to 87.6)	0.025 (0.023 to 0.028)	0.038 (0.035 to 0.041)	50.4 (47 to 53.5)	0.004 (0.004 to 0.004)	0.008 (0.008 to 0.009)	129.3 (124.8 to 132.4)	0.1 (0.097 to 0.103)	0.199 (0.189 to 0.207)	99.2 (95.3 to 101.3)
Migraine	4.183 (3.808 to 4.549)	5.037 (4.629 to 5.458)	20.4 (20 to 21.6)	53.317 (49.613 to 57.486)	68.487 (63.84 to 73.437)	28.5 (27.7 to 28.7)	NA	NA	NA	1.86 (1.191 to 2.692)	2.404 (1.535 to 3.441)	29.3 (27.8 to 28.9)
Tension-type headache	34.088 (30.244 to 37.782)	44.471 (39.999 to 48.819)	30.5 (29.2 to 32.3)	92.316 (83.394 to 102.183)	121.60 (110.40 to 133.34)	31.7 (30.5 to 32.4)	NA	NA	NA	0.261 (0.147 to 0.416)	0.35 (0.199 to 0.555)	33.8 (33.4 to 35.3)
Traumatic brain injury	1.005 (0.845 to 1.198)	0.961 (0.795 to 1.163)	−4.4 (−5.9 to −2.9)	1.973 (1.886 to 2.057)	2.104 (2.01 to 2.197)	6.6 (6.6 to 6.8)	NA	NA	NA	NA	NA	NA
Spinal cord injury	0.075 (0.061 to 0.094)	0.075 (0.059 to 0.097)	0.2 (−2.6 to 4.1)	2.404 (2.188 to 2.67)	2.159 (2.008 to 2.327)	−10.2 (−12.8 to −8.2)	NA	NA	NA	NA	NA	NA
Brain and other nervous system cancers	0.016 (0.014 to 0.018)	0.029 (0.026 to 0.032)	77.2 (82.6 to 82.8)	0.058 (0.052 to 0.064)	0.112 (0.104 to 0.131)	92.7 (101 to 106)	0.011 (0.01 to 0.012)	0.018 (0.016 to 0.02)	55.6 (55 to 57.9)	0.358 (0.311 to 0.385)	0.49 (0.449 to 0.558)	36.8 (44.1 to 44.9)
Meningitis	0.049 (0.043 to 0.056)	0.05 (0.044 to 0.056)	1.8 (0.7 to 2.1)	0.11 (0.096 to 0.128)	0.089 (0.078 to 0.102)	−19.2 (−20 to −17.9)	0.003 (0.002 to 0.003)	0.001 (0.001 to 0.002)	−48.5 (−42.3 to −41.1)	0.147 (0.129 to 0.158)	0.063 (0.059 to 0.075)	−56.9 (−54.6 to −52.7)
Encephalitis	0.014 (0.013 to 0.014)	0.019 (0.019 to 0.02)	41.9 (41.8 to 42)	0.02 (0.012 to 0.032)	0.026 (0.015 to 0.041)	29.3 (28.5 to 30)	0.001 (0.001 to 0.001)	0.001 (0.001 to 0.001)	22.1 (29.4 to 37)	0.026 (0.023 to 0.028)	0.025 (0.024 to 0.031)	−3.1 (2.9 to 10.7)

Abbreviations: NA, not available; UI, uncertainty interval.

While the US-wide age-standardized incidence, prevalence, mortality, and DALY rates of most neurological disorders declined or remained flat from 1990 through 2017 (Table 2), the absolute number of incident cases, prevalent cases, mortality, and DALYs increased, except for meningitis and encephalitis (Table 1). In 2017, the 5 highest incidence rates were for TTH (13 014 [95% UI, 11 602-14 432] cases per 100 000 people), migraine (1722 [95% UI, 1578-1865] cases per 100 000 people), TBI (285 [95% UI, 238-341] cases per 100 000 people), stroke (115 [95% UI, 107-125] cases per 100 000 people), and AD and other dementias (85 [95% UI, 78-93] cases per 100 000 people). Prevalence was highest for TTH (34 642 [95% UI, 31 341-38 113] cases per 100 000 people), migraine (20 188 [95% UI, 18 678-21 750] cases per 100 000 people), stroke (1536 [95% UI, 1461-1621] cases per 100 000 people), SCI (541 [95% UI, 502-583] cases per 100 000 people), and TBI (502 [95% UI, 478-525] cases per 100 000 people). The 2 leading causes of mortality were AD and other dementias (38 [95% UI, 38-39] per 100 000 population per year) and stroke (29 [95% UI, 28-30] per 100 000 population per year). The 5 leading causes of DALYs (rates) were migraine (705 [95% UI, 446-1021] per 100 000 population per year), stroke (692 [95% UI, 625-759] per 100 000 population per year), AD and other dementias (419 [95% UI, 399-439] per 100 000 population per year), idiopathic epilepsy (124 [95% UI, 75-187] per 100 000 population per year), and brain and other nervous system cancers (120 [95% UI, 111-138] per 100 000 population per year).

**Table 2.** Age-Standardized Incidence, Prevalence, Mortality, and Disability-Adjusted Life-Year (DALY) Rates (per 100 000 People), for Neurological Disorders in the US in 1990 and 2017 and the Percentage Change From 1990 to 2017

Disease	Incidence rates (95% UI)			Prevalence (95% UI)			Mortality rates (95% UI)			DALY rates (95% UI)		
	1990	2017	Change, %	1990	2017	Change, %	1990	2017	Change, %	1990	2017	Change, %
Stroke	137.5 (127.9 to 147.6)	115 (106.5 to 125)	−16.3 (−19.1 to −13.8)	1539.4 (1459 to 1623)	1537 (1461 to 1621)	−0.2 (−3.8 to 3.3)	42 (41.4 to 42.9)	28.6 (27.6 to 29.5)	−32.0 (−34.5 to −29.8)	910.4 (845.5 to 973.9)	691.9 (624.6 to 759.3)	−24.0 (−26.9 to −21.3)
Alzheimer disease and other dementias	97.2 (85 to 111)	85.2 (77.8 to 93)	−12.4 (−19.2 to −5.2)	542.7 (468.9 to 622.1)	470 (429.1 to 513.8)	−13.4 (−20.6 to −5.1)	35 (34.5 to 35.4)	38.5 (37.7 to 39.2)	9.8 (7.3 to 12.2)	413.6 (389.2 to 439.7)	418.8 (398.8 to 439.1)	1.2 (−1.9 to 4.2)
Parkinson disease	10.5 (8.7 to 12.8)	12.9 (11.2 to 14.6)	21.9 (8.3 to 37)	83.5 (67.7 to 101.9)	97 (84.5 to 108.6)	16.2 (2.7 to 31)	3.7 (3.6 to 4.4)	4.9 (4 to 5.1)	33.1 (−4.6 to 41.7)	58.1 (53.4 to 67.9)	72.5 (61 to 78)	24.8 (−5.2 to 32.9)
Idiopathic epilepsy	40.6 (28.7 to 52.8)	47.6 (32.3 to 62.1)	17.4 (−6 to 45.1)	357.3 (252.4 to 469.5)	411.8 (290.2 to 540.5)	15.2 (−7.5 to 43.3)	0.6 (0.6 to 0.6)	0.5 (0.5 to 0.6)	−2.7 (−7.8 to 1.5)	117.4 (76.9 to 172.7)	123.9 (75.1 to 187.5)	5.5 (−18.4 to 33.2)
Multiple sclerosis	2.9 (2.7 to 3.2)	3.3 (3.1 to 3.5)	13.2 (7.3 to 18.9)	85.1 (76.8 to 94.6)	97.6 (91.5 to 103.7)	14.7 (8.1 to 21.3)	0.6 (0.6 to 0.8)	0.8 (0.6 to 0.9)	32.8 (−20.1 to 56.1)	40.5 (33.7 to 47.7)	47.2 (39.3 to 54.7)	16.6 (−1.4 to 26.2)
Motor neuron disease	1.7 (1.6 to 1.8)	1.9 (1.8 to 2.1)	12.5 (9.8 to 15.3)	9 (8.1 to 10)	9.1 (8.4 to 9.8)	0.3 (−4.4 to 5.6)	1.2 (1.1 to 1.2)	1.6 (1.5 to 1.7)	38.3 (30 to 45.6)	35.5 (34.3 to 36.8)	42.9 (40.7 to 44.7)	20.9 (13.9 to 27.6)
Migraine	1696 (1544 to 1836)	1722 (1578 to 1865)	1.6 (−0.8 to 4.2)	19 902 (18 489 to 21 465)	20 188 (18 678 to 21 750)	1.4 (−0.8 to 4)	NA	NA	NA	694.1 (443.6 to 1008)	704.8 (446 to 1020.5)	1.5 (−0.8 to 4)
Tension-type headache	12 965 (11 495 to 14 380)	13 014 (11 602 to 14 432)	0.4 (−2.1 to 2.9)	34 469 (31 156 to 38 223)	34 642 (31 341 to 38 113)	0.5 (−2 to 3.3)	NA	NA	NA	96.5 (54.4 to 154.3)	96.6 (54.4 to 154.9)	0.1 (−1.5 to 1.9)
Traumatic brain injury	402 (339 to 482)	285 (238 to 341)	−29.1 (−32.4to −25.8)	700 (668 to 731)	502 (478 to 525)	−28.3 (−29.7 to −26.9)	NA	NA	NA	NA	NA	NA
Spinal cord injury	30 (24 to 37)	21 (16 to 26)	−30.5 (−34.8 to −26.2)	879 (798 to 981)	541 (502 to 583)	−38.5 (−43.1 to −34.0)	NA	NA	NA	NA	NA	NA
Brain and nervous system cancers	5.8 (5.1 to 6.3)	7.2 (6.6 to 8.2)	24.1 (12.4 to 41.4)	22.5 (20 to 24.8)	34.2 (31.7 to 40.1)	52.2 (36.7 to 76.5)	3.8 (3.3 to 4.1)	3.7 (3.3 to 4.1)	−3.4 (−9.2 to 6.8)	131.2 (114.7 to 141.4)	119.9 (111.2 to 138)	−8.6 (−15.5 to 5.4)
Meningitis	19.4 (17 to 22.4)	13.8 (12.2 to 15.6)	−28.7 (−32.9 to −24.4)	41 (35.6 to 47.3)	22.7 (20 to 25.9)	−44.8 (−47.3 to −42.3)	1.1 (0.9 to 1.2)	0.4 (0.4 to 0.5)	−64.4 (−67.7 to −50.3)	64.9 (56.7 to 70)	21.5 (19.7 to 25.7)	−66.9 (−70.1 to −55.9)
Encephalitis	5 (4.9 to 5.1)	5 (4.9 to 5.1)	−0.1 (−0.7 to 0.6)	7.1 (4.2 to 11.1)	6.4 (3.9 to 9.8)	−10.1 (−12 to −8.1)	0.2 (0.2 to 0.2)	0.2 (0.2 to 0.2)	−20.6 (−24.9 to −1.7)	10.4 (9.2 to 11)	7.7 (7.2 to 9.5)	−25.8 (−30.7 to −5.8)

Abbreviations: NA, not available; UI, uncertainty interval.

**Figure 1.**
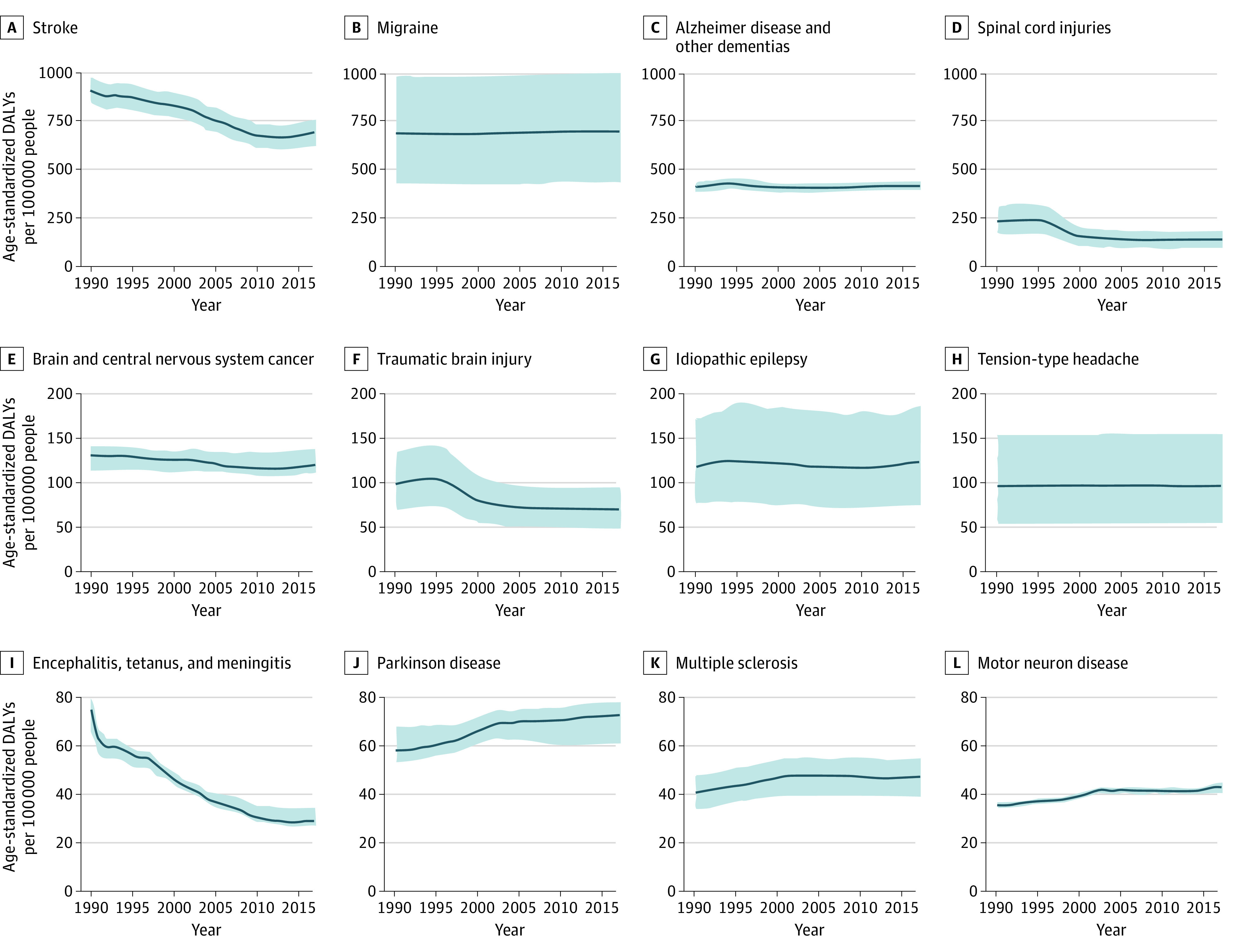
Temporal Trends in Aggregate US-Wide Age-Standardized Disability-Adjusted Life-Year (DALY) Rates per 100 000 Persons per Year for Neurological Disorders From 1990 to 2017

For stroke, from 1990 to 2017, there were significant reductions in age-standardized incidence (−16.3% [95% UI, −19.1% to −13.8%]), mortality (−32.0% [95% UI, −34.5% to −29.8%]), and DALY (−24.0% [95% UI, −26.9% to −21.3]) rates, but no significant change in age-standardized prevalence (0.2% [95% UI, −3.8% to 3.3%]) (Table 2). Age-standardized DALY rates of stroke stopped declining and plateaued around 2010, with some nonsignificant trends to increase since 2015 (Figure 1; eFigure in the Supplement). Age-standardized incidence and prevalence of AD and other dementias decreased (−12.4% [95% UI, −19.2% to −5.2%] and −13.4% [95% UI, −20.6% to −5.1%], respectively), but mortality and DALY rates increased by 9.8% (95% UI, 7.3%-12.2%) and 1.2% (95% UI, −1.9% to 4.2%), respectively. For TBI and SCI, there were also significant decreases in age-standardized incidence (−29.1% [95% UI, −32.4% to −25.8%] and −30.5% [95% UI, −34.8% to −26.2%], respectively) and prevalence (−28.3% [95% UI, −29.7% to −26.9%] and −38.5% [95% UI, −43.1% to −34.0%], respectively). Although age-standardized mortality and DALY rates from brain and other CNS cancers decreased (−3.4% [95% UI, −9.2% to 6.8%] and −8.6% [95% UI, −15.5% to 5.4%], respectively), incidence and prevalence rates increased significantly (24.1% [95% UI, 12.4%-41.4%] and 52.2% [95% UI, 36.7%-76.5%], respectively). In meningitis, there were significant reductions in the age-standardized rates of incidence (−28.7% [95% UI, −32.9% to −24.4%]), prevalence (−44.8% [95% UI, −47.3% to −42.3%]), mortality (−64.4% [95% UI, −67.7% to −50.3%]), and DALY (−66.9% [95% UI, −70.1% to −55.9%]). In encephalitis, rates of incidence (−0.1% [95% UI, −0.7% to 0.6%]), prevalence (−10.1% [95% UI, −12% to −8.1%]), mortality (−20.6% [95% UI, −24.9% to −1.7%]), and DALY (−25.8% [95% UI, −30.7% to −5.8%]) also decreased. In PD, there were small but significant increases in the age-standardized rates of incidence (21.9% [95% UI, 11.2%-14.6%]), prevalence (16.2% [95% UI, 2.7%-31%]), mortality (33.1% [95% UI, −4.6% to 41.7%]), and DALY (24.8% [95% UI, −5.2% to 32.9%]) of PD. Age-standardized incidence (17.4% [95% UI, −6% to 45.1%]), prevalence (15.2% [95% UI, −7.5% to 43.3%]), and DALY (5.5% [95% UI, −18.4% to 33.2%]) rates of epilepsy increased, but mortality rates decreased (−2.7% [95% UI, −7.8% to 1.5%]). There was an increase in the age-standardized incidence, prevalence, mortality, and DALY rates of MS (13.2% [95% UI, 7.3%-18.9%], 14.7% [95% UI, 8.1%-21.3%], 32.8% [95% UI, −20.1% to 56.1%], and 16.6% [95% UI, −1.4% to 26.2%], respectively) and MND (12.5% [95% UI, 9.8%-15.3%], 0.3% [95% UI, −4.4% to 5.6%], 38.3% [95% UI, 30%-45.6%], and 20.9% [95% UI, 13.9%-27.6%], respectively). There were small increases in the incidence, prevalence, and DALY age-standardized rates of migraine (1.6% [95% UI, −0.8% to 4.2%], 1.4% [95% UI, −0.8% to 4%], and 1.5% [95% UI, −0.8% to 4%], respectively) and TTH (0.4% [95% UI, −2.1% to 2.9%], 0.5% [95% UI, −2% to 3.3%], and 0.1% [95% UI, −1.5% to 1.9%], respectively).

However, the absolute numbers of people affected by noncommunicable neurological disorders (Table 1) in terms of incident, prevalent, and fatal cases, as well as DALYs, have increased substantially from 1990 to 2017 (except with respect to TBI incident and SCI prevalent cases, where small, nonsignificant reductions were observed). The largest increases were observed for (in order of increase) PD, MND, AD and other dementias, brain and other CNS cancers, and stroke.

### Burden of Selected Neurological Disorders in US States

**Figure 2.**
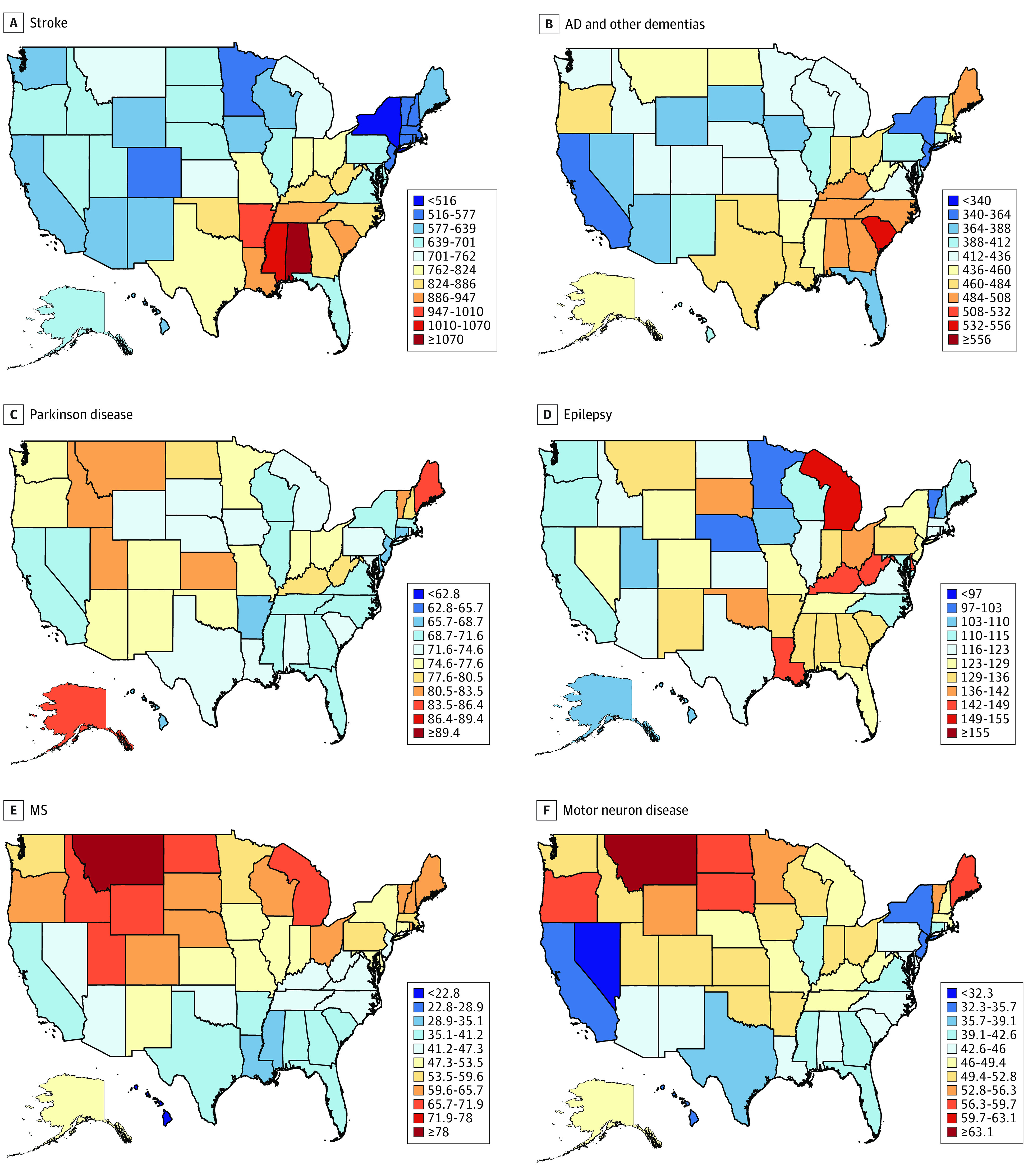
Disability-Adjusted Life-Year Rates per 100 000 Persons for Neurological Disorders in the US States in 2017 MS indicates multiple sclerosis.

**Figure 3.**
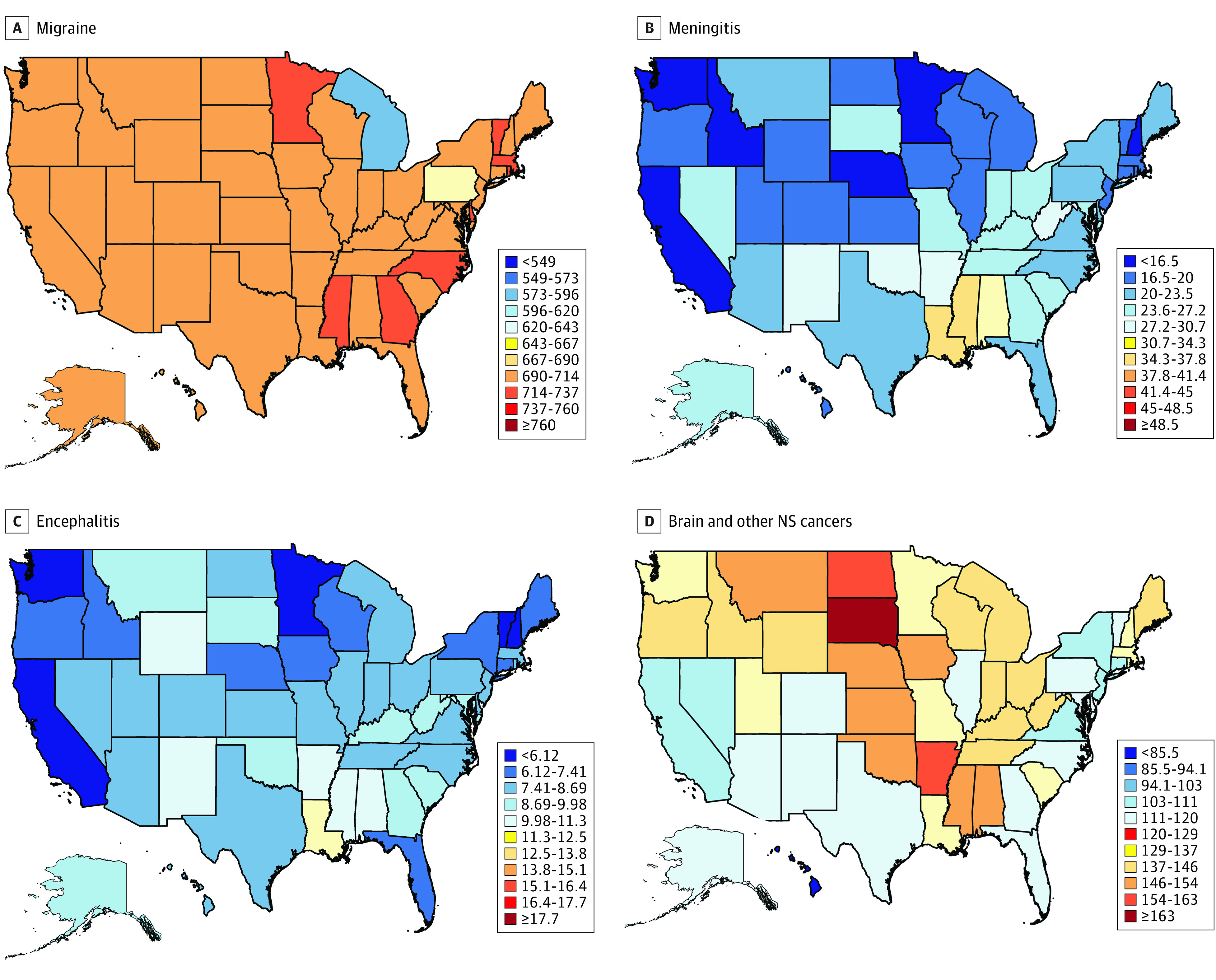
Disability-Adjusted Life-Year Rates per 100 000 Persons for Neurological Disorders in the US States in 2017 NS indicates nervous system.

Figure 2 and Figure 3 show age-standardized incidence, prevalence, mortality, and DALY rates of the selected neurological disorders in the US states in 2017. Age-standardized prevalence, incidence, mortality, and DALY rates for specific neurological disorders in the US states and changes in the rates from 1990 to 2017 and the US states’ ranking based on the 2017 estimates are described and shown in eTables 1 through 13 in the Supplement.

The different metrics of age-standardized rates varied between the US states from a 1.2-fold difference for TTH to 7.5-fold for tetanus. Southeastern states and Arkansas had a relatively higher burden for stroke, northern states had a relatively higher burden of MS, and eastern states had higher rates of PD, idiopathic epilepsy, migraine, TTH, and meningitis, encephalitis, and tetanus (eFigure, eAppendix, and eTables 1 through 12 in the Supplement).

### Changes in the Burden From 1990 to 2017

From 1990 to 2017, the direction of trends in changes in the age-standardized incidence, prevalence, mortality, and DALY rates across US states mirrored those for the country as a whole. Over that period of time, there were very few changes in any of the states in the rates of migraine and TTH.

## Discussion

To our knowledge, this is the first comprehensive report on incidence, prevalence, mortality, and DALY estimates and their trends from 1990 to 2017 for 14 neurological disorders for the individual 50 US states and the country overall. The study showed reductions in the age-adjusted rates of most burden metrics of stroke, AD and other dementias, TBI, SCI, meningitis, and encephalitis, but increasing numbers of people affected by various neurological disorders in the US, with a significant (up to 5-fold) variation in the burden of and trends in particular neurological disorders across the US states. Falling rates of stroke, AD and other dementias, TBI, SCI, meningitis, and encephalitis might suggest that primary prevention of these disorders are beginning to show an influence, while between-state variations may be associated with differences in the case ascertainment, as well as access to health care; racial/ethnic, genetic, and socioeconomic diversity; quality and comprehensiveness of preventive strategies; and risk factor distribution. For dementia, improving educational levels of cohort reaching the age groups at greatest risk of disease may also be contributing to a modest decline over time.^25^ While globally, 6 neurological disorders (AD and other dementias, PD, epilepsy, MS, MND, and headache disorders) in 2017 constituted 4.4% (95% UI, 3.7%-5.3%)^26^ of total DALYs and ranked ninth among the leading causes of DALYs, in the US, they constituted 6.7% (95% UI, 6.0%-7.6%) and ranked fifth. In 2017, deaths from these neurological disorders were ranked fifth (5.5% [95% UI, 5.4%-5.6%]) among all causes of death globally and third (10.8% [95% UI, 10.6%-10.9%]) in the US.^26^ Similar findings were observed for neurological disorders in Western European countries.^3^ Given the association of prevalent disorders, such as AD, stroke, PD, and MND with older age, the higher rank of neurological disorders in the US can be explained by the longer life expectancy in the US compared with the world overall. In addition, it may also be attributable to better case ascertainment in the US, including improved diagnosis, surveillance or reporting, and health care access.

The exact causes for significant between-state variations in the age-standardized rates of some neurological disorders are unknown, but these may be associated with between-state differences in the completeness of medical examinations and accuracy of diagnosis, completeness and accuracy of data from medical claims, and referral patterns to specialized centers (eg, seeking medical advice for brain tumors outside of the place of residence), despite corrections for these measurement biases during data analysis. The highest between-state variations were observed for tetanus, but the number of tetanus cases was very low, and thus large relative variation reflected variation by just 1 or a few cases. However, our data on between-state variations in the burden from neurological disorders concur with previous GBD observations of large differences in the burden of disease among US states^2^ and significant geographical variations in the burden of neurological disorders in the world.^3^ For example, for stroke, there is a strong increasing gradient from north to south, while for MS, the gradient is decreasing from north to south. Both gradients might have been expected based on our understanding of the epidemiology of both diseases.^27,28,29^ While confirming previous observations of the so-called stroke belt mortality in the southeastern United States, unlike previous estimates that identified North Carolina, South Carolina, and Georgia with a higher stroke mortality rate than the other states of the stroke belt,^30,31^ our 2017 GBD stroke mortality estimates show that the stroke belt is now in Alabama, Mississippi, and South Carolina. Our dementia mortality estimates also showed a higher percentage change (increase) in the stroke belt states compared with the other states, likely attributable to the reciprocal association between stroke and dementia.^32^ These findings can be used to set research priorities (eg, identifying causes of between-state variations in the burden of neurological disorders). These data will also allow health care professionals and policy makers on national and state levels to allocate resources (eg, number of hospital beds, specialists, services) and give priorities for improving care for people with some major neurological disorders in each US state.

The finding that the 3 most burdensome neurological disorders in the US in terms of the absolute numbers of DALYs are stroke, AD and other dementias, and migraine is in line with ranking of age-standardized DALY rates for neurological disorders found in other high-income countries in 2016.^3^ There were diverse changes in the rates of all neurological disorders in the country overall (eg, decreased incidence rates of stroke, AD and other dementias, TBI, SCI, and meningitis, and increased incidence rates of PD, MS, MND, and brain and other nervous system cancers) and across all US states from 1990 to 2017. However, the absolute number of people who are affected by noncommunicable neurological disorders over that period has increased substantially and is likely to continue increasing because of aging of the US population and population growth. Unfavorable trends in some lifestyle factors (eg, overweight, fasting plasma glucose level)^2^ in the US are also likely additional contributing factors for the increasing burden from some neurological disorders (eg, stroke, dementia).^33^ These developments are consistent with the global trends^3^ and support the call to action to reduce the burden of neurological disorders in the United States, as outlined by Gooch et al.^1^

Although we understand that individuals may have more than 1 neurological condition, assuming no overlap between stroke, TBI, SCI, brain and other CNS cancers, meningitis, encephalitis, tetanus, and other neurological disorders (AD and other dementias, PD, idiopathic epilepsy, MS, MND, migraine, TTH, and other neurological disorders), it can be estimated that in 2017 more than 200 million Americans (60% of the population) were afflicted by at least 1 neurological disorder, ranging from TTH and migraine to stroke and dementia. This is twice the estimate by Gooch et al^1^ and 9 times the estimate by Borlongan et al.^34^ The greater than previously reported overall prevalence of neurological disorders in the US may be explained by at least 3 factors. First, we included many disorders in this study that Gooch et al^1^ did not, such as TTH (the most prevalent of all neurological disorders analyzed in the GBD study), brain and other CNS cancers, tetanus, meningitis, and encephalitis, although they^1^ did include chronic low back pain, which we did not. The selection of neurological disorders included in this analysis was largely based on 2 factors: (1) the GBD study currently provides estimates of the burden of only these specific neurological disorders, and (2) these neurological disorders are considered by the GBD study as conditions for which neurologists play a particular important role in care and diagnosis (as opposed to, for example, low back pain, for which the role of a neurologist is less dominant). Second, Gooch et al^1^ based their analysis on previously published articles only, while the GBD study analyses also included administrative data and modeling, thus allowing estimates for US states with no epidemiological data on a particular neurological disorder. Differences in the methodologies may explain differences in the estimates of stroke prevalence (7.8 million in the GBD study vs 6.8 million in the Gooch et al study^1^), AD and other dementias (2.9 million vs 5.3 million), epilepsy (1.4 million vs 2.8 million), TBI (2.1 million vs 1.4 million), and SCI (2.2 million vs 0.3 million). The large difference in SCI cases may reflect a weakness in GBD methods, which rely on sparse data on the association between the incidence of a cause of injury (eg, falls and road injury) and the nature of the injury (eg, fall or SCI), which should be examined in future rounds of GBD. Our GBD estimates of PD (0.6 million), migraine (69 million), MS (0.4 million), and MND (38 000) are close to those reported by Gooch et al (0.6 million, 72 million, 0.4 million, and 18 000-30 000, respectively).^1^ Third, the Gooch et al^1^ study was based on articles published prior to 2017, with many of them referring to the data collected 5 to 10 years ago; thus, ongoing aging of the population and population growth may partly explain the greater prevalence of selected neurological disorders in our study.

### Limitations

General limitations of the GBD study discussed elsewhere^3^ fully apply to this report and may account for differences between these data and those collected by other governmental or disease-specific organizations. Specifically, for this report, we were not able to provide burden estimates for all neurological disorders combined, or burden estimates by age and sex, because these estimates will be the subjects of separate reports. In addition, some very prevalent neurological disorders (eg, restless leg syndrome and peripheral neuropathy are not currently estimated by GBD) and some common disorders (eg, low back and neck pain) are not regarded as neurological disorders in this article, although they are at least partly within the realm of neurology. Inclusion of these currently unaccounted disorders would increase the estimates of the burden of neurological disorders. Other limitations specific to this report include (1) difficulty in determining death from dementia because coding practices have changed by orders of magnitude over the last 30 years; (2) lack of data to quantify headaches in the US; and (3) accuracy of medical claims and hospitalization data and potential inaccuracies in the adjustments of administrative data to those reported in epidemiological studies. Because GBD study estimates are updated annually, the current limitations can be addressed, and with an increasing amount of data being added year to year, a more accurate picture of the burden of neurological disorders in the US can be made.

## Conclusions

In summary, this report showed that, while there were reductions in the rates of most burden metrics of stroke, Alzheimer and other dementias, TBI, SCI, meningitis, and encephalitis, there was a large and increasing number of people affected by various neurological disorders in the US, with a significant variation of the burden of and trends in particular neurological disorders across the US states. The reasons for geographic variations among different US states need to be explored further. Health care professionals and policy makers at the national and state levels can use the information reported in this article to advance their health care planning and resource allocation, including research funding, to prevent and reduce the morbidity and mortality of neurological disorders.

## Notes

Supplement. **eFigure.** Age-standardized incidence, prevalence, mortality, and DALY rates per 100,000 persons for neurological disorders in the US states in 2017. eAppendix. **eTable 1.** Age-adjusted incidence, prevalence, mortality, and disability-adjusted life years (DALY) rates per 100,000 persons for stroke by US states in 1990 and 2017, and the percentage change between 1990 and 2017 **eTable 2.** Age-adjusted incidence, prevalence, mortality, and disability-adjusted life years (DALY) rates per 100,000 persons for Alzheimer’s disease and other dementias by US states in 1990 and 2017, and the percentage change between 1990 and 2017 **eTable 3.** Age-adjusted incidence, prevalence, mortality, and disability-adjusted life years (DALY) rates per 100,000 for Parkinson’s disease by US states in 1990 and 2017, and the percentage change between 1990 and 2017 **eTable 4.** Age-adjusted incidence, prevalence, mortality, and disability-adjusted life years (DALY) rates per 100,000 for epilepsy by US states in 1990 and 2017, and the percentage change between 1990 and 2017 **eTable 5.** Age-adjusted incidence, prevalence, mortality, and disability-adjusted life years (DALY) rates per 100,000 for multiple sclerosis by US states in 1990 and 2017, and the percentage change between 1990 and 2017 **eTable 6.** Age-adjusted incidence, prevalence, mortality and disability-adjusted life years (DALY) rates per 100,000 for motor neuron disease by US states in 1990 and 2017, and the percentage change between 1990 and 2017 **eTable 7.** Age-adjusted incidence, prevalence, mortality, and disability-adjusted life years (DALY) rates per 100,000 for migraine by US states in 1990 and 2017, and the percentage change between 1990 and 2017 **eTable 8.** Age-adjusted incidence, prevalence, mortality, and disability-adjusted life years (DALY) rates per 100,000 for tension-type headache by US states in 1990 and 2017, and the percentage change between 1990 and 2017 **eTable 9.** Age-adjusted incidence, prevalence, mortality, and disability-adjusted life years (DALY) rates per 100,000 for traumatic brain injury by US states in 1990 and 2017, and the percentage change between 1990 and 2017 **eTable 10.** Age-adjusted incidence, prevalence, mortality, and disability-adjusted life years (DALY) rates per 100,000 for spinal cord injuries by US states in 1990 and 2017, and the percentage change between 1990 and 2017 **eTable 11.** Age-adjusted incidence, prevalence, mortality, and disability-adjusted life years (DALY) rates per 100,000 for brain and nervous system cancer by US states in 1990 and 2017, and the percentage change between 1990 and 2017 **eTable 12.** Age-adjusted incidence, prevalence, mortality, and disability-adjusted life years (DALY) rates per 100,000 for meningitis by US states in 1990 and 2017, and the percentage change between 1990 and 2017 **eTable 13.** Age-adjusted incidence, prevalence, mortality, and disability-adjusted life years (DALY) rates per 100,000 for encephalitis by US states in 1990 and 2017, and the percentage change between 1990 and 2017
