# Supplementary material for: Burden of Neurological Disorders Across the US From 1990-2017: A Global Burden of Disease Study
Source: JAMA Neurol. 2020 Nov 2;78(2):1–12. doi: 10.1001/jamaneurol.2020.4152 (PMC7607495; doi:10.1001/jamaneurol.2020.4152)
Supplement: Supplement. — eFigure. Age-standardized incidence, prevalence, mortality, and DALY rates per 100,000 persons for neurological disorders in the US states in 2017. eAppendix. eTable 1. Age-adjusted incidence, prevalence, mortality, and disability-adjusted life years (DALY) rates per 100,000 persons for stroke by US states in 1990 and 2017, and the percentage change between 1990 and 2017 eTable 2. Age-adjusted incidence, prevalence, mortality, and disability-adjusted life years (DALY) rates per 100,000 persons for Alzheimer’s disease and other dementias by US states in 1990 and 2017, and the percentage change between 1990 and 2017 eTable 3. Age-adjusted incidence, prevalence, mortality, and disability-adjusted life years (DALY) rates per 100,000 for Parkinson’s disease by US states in 1990 and 2017, and the percentage change between 1990 and 2017 eTable 4. Age-adjusted incidence, prevalence, mortality, and disability-adjusted life years (DALY) rates per 100,000 for epilepsy by US states in 1990 and 2017, and the percentage change between 1990 and 2017 eTable 5. Age-adjusted incidence, prevalence, mortality, and disability-adjusted life years (DALY) rates per 100,000 for multiple sclerosis by US states in 1990 and 2017, and the percentage change between 1990 and 2017 eTable 6. Age-adjusted incidence, prevalence, mortality and disability-adjusted life years (DALY) rates per 100,000 for motor neuron disease by US states in 1990 and 2017, and the percentage change between 1990 and 2017 eTable 7. Age-adjusted incidence, prevalence, mortality, and disability-adjusted life years (DALY) rates per 100,000 for migraine by US states in 1990 and 2017, and the percentage change between 1990 and 2017 eTable 8. Age-adjusted incidence, prevalence, mortality, and disability-adjusted life years (DALY) rates per 100,000 for tension-type headache by US states in 1990 and 2017, and the percentage change between 1990 and 2017 eTable 9. Age-adjusted incidence, prevalence, mortality, and disability-adjuste [file jamaneurol-e204152-s001.pdf]

Supplemental Online Content

GBD 2017 US Neurological Disorders Collaborators. Burden of neurological disorders across the US from 1990-2017: a Global Burden of Disease study. *JAMA Neurol*. Published online November 2, 2020. doi:10.1001/jamaneurol.2020.4152

eFigure. Age-standardized incidence, prevalence, mortality, and DALY rates per 100,000 persons for neurological disorders in the US states in 2017.
eAppendix.
eTable 1. Age-adjusted incidence, prevalence, mortality, and disability-adjusted life years (DALY) rates per 100,000 persons for stroke by US states in 1990 and 2017, and the percentage change between 1990 and 2017
eTable 2. Age-adjusted incidence, prevalence, mortality, and disability-adjusted life years (DALY) rates per 100,000 persons for Alzheimer's disease and other dementias by US states in 1990 and 2017, and the percentage change between 1990 and 2017
eTable 3. Age-adjusted incidence, prevalence, mortality, and disability-adjusted life years (DALY) rates per 100,000 for Parkinson's disease by US states in 1990 and 2017, and the percentage change between 1990 and 2017
eTable 4. Age-adjusted incidence, prevalence, mortality, and disability-adjusted life years (DALY) rates per 100,000 for epilepsy by US states in 1990 and 2017, and the percentage change between 1990 and 2017
eTable 5. Age-adjusted incidence, prevalence, mortality, and disability-adjusted life years (DALY) rates per 100,000 for multiple sclerosis by US states in 1990 and 2017, and the percentage change between 1990 and 2017
eTable 6. Age-adjusted incidence, prevalence, mortality and disability-adjusted life years (DALY) rates per 100,000 for motor neuron disease by US states in 1990 and 2017, and the percentage change between 1990 and 2017
eTable 7. Age-adjusted incidence, prevalence, mortality, and disability-adjusted life years (DALY) rates per 100,000 for migraine by US states in 1990 and 2017, and the percentage change between 1990 and 2017
eTable 8. Age-adjusted incidence, prevalence, mortality, and disability-adjusted life years (DALY) rates per 100,000 for tension-type headache by US states in 1990 and 2017, and the percentage change between 1990 and 2017
eTable 9. Age-adjusted incidence, prevalence, mortality, and disability-adjusted life years (DALY) rates per 100,000 for traumatic brain injury by US states in 1990 and 2017, and the percentage change between 1990 and 2017
eTable 10. Age-adjusted incidence, prevalence, mortality, and disability-adjusted life years (DALY) rates per 100,000 for spinal cord injuries by US states in 1990 and 2017, and the percentage change between 1990 and 2017
eTable 11. Age-adjusted incidence, prevalence, mortality, and disability-adjusted life years (DALY) rates per 100,000 for brain and nervous system cancer by US states in 1990 and 2017, and the percentage change between 1990 and 2017
eTable 12. Age-adjusted incidence, prevalence, mortality, and disability-adjusted life years (DALY) rates per 100,000 for meningitis by US states in 1990 and 2017, and the percentage change between 1990 and 2017
eTable 13. Age-adjusted incidence, prevalence, mortality, and disability-adjusted life years (DALY) rates per 100,000 for encephalitis by US states in 1990 and 2017, and the percentage change between 1990 and 2017

This supplemental material has been provided by the authors to give readers additional information about their work.

© 2020 GBD 2017 US Neurological Disorders Collaborators. *JAMA Neurology.*

eFigure. Age-standardized incidence, prevalence, mortality, and DALY rates per 100,000 persons for neurological disorders in the US states in 2017

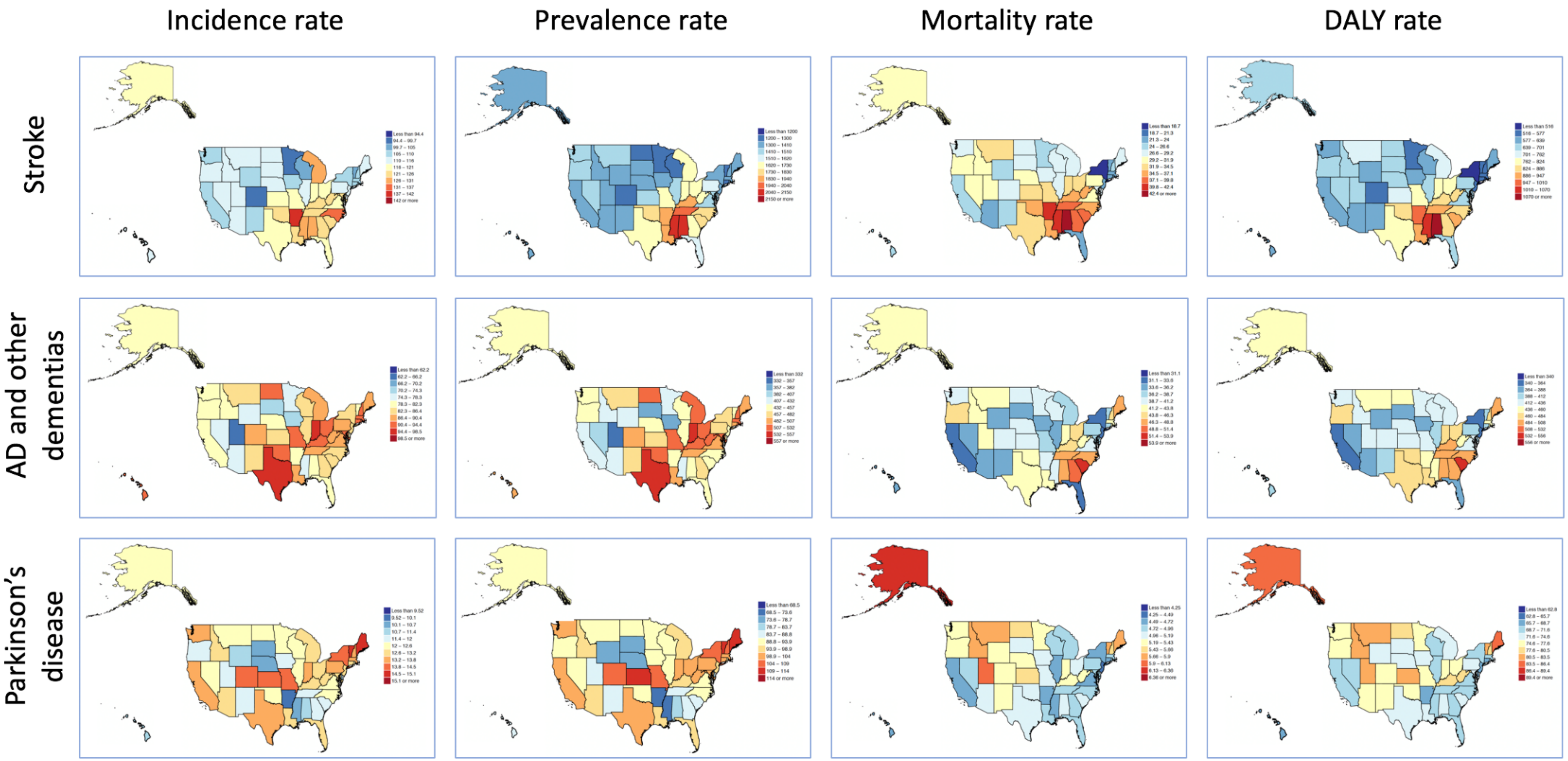

© 2020 GBD 2017 US Neurological Disorders Collaborators. *JAMA Neurology*.

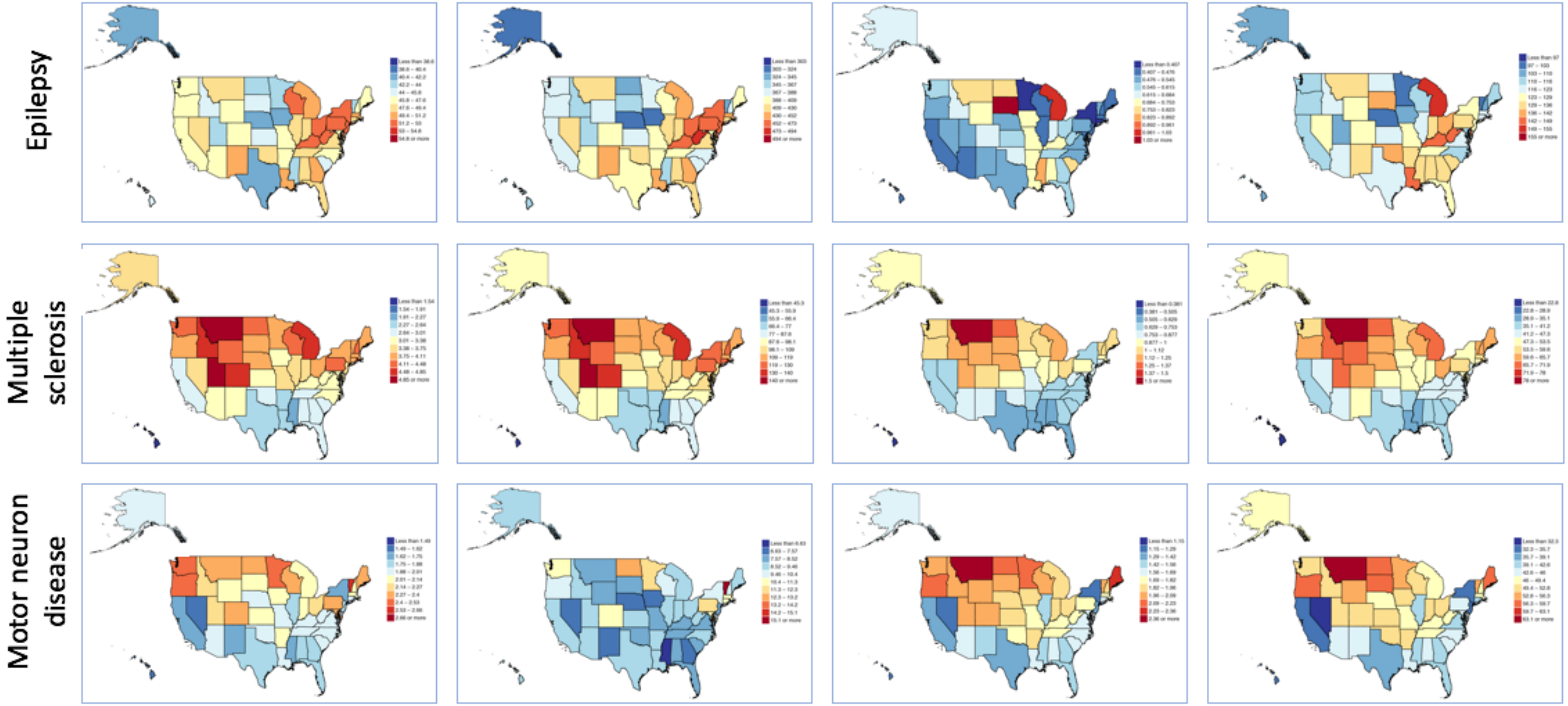

© 2020 GBD 2017 US Neurological Disorders Collaborators. *JAMA Neurology*.

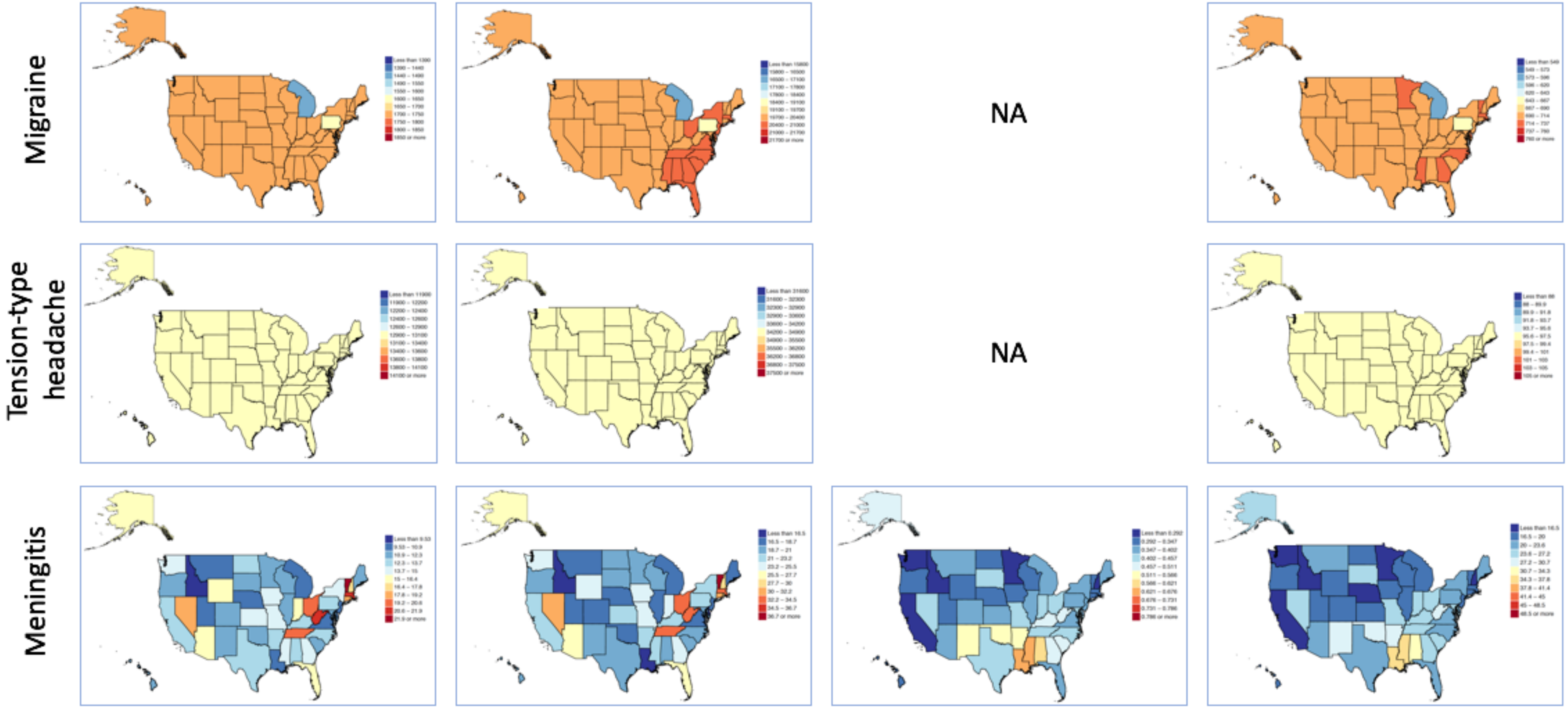

© 2020 GBD 2017 US Neurological Disorders Collaborators. *JAMA Neurology*.

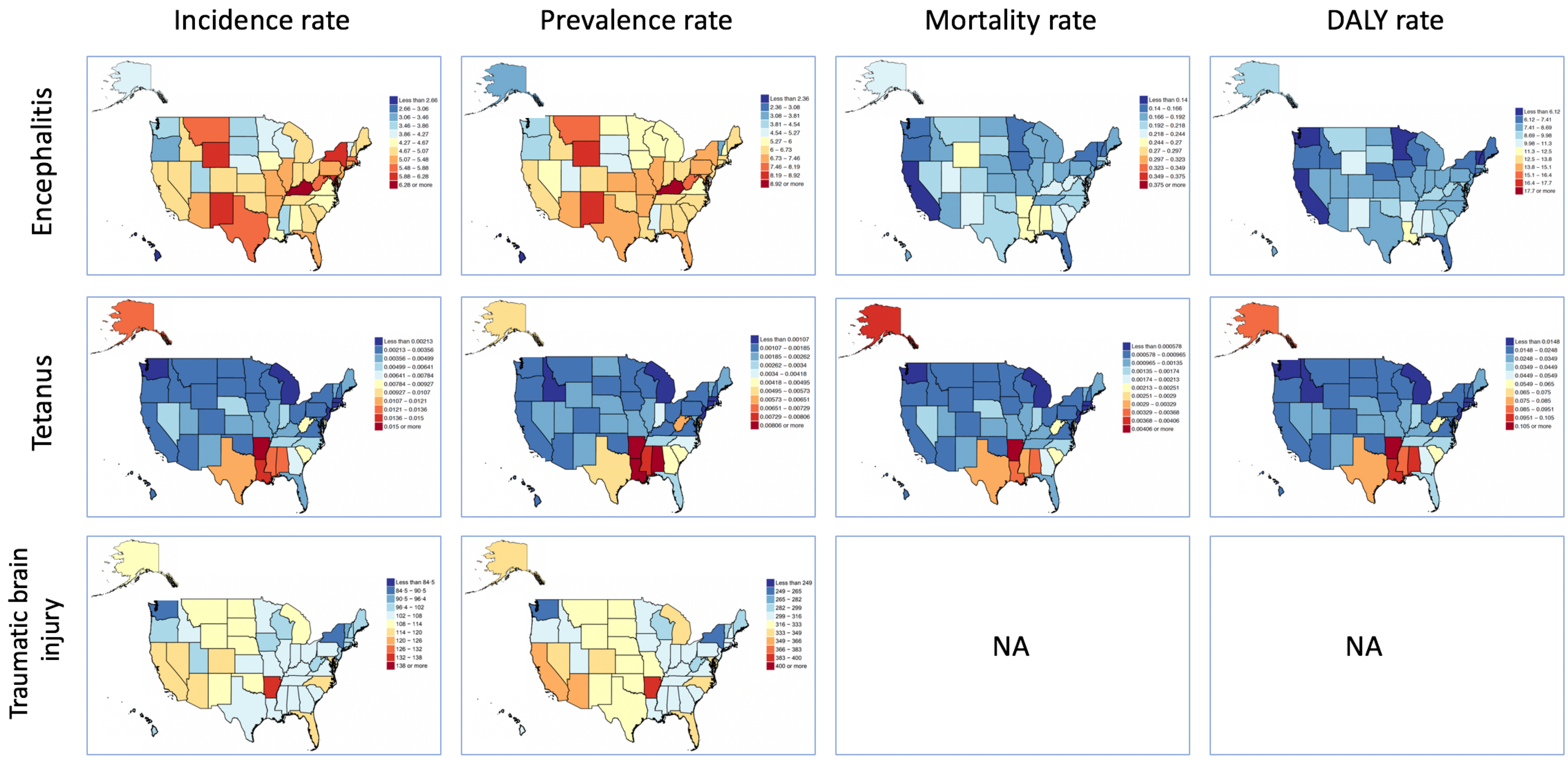

© 2020 GBD 2017 US Neurological Disorders Collaborators. *JAMA Neurology*.

eAppendix.

Stroke and Alzheimer's disease and other dementias

Almost all age-standardized metrics for stroke and Alzheimer's disease and other dementias were highest in the Southeastern states and lowest in the Northern and Western states (Figure 1). For stroke, the highest incidence rate in 2017 was in Arkansas (137 per 100,000 [95% UI 126–149]), and the lowest in Colorado (99 per 100,000 [92–107]). Similarly, the highest (2,133 [2,015–2,268]) and lowest (1,211 [1,135–1,295]) prevalence was in Alabama and Wisconsin, respectively; the highest mortality and DALY rates were in Alabama (43 [39–48] and 1,082 [961–1,208], respectively) and the lowest in New York (18 [16–20] and 504 [438–566], respectively). For Alzheimer's disease and other dementias, the highest incidence and prevalence rates were in Indiana (96 [88–105] and 547 [498–604]) and the lowest in Utah (65 [58–72] and 342 [309–377]); the highest mortality rates were in in South Carolina (53 [49–58]) and the lowest in New York (32 [29–35]). Finally, the highest DALY rate was in in South Carolina (545 [497–598]) and the lowest in California (351 [319–382]).

Parkinson's disease

Age-standardized incidence and prevalence rates of PD were highest in Eastern and Central states, particularly in Vermont (15 [95% UI 13–17] and 112 [97–126]) and lowest in Arkansas (10 [9–11] and 71 [61–80]); while the highest mortality and DALY rates were in Northern states, especially in Alaska (6 [4-7] and 86 [66-95]) and the lowest were in New Jersey and Hawaii (range 4–5 [range 3–5] and 66 [range 51–76]).

Idiopathic epilepsy

The largest burden of idiopathic epilepsy was observed in Eastern states. The age-standardized incidence rate of epilepsy was highest in Delaware (52 [95% UI 15–83]), and the lowest in Texas (41 [11–65]); the highest and lowest prevalence rates were in West Virginia (484 [142–758]) and Iowa (313 [94–481]), respectively. The highest and lowest

© 2020 GBD 2017 US Neurological Disorders Collaborators. *JAMA Neurology.*

mortality rates were in South Dakota (1 [1–1]) and Connecticut (0 [0–0]); the highest DALY rate was in Michigan (152 [70–288]) and the lowest in Vermont (101 [38–209]).

Multiple sclerosis

The burden of MS was particularly high in Northern states (except for Alaska). Age-standardized incidence and mortality rates of multiple sclerosis ranged from 1–5 to 0–2 per 100,000 people, respectively, across all US states; prevalence rates were highest in Utah (147 [95% UI 138–157]) and lowest in Hawaii (39 [36–42]); the highest DALY rate was in Montana (82 [53–102]) and the lowest in Hawaii (19 [14–27]).

Motor neuron disease

Very low age-standardized incidence, prevalence, and mortality rates were observed across all US states for MND (2–3, 6–15, and 1–2, respectively); DALY rates were highest in Montana (63 [95% UI 54–72]) and lowest in Nevada (32 [28–36]).

Migraine and tension-type headache

Age-standardized incidence, prevalence, and DALY rates of migraine and TTH were evenly distributed across all US states, except for a few Eastern states where the rates for migraine were noticeably greater. The highest incidence, prevalence, and DALY rates of migraine were observed in the District of Columbia (1,756 [95% UI 1,613–1,899], 20,644 [19,098–22,227], and 726 [462–1,051]) and the lowest in Michigan (1,488 [1,337–1,646], 16,862 [15,259–18,507], and 584 [367–860], respectively). The District of Columbia had the highest incidence and prevalence of TTH (13,021 [11,617–14,431] and 34,665 [31,361–38,129]), and Maryland had the lowest (12,975 [11,526–14,352] and 34,412 [31,086–38,237]). While DALY rates of TTH were also highest in the District of Columbia (97 [55–154]), they were lowest in West Virginia (96 [54–154]).

© 2020 GBD 2017 US Neurological Disorders Collaborators. *JAMA Neurology*.

Traumatic brain and spinal cord injuries

TBI (and to a lesser extent, SCI) age-standardized incidence and prevalence rates were relatively evenly distributed across US states. Arkansas had the highest incidence and prevalence rates of TBI (354 [95% UI 297–421] and 618 [589–647]). The lowest incidence of TBI was in New York (245 [205–294]), and the lowest prevalence of TBI was in Washington (417 [398–437]). Alaska demonstrated the highest incidence and prevalence of SCI (26 [21–33] and 672 [620–728], respectively). Hawaii showed the lowest incidence of SCI (19 [15–24]), and Tennessee had the lowest prevalence of SCI (492 [453–532]).

Brain and other nervous system cancers

There was relatively low variation in the age-standardized incidence and prevalence of brain and other nervous system cancers across the US states, with somewhat higher rates in North Dakota (10 [95% UI 8–12] and 49 [37–61], respectively) and lower rates in Nevada and Mississippi (6 [5–8]) and 25 [19–31], respectively). Mortality and DALY rates from brain and other nervous system cancers were relatively high in North and South Dakota (5 [4–6] and 163 [120–192], respectively) and relatively low in New Mexico (3 [3–4] for mortality) and New York (105 [91–141] for DALYs).

Meningitis, encephalitis, and tetanus

Most of the age-standardized burden from meningitis, encephalitis, and tetanus was located in the Eastern and Southern states (except incidence and prevalence rates from encephalitis, which were also high in Northern and South-central states). The highest incidence and prevalence rates of meningitis were observed in Vermont (22 [95% UI 20–26] and 37 [33–43]), and the lowest in Idaho (9 [8–10] and 16 [14–18], respectively). The District of Columbia showed the highest mortality and DALY rates of meningitis (1 [1–1] and 51 [38–64], respectively), while Minnesota showed the lowest mortality rate (0 [0–0]) and Idaho

© 2020 GBD 2017 US Neurological Disorders Collaborators. *JAMA Neurology.*

showed the lowest DALY rate (14 [12–22]) from this disease. The highest incidence and prevalence rates of encephalitis were observed in Kentucky (6 [6–7] and 9 [5–15], respectively). While mortality from encephalitis was virtually zero across all US states, the highest DALY rate of this disease was observed in the District of Columbia (18 [10–23]) and the lowest in Washington (6 [5–9]).

Between-state variations in the burden of neurological conditions

As shown in the Figures and Supplementary Tables 1-13, age-standardized rates of neurological disorders varied widely between the US states. Between-state incidence rates (per 100,000 people) varied from a 1.4-fold difference for SCI to 3.2-fold and 7.1-fold for MS and tetanus, respectively. For prevalence, the lowest between-state variations were for TTH (1.2-fold difference) and the highest for encephalitis and tetanus (3.8-fold and 7.5-fold, respectively). Mortality rates varied from a 1.5-fold difference for PD to 3.9-fold and 7.0-fold for MS and tetanus, respectively. Lowest variations in DALY rates were observed for TTH (1.2-fold difference), and highest variations for MS and tetanus (3.4-fold and 7.1-fold differences, respectively).

© 2020 GBD 2017 US Neurological Disorders Collaborators. *JAMA Neurology.*

eTable 1. Age-adjusted incidence, prevalence, mortality, and disability-adjusted life years (DALY) rates per 100,000 persons for stroke by US states in 1990 and 2017, and the percentage change between 1990 and 2017

Location	Incidence rates (95% UI)				Prevalence (95% UI)				Mortality rates (95% UI)				DALY rates (95% UI)			
	1990	2017	Change	Rank	1990	2017	Change	Rank	1990	2017	Change	Rank	1990	2017	Change	Rank
Alabama	150 (140,161)	129 (119,140)	-13.8	40	1839 (1728,1947)	2133 (2015,2268)	16	50	53 (51,55)	43 (39,48)	-18.5	47	1191 (1105,1276)	1082 (961,1208)	-9.2	49
Alaska	143 (133,155)	116 (106,127)	-19.2	16	1442 (1353,1538)	1351 (1268,1445)	-6.3	11	54 (51,56)	31 (29,34)	-41.9	3	958 (890,1030)	682 (610,755)	-28.9	12
Arizona	131 (121,141)	113 (105,122)	-13.4	43	1423 (1332,1516)	1377 (1299,1459)	-3.2	21	37 (35,38)	23 (21,26)	-36.3	14	773 (709,834)	580 (511,648)	-25	22
Arkansas	155 (144,166)	137 (126,149)	-11.1	48	1781 (1679,1882)	1917 (1812,2037)	7.6	43	53 (51,55)	40 (36,44)	-24.9	39	1154 (1071,1236)	983 (870,1097)	-14.8	42
California	132 (122,142)	110 (102,121)	-16.4	28	1437 (1352,1533)	1352 (1282,1433)	-5.9	12	41 (40,43)	24 (22,27)	-41.1	6	870 (803,936)	593 (525,660)	-31.9	5
Colorado	123 (113,133)	99 (92,107)	-19.2	14	1309 (1223,1393)	1220 (1157,1287)	-6.8	7	38 (36,39)	27 (24,30)	-28.5	30	745 (682,804)	564 (496,631)	-24.2	27
Connecticut	133 (123,144)	107 (98,117)	-19.7	11	1414 (1323,1507)	1226 (1159,1299)	-13.3	1	36 (34,37)	22 (20,25)	-37.8	10	758 (693,817)	519 (450,583)	-31.5	6
Delaware	135 (125,146)	112 (102,122)	-17.5	21	1504 (1413,1593)	1510 (1422,1611)	0.4	32	41 (39,43)	27 (25,30)	-32.9	25	866 (795,934)	649 (575,720)	-25.1	21
District of Columbia	155 (145,167)	109 (99,119)	-30	1	1747 (1646,1862)	1662 (1553,1783)	-4.9	16	54 (51,59)	25 (22,29)	-54.2	1	1345 (1240,1503)	697 (607,800)	-48.2	1
Florida	134 (124,145)	117 (107,127)	-12.8	44	1565 (1473,1663)	1568 (1483,1660)	0.2	30	36 (35,38)	24 (21,27)	-34.4	19	852 (785,918)	644 (573,723)	-24.4	26
Georgia	156 (145,167)	124 (114,135)	-20.7	7	1713 (1613,1818)	1720 (1623,1830)	0.4	31	56 (54,58)	37 (34,41)	-33.1	24	1204 (1130,1281)	881 (781,985)	-26.8	17
Hawaii	142 (132,153)	112 (103,123)	-21.2	6	1444 (1356,1534)	1339 (1268,1414)	-7.3	6	43 (41,44)	26 (23,28)	-40.4	8	909 (843,974)	631 (561,705)	-30.6	7
Idaho	137 (127,148)	113 (103,122)	-17.8	18	1458 (1371,1554)	1372 (1289,1463)	-5.9	13	43 (41,45)	30 (27,33)	-30.2	27	835 (772,903)	640 (565,709)	-23.4	28
Illinois	142 (132,153)	114 (105,125)	-19.7	12	1565 (1474,1662)	1492 (1403,1585)	-4.7	17	42 (40,43)	29 (26,32)	-31.3	26	930 (859,997)	673 (589,755)	-27.6	15
Indiana	143 (133,154)	118 (108,128)	-17.6	20	1587 (1494,1693)	1654 (1566,1754)	4.2	40	45 (44,47)	33 (30,37)	-26.8	34	945 (875,1015)	782 (695,878)	-17.2	38
Iowa	125 (116,136)	108 (101,117)	-13.6	41	1361 (1277,1445)	1319 (1248,1401)	-3.1	22	37 (35,38)	27 (24,30)	-26.8	35	744 (683,806)	607 (531,681)	-18.4	37
Kansas	135 (125,145)	116 (107,127)	-13.6	42	1476 (1391,1572)	1509 (1428,1596)	2.3	36	38 (37,40)	33 (29,36)	-14.5	49	805 (734,869)	727 (636,809)	-9.7	48
Kentucky	141 (131,153)	123 (113,134)	-12.7	45	1691 (1586,1796)	1916 (1806,2048)	13.3	48	46 (44,48)	37 (33,41)	-20.2	44	989 (917,1062)	876 (771,977)	-11.5	44
Louisiana	145 (136,156)	121 (111,131)	-16.8	24	1718 (1621,1829)	1917 (1807,2043)	11.6	47	49 (47,51)	35 (32,40)	-27.1	32	1127 (1045,1202)	917 (815,1026)	-18.6	36
Maine	131 (122,142)	110 (101,121)	-16	31	1395 (1307,1482)	1334 (1259,1416)	-4.4	19	38 (36,39)	28 (26,31)	-24.9	38	769 (705,832)	622 (548,695)	-19.1	35
Maryland	139 (129,150)	125 (116,135)	-10.4	49	1536 (1445,1631)	1511 (1430,1593)	-1.6	26	42 (40,44)	28 (25,31)	-34.3	21	906 (831,976)	672 (593,755)	-25.8	20
Massachusetts	131 (122,142)	106 (97,116)	-19.2	15	1437 (1352,1527)	1372 (1287,1463)	-4.5	18	36 (34,37)	23 (21,26)	-34.1	22	759 (693,820)	556 (487,625)	-26.7	18
Michigan	145 (135,157)	127 (116,138)	-12.6	46	1686 (1578,1787)	1717 (1626,1823)	1.8	35	41 (40,43)	29 (26,32)	-29.3	28	929 (856,1001)	720 (629,814)	-22.5	29
Minnesota	125 (116,136)	99 (91,109)	-20.6	9	1331 (1247,1422)	1233 (1159,1309)	-7.4	5	41 (40,43)	25 (23,28)	-38.3	9	786 (725,850)	558 (491,627)	-29.1	11
Mississippi	150 (140,161)	128 (118,139)	-14.7	36	1809 (1706,1915)	2097 (1984,2223)	15.9	49	51 (49,53)	41 (37,47)	-19.2	45	1198 (1112,1284)	1067 (947,1191)	-11	45
Missouri	138 (128,148)	118 (108,128)	-14.3	38	1565 (1473,1660)	1636 (1538,1744)	4.6	41	42 (40,43)	33 (30,36)	-20.8	43	907 (834,978)	774 (682,867)	-14.6	43
Montana	133 (124,145)	114 (105,124)	-14.6	37	1432 (1346,1516)	1449 (1366,1539)	1.2	34	42 (40,44)	33 (30,36)	-21.9	42	830 (762,895)	702 (626,788)	-15.3	41
Nebraska	135 (126,146)	113 (104,123)	-16.4	30	1419 (1329,1505)	1326 (1253,1404)	-6.6	9	40 (38,42)	29 (27,33)	-26.4	37	820 (757,887)	646 (571,723)	-21.2	30

© 2020 GBD 2017 US Neurological Disorders Collaborators. *JAMA Neurology*.

Nevada	133 (123,144)	113 (104,122)	-15.5	33	1462 (1373,1551)	1455 (1371,1551)	-0.5	28	45 (43,48)	30 (27,33)	-34.5	18	919 (851,991)	693 (616,771)	-24.6	25
New Hampshire	135 (125,145)	107 (98,117)	-20.6	8	1485 (1393,1572)	1384 (1303,1476)	-6.8	8	40 (38,41)	25 (23,27)	-37.6	11	799 (731,867)	565 (494,640)	-29.3	10
New Jersey	132 (122,143)	112 (102,123)	-15.3	35	1469 (1381,1561)	1373 (1302,1459)	-6.5	10	37 (36,39)	22 (19,24)	-41.8	4	839 (774,903)	558 (488,629)	-33.4	3
New Mexico	132 (122,142)	109 (100,119)	-17.6	19	1413 (1324,1504)	1390 (1309,1470)	-1.6	25	39 (37,40)	25 (23,27)	-35.9	15	798 (733,859)	599 (527,669)	-24.9	23
New York	126 (116,137)	105 (98,115)	-16.5	27	1457 (1365,1554)	1376 (1295,1464)	-5.6	14	36 (35,37)	18 (16,20)	-50	2	826 (758,887)	504 (438,566)	-38.9	2
North Carolina	158 (147,170)	134 (123,145)	-15.3	34	1755 (1653,1865)	1799 (1702,1904)	2.5	38	53 (51,55)	35 (32,39)	-33.6	23	1129 (1050,1207)	828 (736,925)	-26.6	19
North Dakota	135 (125,146)	113 (104,122)	-16.4	29	1363 (1282,1445)	1293 (1220,1373)	-5.2	15	40 (38,42)	29 (27,31)	-27.8	31	796 (732,860)	662 (595,730)	-16.9	39
Ohio	136 (126,147)	117 (108,127)	-13.8	39	1573 (1480,1669)	1674 (1579,1775)	6.4	42	40 (39,42)	34 (30,37)	-16.3	48	883 (811,950)	789 (697,878)	-10.6	46
Oklahoma	139 (129,150)	123 (113,133)	-12.1	47	1597 (1505,1695)	1737 (1643,1846)	8.8	44	45 (43,46)	36 (32,40)	-19.1	46	963 (889,1032)	867 (770,966)	-10	47
Oregon	142 (132,153)	111 (102,122)	-21.7	3	1484 (1396,1572)	1458 (1370,1550)	-1.7	24	46 (44,48)	30 (27,33)	-34.7	17	880 (814,945)	662 (580,745)	-24.8	24
Pennsylvania	138 (128,149)	114 (105,124)	-17.2	23	1545 (1452,1644)	1558 (1471,1653)	0.8	33	39 (38,41)	30 (27,33)	-24.8	40	869 (799,938)	697 (618,782)	-19.8	33
Rhode Island	131 (121,142)	106 (97,116)	-19.4	13	1442 (1351,1535)	1330 (1255,1414)	-7.8	4	36 (34,37)	21 (19,24)	-40.5	7	773 (705,840)	536 (469,600)	-30.6	8
South Carolina	161 (150,173)	125 (115,136)	-22.4	2	1748 (1644,1850)	1794 (1692,1901)	2.6	39	60 (58,63)	40 (36,44)	-34.4	20	1317 (1234,1401)	939 (835,1047)	-28.7	13
South Dakota	132 (122,142)	111 (102,121)	-15.9	32	1401 (1312,1489)	1369 (1291,1453)	-2.3	23	38 (36,39)	29 (27,32)	-22.6	41	789 (726,852)	666 (591,740)	-15.6	40
Tennessee	152 (142,163)	123 (114,135)	-18.9	17	1785 (1683,1894)	1946 (1835,2071)	9	45	53 (51,55)	38 (34,42)	-28.8	29	1134 (1048,1214)	911 (810,1022)	-19.7	34
Texas	141 (130,152)	116 (107,127)	-17.4	22	1587 (1493,1682)	1625 (1536,1722)	2.4	37	45 (43,46)	32 (29,36)	-27	33	960 (888,1028)	767 (682,851)	-20.1	31
Utah	135 (124,146)	112 (103,122)	-16.7	25	1405 (1320,1495)	1387 (1312,1470)	-1.3	27	43 (41,45)	32 (29,35)	-26.6	36	820 (756,881)	657 (588,729)	-19.9	32
Vermont	132 (122,143)	104 (96,115)	-21.2	5	1393 (1309,1480)	1284 (1212,1365)	-7.8	3	39 (38,41)	23 (21,25)	-41.7	5	778 (713,844)	519 (458,578)	-33.2	4
Virginia	148 (138,159)	116 (107,127)	-21.6	4	1568 (1471,1669)	1505 (1424,1595)	-4	20	49 (47,50)	31 (27,34)	-37.1	12	994 (920,1065)	699 (615,779)	-29.7	9
Washington	131 (121,142)	109 (102,118)	-16.5	26	1425 (1341,1512)	1423 (1348,1508)	-0.1	29	43 (41,45)	27 (24,30)	-36.7	13	831 (765,894)	606 (530,685)	-27.1	16
West Virginia	135 (125,145)	121 (111,133)	-10.3	50	1572 (1480,1668)	1722 (1623,1835)	9.6	46	41 (39,42)	36 (32,40)	-11.8	50	907 (837,979)	841 (749,934)	-7.3	50
Wisconsin	128 (118,139)	103 (95,111)	-19.8	10	1338 (1252,1430)	1211 (1135,1295)	-9.5	2	41 (40,43)	27 (24,30)	-35.8	16	817 (754,875)	584 (514,655)	-28.6	14

© 2020 GBD 2017 US Neurological Disorders Collaborators. *JAMA Neurology*.

eTable 2. Age-adjusted incidence, prevalence, mortality, and disability-adjusted life years (DALY) rates per 100,000 persons for Alzheimer's disease and other dementias by US states in 1990 and 2017, and the percentage change between 1990 and 2017

Location	Incidence rates (95% UI)				Prevalence (95% UI)				Mortality rates (95% UI)				DALY rates (95% UI)			
	1990	2017	Change	Rank	1990	2017	Change	Rank	1990	2017	Change	Rank	1990	2017	Change	Rank
Alabama	96 (84,110)	80 (73,88)	-17.1	9	534 (456,618)	446 (407,491)	-16.5	17	35 (34,36)	47 (43,51)	33.7	47	419 (393,445)	504 (457,550)	20.4	49
Alaska	95 (82,110)	81 (74,89)	-14.6	19	530 (449,617)	445 (402,491)	-16.1	19	50 (48,51)	43 (40,46)	-13.5	1	514 (485,544)	450 (420,481)	-12.3	1
Arizona	95 (82,109)	78 (71,86)	-17.3	8	527 (449,612)	429 (393,470)	-18.5	9	35 (34,36)	35 (32,38)	0.7	9	404 (379,433)	385 (351,423)	-4.8	13
Arkansas	96 (83,113)	80 (72,88)	-17	11	532 (448,624)	435 (396,479)	-18.4	11	35 (34,36)	41 (38,45)	17.8	35	415 (389,444)	452 (409,495)	8.8	37
California	93 (80,108)	79 (72,87)	-15	18	512 (437,606)	423 (384,465)	-17.4	13	34 (33,34)	32 (30,35)	-3.7	4	392 (367,420)	351 (319,382)	-10.5	4
Colorado	99 (86,112)	89 (81,98)	-9.2	35	555 (480,638)	495 (450,544)	-10.9	30	37 (35,37)	41 (37,45)	11.8	26	423 (396,451)	428 (383,469)	1.3	24
Connecticut	99 (87,112)	91 (83,100)	-7.4	42	554 (481,633)	506 (460,556)	-8.7	39	36 (35,37)	37 (33,40)	2	14	419 (391,447)	401 (361,443)	-4.4	14
Delaware	97 (84,112)	81 (74,89)	-16.5	12	539 (460,630)	439 (399,480)	-18.5	10	38 (37,39)	38 (36,41)	1.5	13	433 (406,462)	407 (378,438)	-5.9	10
District of Columbia	102 (90,116)	96 (87,105)	-6.4	47	577 (499,660)	538 (488,596)	-6.7	45	39 (38,40)	39 (37,42)	0.1	7	482 (454,512)	452 (413,493)	-6.3	7
Florida	96 (83,110)	81 (73,89)	-15.9	14	536 (460,625)	444 (405,488)	-17.2	14	32 (31,33)	32 (30,35)	1.3	11	389 (363,417)	370 (336,404)	-4.9	12
Georgia	97 (85,110)	83 (76,92)	-14.1	21	537 (461,618)	461 (419,508)	-14.2	26	37 (36,37)	49 (45,54)	34.8	49	427 (402,455)	505 (461,553)	18.2	47
Hawaii	98 (86,110)	91 (82,99)	-7.4	43	556 (483,632)	506 (460,556)	-9	38	36 (35,36)	36 (33,39)	1.2	10	415 (389,444)	401 (366,440)	-3.4	16
Idaho	95 (83,108)	78 (71,86)	-17.3	7	528 (453,609)	429 (391,471)	-18.7	7	36 (35,37)	42 (39,44)	15.6	29	412 (387,441)	434 (401,466)	5.5	29
Illinois	97 (84,111)	83 (76,92)	-13.7	23	537 (461,622)	454 (414,496)	-15.5	22	35 (34,36)	36 (33,39)	2.7	15	414 (388,441)	391 (357,430)	-5.5	11
Indiana	101 (89,115)	96 (88,105)	-5.2	49	571 (494,653)	547 (498,604)	-4.1	49	37 (36,38)	43 (40,47)	16.4	32	436 (409,464)	475 (433,524)	8.8	38
Iowa	93 (79,109)	73 (66,81)	-21.4	3	508 (424,599)	380 (345,417)	-25.3	3	33 (32,34)	35 (32,38)	4.9	18	388 (362,417)	377 (340,413)	-2.9	17
Kansas	97 (85,111)	85 (78,94)	-12.2	28	542 (468,625)	469 (428,518)	-13.4	28	35 (34,36)	39 (36,43)	12	27	409 (381,438)	426 (387,468)	4.2	27
Kentucky	100 (88,114)	92 (83,100)	-8.7	37	563 (485,645)	518 (473,568)	-7.9	41	36 (35,37)	46 (42,50)	26.5	44	433 (407,461)	495 (449,541)	14.2	46
Louisiana	99 (87,113)	89 (80,98)	-10.9	30	557 (482,638)	498 (450,549)	-10.5	32	36 (35,37)	42 (39,46)	18.7	37	428 (402,455)	472 (431,519)	10.3	41
Maine	99 (87,113)	90 (82,99)	-8.9	36	556 (479,641)	505 (459,555)	-9.2	35	37 (36,38)	47 (43,50)	26.9	45	427 (400,455)	485 (444,525)	13.4	44
Maryland	97 (86,111)	91 (83,100)	-6.5	46	543 (472,619)	509 (465,560)	-6.1	47	37 (36,38)	39 (35,42)	4.1	17	428 (403,455)	420 (383,462)	-1.9	18
Massachusetts	93 (82,106)	92 (83,101)	-1.3	50	518 (449,593)	512 (467,564)	-1.2	50	35 (33,36)	41 (38,45)	20.1	42	405 (380,431)	447 (407,493)	10.5	43
Michigan	100 (88,113)	90 (82,99)	-9.7	33	561 (487,644)	508 (463,557)	-9.4	34	36 (34,37)	38 (34,41)	5.4	19	422 (395,450)	419 (378,459)	-0.9	20
Minnesota	96 (83,110)	78 (71,85)	-18.7	5	533 (456,619)	426 (389,466)	-20	5	36 (35,37)	41 (38,45)	14.3	28	415 (390,443)	422 (383,462)	1.6	25
Mississippi	94 (80,110)	77 (69,85)	-18.5	6	515 (430,605)	416 (377,461)	-19.1	6	34 (33,35)	41 (37,45)	19.7	39	407 (380,435)	444 (404,488)	9.2	40
Missouri	100 (88,113)	92 (84,101)	-7.4	41	559 (484,642)	517 (471,566)	-7.5	42	36 (35,37)	39 (35,42)	7.2	20	426 (399,454)	430 (392,472)	0.8	22
Montana	97 (85,110)	86 (79,95)	-11.1	29	544 (473,621)	474 (432,523)	-12.8	29	37 (36,38)	44 (41,47)	17.3	34	424 (398,451)	460 (422,499)	8.4	33
Nebraska	95 (82,110)	79 (72,87)	-17	10	528 (451,616)	433 (394,477)	-18.1	12	36 (34,36)	39 (36,43)	11.1	24	411 (386,440)	423 (387,459)	2.8	26

© 2020 GBD 2017 US Neurological Disorders Collaborators. *JAMA Neurology.*

Nevada	93 (79,112)	75 (68,83)	-19.8	4	510 (420,613)	397 (362,440)	-22.2	4	39 (38,40)	36 (33,38)	-9.3	2	426 (401,455)	375 (347,405)	-12	2
New Hampshire	101 (89,115)	94 (86,103)	-6.7	45	572 (497,655)	530 (481,581)	-7.5	43	38 (37,39)	44 (41,48)	16.2	31	441 (414,470)	467 (429,506)	5.9	30
New Jersey	95 (82,111)	83 (75,91)	-13.4	25	529 (452,616)	447 (407,492)	-15.4	23	34 (33,35)	32 (29,36)	-5.2	3	403 (378,430)	359 (325,397)	-10.9	3
New Mexico	96 (85,110)	84 (77,93)	-12.4	27	540 (466,621)	464 (423,509)	-14.1	27	37 (36,38)	36 (33,38)	-2.7	5	422 (397,450)	396 (364,429)	-6	9
New York	98 (86,111)	85 (78,93)	-13	26	547 (472,626)	468 (429,517)	-14.4	25	32 (31,32)	32 (29,35)	0.5	8	387 (362,415)	360 (324,399)	-7	6
North Carolina	98 (86,113)	82 (75,91)	-15.9	13	551 (471,643)	449 (409,493)	-18.6	8	37 (36,38)	48 (44,52)	29	46	434 (408,463)	493 (450,538)	13.5	45
North Dakota	99 (87,113)	91 (83,100)	-8.5	38	564 (487,648)	512 (464,564)	-9.2	37	37 (36,38)	40 (37,43)	8.6	21	422 (394,452)	439 (408,474)	4.2	28
Ohio	100 (88,113)	93 (85,102)	-6.8	44	563 (489,644)	527 (481,577)	-6.3	46	36 (35,37)	44 (40,48)	20.2	43	432 (406,459)	477 (431,520)	10.3	42
Oklahoma	100 (88,113)	92 (84,101)	-8	39	560 (482,638)	518 (473,570)	-7.4	44	36 (35,37)	42 (38,46)	15.7	30	426 (401,454)	464 (421,509)	8.7	36
Oregon	96 (84,111)	81 (74,89)	-15.4	15	537 (461,620)	451 (412,496)	-16	20	37 (36,38)	45 (41,49)	20	41	424 (397,452)	461 (418,506)	8.9	39
Pennsylvania	100 (88,114)	87 (79,95)	-13.6	24	564 (494,645)	478 (433,522)	-15.3	24	36 (35,37)	36 (33,39)	1.3	12	426 (399,455)	399 (364,436)	-6.2	8
Rhode Island	102 (90,115)	94 (86,103)	-7.6	40	575 (499,655)	527 (478,578)	-8.4	40	35 (34,36)	41 (38,45)	17.8	36	421 (395,452)	447 (411,485)	6.2	31
South Carolina	97 (85,111)	83 (76,91)	-14.3	20	541 (463,625)	457 (414,500)	-15.7	21	36 (35,37)	53 (49,58)	46.4	50	430 (405,458)	545 (497,598)	26.8	50
South Dakota	92 (78,108)	70 (63,78)	-23.3	2	502 (417,596)	371 (333,411)	-26	2	34 (33,35)	35 (32,37)	2.7	16	385 (360,413)	370 (343,398)	-4.1	15
Tennessee	97 (85,112)	88 (80,97)	-9.5	34	541 (461,627)	491 (446,541)	-9.2	36	36 (35,36)	48 (43,52)	34.5	48	423 (399,451)	504 (458,552)	19	48
Texas	100 (88,114)	95 (87,104)	-5.3	48	566 (489,645)	539 (491,591)	-4.7	48	37 (36,38)	44 (40,47)	19	38	433 (407,461)	471 (430,513)	8.7	35
Utah	88 (75,105)	65 (58,72)	-26.8	1	480 (398,573)	342 (309,377)	-28.8	1	34 (33,35)	41 (38,44)	20	40	388 (365,415)	422 (388,455)	8.5	34
Vermont	95 (82,111)	81 (74,89)	-15.1	16	529 (449,617)	438 (399,482)	-17.1	15	38 (36,39)	37 (35,40)	-0.9	6	425 (399,454)	395 (364,427)	-7	5
Virginia	99 (87,112)	89 (81,98)	-10.2	32	556 (483,637)	496 (452,547)	-10.7	31	37 (36,38)	41 (37,44)	8.9	23	434 (407,461)	435 (395,478)	0.3	21
Washington	95 (82,110)	81 (73,89)	-15	17	528 (447,620)	438 (400,481)	-16.9	16	37 (36,37)	41 (37,44)	11.2	25	417 (391,445)	420 (383,460)	0.8	23
West Virginia	101 (88,114)	90 (82,99)	-10.5	31	566 (492,648)	511 (465,562)	-9.7	33	35 (34,36)	41 (38,45)	17.3	33	424 (396,454)	456 (417,499)	7.6	32
Wisconsin	97 (84,111)	83 (76,91)	-14.1	22	539 (462,624)	452 (410,496)	-16.2	18	36 (36,37)	40 (36,44)	8.8	22	420 (395,448)	413 (373,454)	-1.6	19

© 2020 GBD 2017 US Neurological Disorders Collaborators. *JAMA Neurology*.

eTable 3. Age-adjusted incidence, prevalence, mortality, and disability-adjusted life years (DALY) rates per 100,000 for Parkinson's disease by US states in 1990 and 2017, and the percentage change between 1990 and 2017

Location	Incidence rates (95% UI)				Prevalence (95% UI)				Mortality rates (95% UI)				DALY rates (95% UI)			
	1990	2017	Change	Rank	1990	2017	Change	Rank	1990	2017	Change	Rank	1990	2017	Change	Rank
Alabama	10 (8,12)	11 (9,12)	8.7	5	78 (61,97)	80 (69,91)	3.7	6	4 (3,4)	5 (4,6)	42.6	46	56 (51,69)	74 (64,83)	32.1	44
Alaska	11 (9,13)	12 (10,14)	15	12	83 (67,101)	89 (78,100)	8	8	6 (6,6)	6 (4,7)	3.4	1	80 (75,87)	86 (66,95)	6.7	1
Arizona	11 (9,13)	13 (11,15)	19.9	28	84 (68,104)	96 (84,108)	14.5	29	4 (4,4)	5 (4,6)	35	29	59 (55,66)	75 (58,85)	27.1	32
Arkansas	10 (8,12)	10 (9,11)	2.2	3	74 (58,94)	71 (61,80)	-4.9	2	3 (3,4)	5 (4,5)	33.1	22	54 (49,65)	68 (60,76)	24.8	25
California	11 (9,13)	13 (12,15)	24.7	40	86 (69,105)	102 (88,114)	19	40	4 (4,4)	5 (4,5)	26.8	11	58 (53,66)	70 (56,78)	20	11
Colorado	11 (9,13)	14 (12,16)	27.2	44	88 (72,107)	107 (93,120)	21.8	45	4 (4,4)	5 (4,6)	36.5	34	61 (56,68)	77 (61,87)	27.2	33
Connecticut	11 (9,13)	13 (11,15)	24.2	39	85 (68,103)	100 (87,113)	18.2	38	4 (3,4)	5 (4,5)	25.8	8	58 (52,68)	69 (58,77)	19	8
Delaware	10 (8,12)	11 (9,13)	9.8	6	78 (62,97)	80 (70,91)	3.6	5	4 (4,5)	5 (4,5)	20.7	4	61 (56,73)	69 (60,75)	12.9	3
District of Columbia	10 (8,13)	13 (11,14)	22.4	32	81 (65,101)	95 (83,108)	17.2	32	4 (4,5)	5 (4,5)	14.1	2	68 (63,81)	73 (62,83)	7.6	2
Florida	11 (9,13)	13 (11,14)	19.3	23	84 (68,104)	96 (84,108)	14.1	25	4 (3,4)	5 (4,5)	32.6	20	57 (52,64)	71 (57,79)	24.3	23
Georgia	10 (8,12)	12 (10,13)	16.4	15	78 (62,97)	86 (74,97)	10.2	16	4 (3,5)	5 (4,5)	35.5	30	57 (52,71)	70 (61,79)	23.7	17
Hawaii	10 (8,12)	11 (10,13)	18.9	22	75 (62,92)	85 (73,95)	13	23	4 (4,4)	5 (3,5)	19.1	3	57 (52,64)	66 (51,76)	16.4	5
Idaho	11 (9,13)	13 (11,15)	18.3	18	85 (69,104)	97 (84,110)	14.3	27	4 (4,5)	6 (4,7)	39.1	39	62 (57,69)	81 (62,91)	29.7	38
Illinois	10 (9,13)	12 (11,14)	19.3	24	83 (68,101)	93 (82,105)	12.6	22	4 (3,4)	5 (4,5)	28.3	13	58 (52,69)	69 (59,77)	19.7	10
Indiana	11 (9,13)	14 (12,15)	26.6	42	85 (69,105)	102 (89,115)	20.3	42	4 (4,5)	5 (4,6)	38.9	37	59 (54,70)	77 (65,87)	31.4	42
Iowa	10 (8,13)	12 (10,14)	15.4	13	82 (66,101)	89 (77,100)	8.8	12	4 (3,4)	5 (4,6)	40.5	41	56 (51,64)	73 (60,82)	30.3	40
Kansas	11 (9,14)	14 (12,16)	28.1	46	89 (72,110)	110 (96,123)	23.1	46	4 (4,4)	6 (4,6)	49.6	50	59 (54,67)	83 (66,93)	39.2	50
Kentucky	11 (9,13)	13 (11,15)	22.1	31	84 (68,102)	98 (86,111)	17.4	33	4 (4,5)	5 (4,6)	41.9	45	59 (53,70)	78 (66,87)	33	46
Louisiana	11 (9,13)	13 (11,15)	19.6	26	84 (69,103)	96 (84,108)	14.5	28	4 (3,5)	5 (4,5)	32.9	21	59 (54,72)	74 (65,82)	25	26
Maine	11 (9,13)	15 (12,17)	31.9	49	88 (71,107)	111 (97,126)	25.5	49	4 (4,5)	6 (4,7)	44.6	48	63 (57,70)	84 (68,95)	34.2	47
Maryland	11 (9,13)	13 (11,15)	23.6	36	84 (69,103)	100 (87,112)	18.1	37	4 (4,5)	5 (4,5)	26.4	10	61 (55,73)	72 (61,81)	19.4	9
Massachusetts	11 (9,13)	13 (12,15)	25.3	41	85 (69,105)	102 (89,115)	20	41	4 (3,5)	5 (4,5)	32.3	19	58 (53,69)	71 (61,80)	24.1	19
Michigan	11 (9,13)	13 (11,15)	22.9	34	84 (68,103)	99 (85,112)	17.8	35	4 (3,5)	5 (4,6)	34	26	58 (53,70)	73 (63,82)	26.2	30
Minnesota	10 (8,13)	12 (11,14)	19.7	27	83 (67,101)	92 (80,104)	11.4	18	4 (4,4)	5 (4,6)	36.1	33	60 (55,68)	75 (59,84)	25.2	27
Mississippi	10 (8,13)	10 (9,12)	-2.2	1	80 (63,100)	72 (61,85)	-9.3	1	4 (3,4)	5 (4,5)	33.8	25	56 (51,68)	69 (61,77)	22.8	16
Missouri	11 (9,13)	14 (12,16)	29.9	47	87 (70,106)	107 (93,120)	23.6	47	4 (4,4)	5 (4,6)	39	38	59 (54,69)	77 (65,86)	30.9	41
Montana	10 (8,13)	12 (11,14)	18.7	20	82 (66,102)	92 (80,104)	11.5	20	4 (4,5)	6 (5,7)	48.5	49	60 (55,68)	82 (68,92)	37.3	49
Nebraska	10 (8,12)	11 (9,12)	8.3	4	77 (62,97)	77 (67,88)	0.1	4	4 (4,4)	5 (4,6)	41.6	43	58 (53,67)	74 (60,83)	27.8	35

© 2020 GBD 2017 US Neurological Disorders Collaborators. *JAMA Neurology.*

Nevada	10 (8,13)	12 (11,14)	18.1	17	81 (65,99)	90 (78,102)	11	17	4 (4,5)	5 (4,6)	21.9	6	61 (56,72)	72 (61,80)	17.3	6
New Hampshire	11 (9,13)	14 (12,16)	27.2	43	86 (70,107)	104 (91,118)	20.9	43	4 (4,5)	6 (4,6)	33.6	24	63 (58,72)	79 (65,88)	24.2	21
New Jersey	11 (9,13)	13 (11,15)	22.7	33	84 (68,103)	98 (86,111)	16.8	30	4 (3,5)	4 (4,5)	21.5	5	58 (53,71)	67 (57,75)	15.4	4
New Mexico	10 (8,13)	12 (10,13)	13.2	8	81 (65,100)	88 (76,98)	8.5	11	4 (4,4)	5 (4,6)	30.9	14	62 (57,68)	77 (60,86)	24.8	24
New York	10 (9,13)	14 (12,16)	33.6	50	83 (68,101)	107 (93,121)	29.5	50	4 (3,4)	5 (4,5)	28.1	12	57 (52,68)	69 (57,78)	22.2	14
North Carolina	10 (8,12)	12 (10,14)	18.8	21	80 (65,98)	90 (78,101)	12.2	21	4 (3,4)	5 (4,5)	33.2	23	57 (52,69)	71 (61,80)	24.2	20
North Dakota	11 (9,13)	12 (11,14)	15.6	14	85 (68,104)	93 (80,105)	9.8	15	4 (4,5)	5 (4,6)	36	32	61 (56,69)	79 (66,87)	29.8	39
Ohio	11 (9,13)	13 (11,15)	23.2	35	84 (68,103)	99 (87,111)	18.6	39	4 (3,5)	5 (4,6)	40.2	40	58 (53,70)	76 (65,85)	32	43
Oklahoma	10 (8,13)	12 (10,14)	18.4	19	80 (64,99)	89 (78,101)	11.5	19	4 (3,4)	5 (4,6)	41.8	44	57 (51,66)	75 (65,84)	32.9	45
Oregon	10 (8,13)	12 (10,14)	13.9	10	81 (65,100)	89 (77,100)	9.2	13	4 (4,5)	5 (4,6)	32.3	18	60 (55,68)	75 (60,84)	24.1	18
Pennsylvania	11 (9,13)	13 (12,15)	23.6	37	86 (70,106)	101 (88,114)	17.1	31	4 (4,5)	5 (4,5)	34.1	27	58 (53,70)	73 (63,81)	25.7	29
Rhode Island	10 (8,13)	12 (11,14)	19.5	25	82 (67,100)	93 (81,105)	13.2	24	4 (4,4)	5 (4,5)	30.9	15	59 (54,68)	72 (61,81)	22.1	13
South Carolina	10 (8,13)	11 (10,13)	13	7	80 (64,98)	86 (76,97)	8.3	9	4 (3,4)	5 (4,6)	40.5	42	57 (52,69)	74 (63,83)	27.9	36
South Dakota	10 (8,12)	10 (9,12)	2.1	2	78 (62,98)	75 (65,85)	-4.4	3	4 (3,4)	5 (4,6)	38.4	36	56 (51,65)	72 (62,79)	27.4	34
Tennessee	10 (8,12)	12 (10,13)	14	11	79 (64,97)	86 (74,98)	8.4	10	4 (3,4)	5 (4,5)	34.7	28	56 (51,69)	71 (63,79)	25.3	28
Texas	11 (9,13)	14 (12,16)	27.7	45	85 (70,104)	104 (91,117)	21.8	44	4 (4,4)	5 (4,6)	35.6	31	59 (54,67)	75 (62,83)	27	31
Utah	10 (8,13)	12 (10,13)	13.3	9	81 (65,101)	88 (76,99)	7.7	7	4 (4,5)	6 (4,7)	36.7	35	65 (59,71)	83 (61,94)	28.1	37
Vermont	11 (9,14)	15 (13,17)	30.9	48	90 (73,110)	112 (97,126)	24	48	5 (4,5)	6 (4,6)	25.5	7	69 (63,76)	83 (65,92)	20.4	12
Virginia	10 (8,13)	13 (11,14)	21.1	29	82 (66,102)	94 (81,106)	14.2	26	4 (4,5)	5 (4,5)	26.4	9	58 (53,71)	69 (60,77)	18.6	7
Washington	11 (9,13)	13 (12,15)	23.6	38	86 (70,104)	102 (88,114)	18	36	4 (4,4)	5 (4,6)	31.4	16	61 (56,68)	75 (61,85)	24.2	22
West Virginia	11 (9,13)	13 (11,15)	21.3	30	85 (69,104)	100 (87,112)	17.7	34	4 (3,5)	5 (4,6)	43.7	47	58 (53,71)	79 (68,88)	35.1	48
Wisconsin	10 (8,13)	12 (10,14)	16.5	16	81 (66,100)	89 (78,101)	9.5	14	4 (4,5)	5 (4,6)	31.6	17	57 (53,68)	70 (59,79)	22.7	15

© 2020 GBD 2017 US Neurological Disorders Collaborators. *JAMA Neurology*.

eTable 4. Age-adjusted incidence, prevalence, mortality, and disability-adjusted life years (DALY) rates per 100,000 for epilepsy by US states in 1990 and 2017, and the percentage change between 1990 and 2017

Location	Incidence rates (95% UI)				Prevalence (95% UI)				Mortality rates (95% UI)				DALY rates (95% UI)			
	1990	2017	Change	Rank	1990	2017	Change	Rank	1990	2017	Change	Rank	1990	2017	Change	Rank
Alabama	40 (11,65)	48 (14,75)	21.5	38	342 (101,560)	413 (117,635)	20.8	42	1 (1,1)	1 (1,1)	9.4	36	121 (50,239)	136 (57,264)	12.1	43
Alaska	38 (11,61)	42 (13,65)	8.4	4	314 (87,494)	316 (101,488)	0.7	2	1 (1,1)	1 (1,1)	-2.8	18	112 (52,217)	105 (48,203)	-5.6	2
Arizona	39 (10,64)	47 (14,73)	21.3	37	329 (86,534)	395 (117,596)	19.9	37	0 (0,1)	0 (0,1)	-1.2	23	105 (40,212)	117 (40,240)	10.8	40
Arkansas	39 (12,64)	47 (13,73)	18.5	27	335 (100,533)	395 (111,608)	17.7	31	1 (1,1)	1 (1,1)	11.1	42	118 (50,218)	130 (53,258)	10.3	36
California	41 (10,67)	46 (12,73)	12.1	6	365 (93,581)	386 (99,602)	5.6	5	0 (0,1)	0 (0,1)	-5.1	13	116 (41,231)	113 (38,240)	-2	4
Colorado	40 (11,65)	48 (15,74)	18.8	29	347 (89,561)	397 (129,609)	14.5	22	1 (1,1)	1 (1,1)	2	29	117 (47,229)	124 (51,250)	5.8	22
Connecticut	42 (12,66)	49 (15,78)	18	25	364 (108,578)	426 (131,666)	16.9	27	0 (0,0)	0 (0,0)	-2.2	20	109 (38,229)	117 (38,249)	7.4	29
Delaware	44 (11,71)	52 (15,83)	19.3	31	409 (105,641)	483 (139,751)	17.9	32	1 (1,1)	1 (1,1)	-3.7	15	138 (51,264)	145 (53,287)	5.5	21
District of Columbia	39 (10,63)	41 (12,64)	6.6	3	325 (89,529)	314 (89,483)	-3.4	1	1 (1,1)	1 (1,1)	-38.4	1	138 (68,230)	105 (45,210)	-24	1
Florida	40 (11,65)	49 (14,76)	22.1	41	353 (101,575)	424 (122,652)	20.3	40	1 (1,1)	1 (1,1)	-12	5	120 (51,233)	129 (46,260)	6.8	25
Georgia	41 (10,65)	50 (13,77)	23.4	45	358 (88,570)	434 (117,661)	21.3	43	1 (1,1)	1 (0,1)	-15.6	3	123 (50,232)	132 (49,265)	6.8	26
Hawaii	37 (10,60)	46 (14,70)	23.5	46	303 (85,494)	363 (114,547)	20	38	0 (0,0)	0 (0,0)	-2.2	21	95 (36,186)	105 (38,216)	10.4	37
Idaho	39 (13,64)	44 (12,71)	12.7	8	333 (110,536)	362 (105,570)	8.7	8	1 (1,1)	1 (1,1)	0.1	26	113 (48,223)	117 (48,233)	3.3	14
Illinois	40 (12,64)	49 (16,76)	21	36	353 (102,556)	418 (133,646)	18.7	35	1 (1,1)	0 (0,1)	-13.2	4	117 (46,235)	122 (42,249)	4.3	18
Indiana	41 (10,66)	49 (13,78)	21.7	40	357 (89,574)	429 (111,670)	20.3	39	1 (1,1)	1 (1,1)	10.4	40	115 (46,227)	134 (51,264)	16.6	48
Iowa	37 (11,59)	41 (13,65)	11.2	5	298 (87,476)	313 (94,481)	5	4	1 (1,1)	1 (1,1)	9.9	38	103 (47,196)	105 (48,198)	2.2	10
Kansas	39 (10,64)	46 (13,72)	15.9	18	336 (85,551)	379 (107,584)	12.7	18	1 (0,1)	1 (1,1)	12.3	43	108 (41,222)	118 (47,237)	9.1	32
Kentucky	42 (13,68)	52 (14,82)	24.3	47	378 (118,600)	467 (127,726)	23.5	48	1 (1,1)	1 (1,1)	14.4	44	127 (50,247)	145 (57,280)	13.5	46
Louisiana	42 (12,67)	50 (16,80)	20.1	32	374 (108,591)	441 (145,692)	18.1	33	1 (1,1)	1 (1,1)	3.1	31	130 (58,233)	146 (61,279)	11.7	41
Maine	40 (11,64)	47 (12,75)	18.2	26	341 (95,549)	393 (106,614)	15.5	23	0 (0,0)	0 (0,1)	10	39	104 (37,210)	114 (39,237)	9.8	34
Maryland	41 (11,65)	47 (14,76)	13.9	12	361 (99,569)	398 (118,625)	10.3	12	0 (0,1)	0 (0,1)	-0.7	25	112 (39,224)	115 (39,231)	3	12
Massachusetts	42 (13,67)	50 (14,79)	17.3	22	380 (119,603)	431 (123,675)	13.3	20	0 (0,0)	0 (0,1)	2	30	114 (39,230)	120 (42,252)	5.3	20
Michigan	43 (11,68)	50 (13,79)	16.4	20	390 (98,617)	438 (117,676)	12.2	16	1 (1,1)	1 (1,1)	-1.5	22	148 (69,270)	152 (70,288)	2.8	11
Minnesota	42 (11,68)	44 (11,69)	4.1	2	366 (101,584)	369 (97,570)	0.8	3	0 (0,0)	0 (0,0)	3.4	32	106 (35,227)	102 (32,217)	-3.5	3
Mississippi	39 (12,62)	44 (11,69)	13.2	9	324 (101,506)	349 (90,538)	7.8	7	1 (1,1)	1 (1,1)	1	28	127 (61,224)	130 (59,240)	1.9	9
Missouri	40 (10,65)	47 (14,74)	19	30	343 (91,557)	397 (119,613)	15.7	24	1 (1,1)	1 (1,1)	8.6	34	118 (46,236)	127 (51,256)	7.4	28
Montana	41 (11,66)	49 (12,76)	20.2	33	354 (96,569)	415 (99,637)	17.4	29	1 (1,1)	1 (1,1)	21.3	47	121 (46,238)	134 (50,259)	10.3	35
Nebraska	37 (11,59)	42 (13,66)	13.6	10	295 (86,471)	323 (101,500)	9.7	9	0 (0,1)	1 (0,1)	6.9	33	95 (38,189)	103 (42,211)	8.1	30

© 2020 GBD 2017 US Neurological Disorders Collaborators. *JAMA Neurology*.

Nevada	42 (14,67)	49 (15,78)	17.5	24	371 (121,587)	430 (126,662)	15.8	25	1 (1,1)	0 (0,1)	-10.4	7	120 (47,245)	126 (42,260)	5.8	23
New Hampshire	39 (12,63)	47 (13,73)	18.7	28	330 (96,521)	384 (103,593)	16.3	26	0 (0,0)	0 (0,0)	9.8	37	99 (37,206)	108 (38,222)	9	31
New Jersey	42 (11,68)	52 (14,81)	22.2	42	386 (99,610)	461 (130,712)	19.2	36	0 (0,0)	0 (0,0)	-11.6	6	119 (41,251)	126 (40,272)	6.1	24
New Mexico	45 (11,74)	51 (14,80)	12.1	7	380 (102,611)	449 (128,692)	18.2	34	0 (0,1)	1 (0,1)	10.9	41	121 (45,240)	134 (49,275)	10.5	39
New York	44 (11,71)	52 (13,80)	17.3	23	384 (103,618)	472 (128,722)	22.7	46	0 (0,1)	0 (0,0)	-18	2	121 (42,246)	126 (41,274)	4.1	17
North Carolina	38 (11,61)	46 (13,72)	20.3	35	322 (87,515)	377 (107,584)	17	28	1 (1,1)	1 (0,1)	-9.2	8	110 (44,218)	115 (41,231)	4.5	19
North Dakota	38 (11,61)	44 (13,67)	15.7	17	309 (86,492)	342 (106,520)	10.7	14	1 (1,1)	1 (1,1)	31.6	50	105 (44,201)	119 (54,229)	12.4	44
Ohio	42 (12,67)	51 (14,80)	22.5	43	379 (109,612)	459 (121,711)	21.3	44	1 (0,1)	1 (1,1)	16.1	45	122 (44,248)	138 (50,279)	13.9	47
Oklahoma	40 (12,65)	49 (15,76)	22.8	44	351 (107,565)	427 (137,650)	21.6	45	1 (0,1)	1 (1,1)	21.6	48	114 (46,224)	136 (52,266)	20.2	49
Oregon	40 (11,64)	46 (14,72)	16.3	19	336 (92,536)	380 (121,591)	13.1	19	1 (1,1)	1 (0,1)	-5.3	12	110 (43,210)	114 (44,227)	3.8	16
Pennsylvania	42 (12,68)	51 (14,81)	21.6	39	382 (106,605)	459 (131,700)	20.3	41	1 (1,1)	1 (0,1)	-0.9	24	122 (45,239)	134 (47,285)	9.5	33
Rhode Island	41 (10,67)	49 (14,77)	20.3	34	361 (93,590)	424 (116,651)	17.6	30	0 (0,0)	0 (0,0)	-2.9	17	111 (38,229)	119 (40,254)	7.2	27
South Carolina	40 (12,66)	46 (14,72)	14.4	15	347 (100,558)	383 (123,597)	10.4	13	1 (1,1)	1 (1,1)	-4.3	14	130 (59,242)	131 (59,242)	0.6	7
South Dakota	39 (10,62)	45 (12,71)	14.4	14	331 (86,523)	365 (98,565)	10.1	10	1 (1,1)	1 (1,1)	24.8	49	126 (56,234)	139 (69,263)	10.4	38
Tennessee	39 (10,63)	48 (13,75)	24.3	48	333 (87,540)	408 (111,627)	22.7	47	1 (1,1)	1 (1,1)	-2.6	19	113 (45,224)	127 (48,254)	12.5	45
Texas	32 (9,53)	41 (11,65)	27.5	50	319 (93,521)	396 (106,610)	24	49	1 (1,1)	1 (0,1)	-3.4	16	108 (42,209)	121 (42,254)	11.9	42
Utah	38 (11,60)	43 (13,68)	13.8	11	313 (90,497)	347 (101,536)	10.9	15	0 (0,1)	1 (0,1)	9.2	35	101 (40,201)	105 (39,215)	3.5	15
Vermont	38 (12,60)	44 (12,69)	14.3	13	311 (92,490)	343 (97,529)	10.3	11	1 (0,1)	0 (0,1)	-5.6	11	101 (41,197)	101 (38,209)	0	6
Virginia	41 (10,66)	48 (13,76)	16.7	21	369 (93,587)	419 (120,647)	13.7	21	1 (1,1)	1 (0,1)	-8.1	9	119 (45,233)	122 (44,261)	3.2	13
Washington	40 (10,64)	46 (12,73)	15.6	16	338 (86,550)	380 (100,598)	12.4	17	1 (1,1)	1 (0,1)	-7.9	10	114 (46,220)	116 (44,239)	1.6	8
West Virginia	41 (12,67)	52 (15,83)	26.3	49	378 (116,612)	484 (142,758)	28.2	50	1 (0,1)	1 (1,1)	18	46	121 (46,232)	148 (55,311)	22.3	50
Wisconsin	51 (14,80)	52 (14,82)	3	1	376 (104,600)	399 (104,618)	6.2	6	0 (0,0)	0 (0,1)	0.6	27	116 (42,240)	116 (41,243)	-0.1	5

© 2020 GBD 2017 US Neurological Disorders Collaborators. *JAMA Neurology*.

eTable 5. Age-adjusted incidence, prevalence, mortality, and disability-adjusted life years (DALY) rates per 100,000 for multiple sclerosis by US states in 1990 and 2017, and the percentage change between 1990 and 2017

Location	Incidence rates (95% UI)				Prevalence (95% UI)				Mortality rates (95% UI)				DALY rates (95% UI)			
	1990	2017	Change	Rank	1990	2017	Change	Rank	1990	2017	Change	Rank	1990	2017	Change	Rank
Alabama	2 (2,3)	3 (2,3)	13.9	21	67 (60,75)	79 (73,86)	17.2	31	0 (0,1)	1 (1,1)	44.8	36	31 (24,43)	38 (31,50)	24.9	33
Alaska	3 (3,4)	3 (3,4)	1.7	1	93 (81,108)	96 (89,103)	2.2	1	1 (1,1)	1 (1,1)	30.6	16	45 (35,63)	51 (43,61)	13.1	8
Arizona	3 (2,3)	3 (3,3)	15.7	29	78 (71,87)	92 (85,98)	16.8	27	1 (1,1)	1 (1,1)	32.5	19	38 (32,44)	45 (36,53)	18.4	20
Arkansas	2 (2,2)	3 (2,3)	16.1	31	61 (55,69)	71 (67,77)	16.6	26	0 (0,1)	1 (1,1)	53	42	30 (24,40)	39 (32,48)	30	45
California	3 (2,3)	3 (3,3)	7.7	4	73 (66,81)	79 (74,85)	8.2	6	1 (1,1)	1 (0,1)	13.3	3	37 (31,43)	37 (30,45)	2.5	2
Colorado	4 (3,4)	5 (4,5)	19.3	44	111 (100,124)	133 (124,143)	19.9	44	1 (1,1)	1 (1,1)	39.6	26	52 (41,62)	63 (46,77)	22.1	28
Connecticut	3 (3,4)	4 (4,4)	13.8	20	103 (92,115)	120 (112,129)	17.1	30	1 (1,1)	1 (1,1)	20.8	6	46 (38,55)	52 (42,63)	13.6	9
Delaware	3 (3,3)	3 (3,4)	12.9	17	89 (80,100)	104 (97,112)	16.9	28	1 (1,1)	1 (1,1)	27.9	10	41 (33,50)	48 (40,57)	18.4	19
District of Columbia	3 (3,4)	3 (3,4)	2.8	2	90 (81,100)	92 (86,99)	2.9	2	1 (1,1)	1 (1,1)	0.3	1	50 (41,68)	48 (39,57)	-4	1
Florida	2 (2,3)	3 (3,3)	13.5	18	72 (65,80)	84 (78,90)	16.1	23	0 (0,1)	1 (0,1)	35.2	22	33 (27,40)	39 (32,46)	17.7	15
Georgia	2 (2,3)	3 (3,3)	15.4	28	68 (61,76)	81 (76,87)	19.6	40	0 (0,1)	1 (1,1)	31.9	18	32 (26,42)	39 (32,46)	19.7	24
Hawaii	1 (1,1)	1 (1,1)	2.8	3	37 (33,42)	39 (36,42)	4.6	3	0 (0,1)	0 (0,1)	20	5	17 (12,26)	19 (14,27)	9	4
Idaho	4 (3,4)	5 (4,5)	20.2	46	110 (100,122)	132 (123,141)	19.7	42	1 (1,1)	1 (1,2)	43.8	32	54 (42,64)	68 (48,82)	25.6	36
Illinois	3 (3,3)	3 (3,4)	16.6	36	86 (77,96)	102 (94,109)	18.3	36	1 (1,1)	1 (1,1)	30.9	17	43 (37,51)	50 (40,59)	16.5	13
Indiana	3 (3,3)	3 (3,4)	18.2	42	84 (76,94)	99 (92,105)	17.3	32	1 (1,1)	1 (1,1)	54.5	44	40 (33,48)	51 (41,61)	28.4	43
Iowa	3 (3,3)	3 (3,3)	10.8	9	84 (75,93)	91 (85,97)	8.1	5	1 (1,1)	1 (1,1)	51.8	41	41 (34,49)	50 (39,60)	22.6	30
Kansas	3 (3,3)	3 (3,3)	14.6	25	83 (75,92)	93 (87,100)	12.9	15	1 (1,1)	1 (1,1)	56	45	40 (34,48)	52 (39,62)	27.5	41
Kentucky	3 (2,3)	3 (3,3)	15.2	27	78 (70,88)	92 (86,99)	17.4	33	0 (0,1)	1 (1,1)	49	40	34 (27,45)	43 (36,53)	26.2	38
Louisiana	2 (2,2)	2 (2,2)	9.7	6	60 (53,67)	68 (63,73)	12.7	14	0 (0,1)	1 (0,1)	32.6	20	29 (22,40)	34 (28,45)	17.8	17
Maine	4 (3,4)	4 (4,4)	12.1	12	106 (95,117)	118 (111,126)	11.7	10	1 (1,1)	1 (1,1)	37.4	23	51 (42,59)	61 (46,73)	19.3	22
Maryland	3 (3,3)	3 (3,4)	12.5	14	86 (78,97)	99 (93,106)	14.8	20	1 (1,1)	1 (1,1)	28.3	11	42 (35,50)	48 (40,57)	16.1	11
Massachusetts	4 (3,4)	4 (4,4)	10.9	11	106 (95,118)	118 (110,126)	11.2	8	1 (1,1)	1 (1,1)	27.3	8	50 (41,58)	56 (44,67)	12.7	7
Michigan	4 (3,4)	5 (4,5)	23	48	106 (96,118)	131 (122,140)	23.8	49	1 (1,1)	1 (1,1)	47.6	38	52 (44,61)	66 (49,81)	26.7	40
Minnesota	4 (3,4)	4 (4,4)	9.1	5	102 (92,113)	109 (102,117)	6.8	4	1 (1,1)	1 (1,1)	38	24	49 (40,59)	57 (42,68)	14.9	10
Mississippi	2 (2,2)	2 (2,2)	12.8	15	49 (43,56)	56 (52,61)	14.4	18	0 (0,1)	1 (0,1)	42.4	30	25 (19,38)	31 (25,44)	24.3	31
Missouri	3 (3,3)	3 (3,4)	18.5	43	85 (77,95)	102 (95,109)	19.6	41	1 (1,1)	1 (1,1)	45.7	37	40 (33,48)	50 (40,59)	25.6	37
Montana	4 (4,5)	5 (5,5)	19.6	45	121 (109,134)	144 (135,153)	19.1	39	1 (1,1)	2 (1,2)	63	50	61 (46,72)	82 (53,102)	35	50
Nebraska	3 (3,4)	4 (4,4)	14.1	22	95 (86,106)	107 (100,113)	11.8	11	1 (1,1)	1 (1,2)	48.6	39	49 (40,57)	61 (43,74)	24.5	32

© 2020 GBD 2017 US Neurological Disorders Collaborators. *JAMA Neurology*.

Nevada	3 (3,3)	3 (3,4)	16.3	33	87 (78,97)	105 (97,112)	20.4	45	1 (0,1)	1 (1,1)	27.8	9	39 (31,48)	46 (38,55)	18.6	21
New Hampshire	4 (3,4)	4 (4,4)	12.4	13	108 (97,120)	123 (115,131)	13.8	16	1 (1,1)	1 (1,1)	30.4	15	51 (42,60)	60 (45,72)	17.7	16
New Jersey	3 (3,3)	3 (3,4)	10	7	91 (81,101)	102 (95,109)	12.4	12	1 (1,1)	1 (1,1)	17.8	4	43 (36,51)	47 (38,56)	8.8	3
New Mexico	3 (3,3)	3 (3,3)	17.3	39	79 (72,88)	94 (88,101)	19	38	1 (1,1)	1 (1,1)	42	29	39 (33,46)	50 (37,60)	26.5	39
New York	3 (3,4)	4 (4,4)	16	30	99 (89,110)	120 (111,129)	20.6	46	1 (1,1)	1 (1,1)	11.8	2	46 (37,55)	50 (40,61)	9.9	5
North Carolina	3 (2,3)	3 (3,3)	17.2	38	77 (69,86)	92 (85,99)	19.8	43	1 (0,1)	1 (1,1)	38.3	25	36 (28,45)	44 (36,51)	22.2	29
North Dakota	4 (3,4)	4 (4,4)	14.2	23	104 (94,115)	117 (109,125)	12.5	13	1 (1,1)	1 (1,2)	60.5	48	52 (42,61)	68 (49,83)	32.2	47
Ohio	3 (3,4)	4 (4,4)	23.8	50	94 (85,105)	116 (109,124)	23.6	48	1 (1,1)	1 (1,1)	58.4	47	45 (38,53)	60 (45,72)	33	49
Oklahoma	2 (2,3)	3 (3,3)	17.3	41	65 (59,73)	76 (71,81)	16.1	22	1 (0,1)	1 (1,1)	61.1	49	32 (26,41)	43 (34,51)	32.7	48
Oregon	4 (3,4)	4 (4,4)	14.9	26	102 (92,113)	117 (109,124)	14.2	17	1 (1,1)	1 (1,1)	40	27	51 (41,60)	60 (45,73)	19.4	23
Pennsylvania	3 (3,4)	4 (4,4)	21.1	47	101 (91,114)	124 (116,133)	22.5	47	1 (1,1)	1 (1,1)	44.8	35	46 (38,55)	58 (46,70)	24.9	34
Rhode Island	4 (4,4)	5 (4,5)	16.9	37	113 (102,126)	134 (126,144)	18.2	35	1 (1,1)	1 (1,1)	28.7	12	53 (44,63)	63 (48,76)	18	18
South Carolina	2 (2,3)	3 (3,3)	13.7	19	70 (63,79)	82 (77,88)	16.9	29	0 (0,1)	1 (1,1)	34.1	21	34 (27,44)	40 (33,48)	20.1	25
South Dakota	3 (3,4)	4 (4,4)	16.2	32	96 (87,107)	111 (104,118)	14.8	19	1 (1,1)	1 (1,2)	57.5	46	49 (39,57)	64 (46,78)	31.7	46
Tennessee	3 (2,3)	3 (3,3)	16.5	35	74 (67,83)	88 (83,94)	18.8	37	0 (0,1)	1 (1,1)	44.8	34	34 (27,44)	42 (35,51)	25.1	35
Texas	2 (2,2)	2 (2,3)	12.9	16	63 (56,71)	73 (68,79)	16.3	24	0 (0,1)	1 (0,1)	41.8	28	29 (23,38)	35 (29,41)	21.2	26
Utah	4 (4,4)	5 (5,5)	23.2	49	118 (107,131)	147 (138,157)	24.3	50	1 (1,1)	1 (1,2)	44.6	33	56 (42,67)	72 (49,88)	27.6	42
Vermont	4 (3,4)	4 (4,4)	10.7	8	106 (96,118)	118 (111,126)	11.6	9	1 (1,1)	1 (1,1)	29.1	14	52 (42,61)	60 (45,73)	16.2	12
Virginia	3 (3,3)	3 (3,4)	14.3	24	85 (77,95)	99 (93,106)	16.5	25	1 (0,1)	1 (1,1)	29	13	39 (32,47)	46 (37,54)	16.8	14
Washington	4 (3,4)	4 (4,5)	10.8	10	110 (100,123)	122 (114,130)	10.5	7	1 (1,1)	1 (1,1)	27.1	7	53 (44,63)	59 (46,70)	10.7	6
West Virginia	3 (2,3)	3 (3,3)	17.3	40	78 (69,87)	91 (85,98)	17.8	34	1 (0,1)	1 (1,1)	53.1	43	37 (30,46)	47 (38,57)	29.5	44
Wisconsin	4 (3,4)	4 (4,4)	16.4	34	103 (93,115)	119 (111,127)	15.2	21	1 (1,1)	1 (1,1)	43.3	31	49 (41,58)	60 (44,72)	21.4	27

© 2020 GBD 2017 US Neurological Disorders Collaborators. *JAMA Neurology*.

eTable 6. Age-adjusted incidence, prevalence, mortality and disability-adjusted life years (DALY) rates per 100,000 for motor neuron disease by US states in 1990 and 2017, and the percentage change between 1990 and 2017

Location	Incidence rates (95% UI)				Prevalence (95% UI)				Mortality rates (95% UI)				DALY rates (95% UI)			
	1990	2017	Change	Rank	1990	2017	Change	Rank	1990	2017	Change	Rank	1990	2017	Change	Rank
Alabama	1 (1,2)	2 (2,2)	22.5	47	7 (6,8)	8 (7,8)	6.4	40	1 (1,1)	2 (1,2)	62.1	46	31 (28,33)	45 (40,52)	48.5	49
Alaska	2 (2,2)	2 (2,2)	7	5	9 (8,10)	9 (8,10)	-1.5	19	1 (1,1)	2 (1,2)	32.7	12	37 (34,41)	46 (40,53)	23.7	21
Arizona	2 (2,2)	2 (2,2)	13.7	24	9 (8,10)	9 (8,10)	0.6	26	1 (1,1)	2 (1,2)	43.4	26	36 (33,38)	44 (39,51)	24.7	24
Arkansas	2 (2,2)	2 (2,2)	22.4	46	8 (7,9)	9 (8,9)	9.4	44	1 (1,1)	2 (2,2)	62.7	47	36 (33,39)	52 (45,60)	44.2	45
California	2 (2,2)	2 (2,2)	3	2	9 (8,10)	9 (8,9)	-5.1	13	1 (1,1)	1 (1,2)	17	3	36 (33,38)	35 (31,40)	-1.1	2
Colorado	2 (2,2)	2 (2,2)	16.1	32	11 (10,12)	11 (10,12)	2.4	34	1 (1,1)	2 (2,2)	41.2	22	42 (39,47)	51 (44,57)	20.1	15
Connecticut	2 (2,2)	2 (2,2)	7.2	7	11 (10,12)	11 (10,12)	-1.5	20	1 (1,1)	2 (1,2)	27.4	9	36 (33,39)	41 (36,47)	14.4	8
Delaware	2 (2,2)	2 (2,2)	10.5	13	10 (9,11)	10 (9,11)	5.3	38	1 (1,1)	2 (1,2)	27.3	8	38 (35,41)	45 (39,50)	18.4	14
District of Columbia	2 (2,2)	2 (2,2)	0.3	1	10 (9,11)	11 (10,12)	7.7	42	1 (1,1)	1 (1,1)	-1.2	1	45 (39,56)	36 (31,42)	-20.5	1
Florida	2 (2,2)	2 (2,2)	9.8	11	8 (7,9)	8 (8,9)	-2.8	17	1 (1,1)	1 (1,2)	37.4	18	35 (32,39)	40 (35,45)	12.9	7
Georgia	2 (1,2)	2 (2,2)	13.7	25	8 (7,8)	8 (7,8)	-0.9	23	1 (1,1)	1 (1,2)	43.3	25	32 (29,34)	39 (35,44)	24.4	23
Hawaii	1 (1,2)	2 (1,2)	12.4	19	9 (8,10)	9 (9,10)	3.5	36	1 (1,1)	1 (1,1)	35.3	15	27 (25,30)	34 (29,39)	23	20
Idaho	2 (2,2)	2 (2,2)	9.9	12	10 (9,11)	9 (8,9)	-12.5	5	1 (1,2)	2 (2,2)	38.1	19	44 (40,47)	52 (45,60)	20.1	16
Illinois	2 (2,2)	2 (2,2)	10.7	17	9 (8,10)	9 (8,10)	-0.3	24	1 (1,1)	2 (1,2)	36.4	16	35 (32,37)	41 (36,46)	17.7	12
Indiana	2 (2,2)	2 (2,2)	18.2	38	9 (8,10)	9 (8,10)	1.1	28	1 (1,1)	2 (2,2)	54.7	38	36 (33,39)	50 (44,56)	39.5	39
Iowa	2 (2,2)	2 (2,2)	13.3	21	9 (8,10)	7 (7,8)	-20.3	1	1 (1,1)	2 (2,2)	53.7	37	37 (34,40)	50 (43,56)	35.6	35
Kansas	2 (2,2)	2 (2,2)	19.1	40	9 (8,10)	9 (8,9)	-3	15	1 (1,1)	2 (2,2)	56.5	40	37 (34,40)	51 (44,57)	37.8	38
Kentucky	2 (2,2)	2 (2,2)	23.9	50	8 (7,9)	9 (8,9)	8.2	43	1 (1,1)	2 (2,2)	63.1	48	33 (31,37)	48 (42,55)	44.3	46
Louisiana	2 (2,2)	2 (2,2)	16.1	33	8 (7,9)	9 (9,10)	13.8	48	1 (1,1)	2 (1,2)	45.2	27	34 (31,37)	45 (40,52)	34.8	33
Maine	2 (2,2)	2 (2,2)	15.3	30	10 (9,11)	9 (9,10)	-5.2	12	1 (1,2)	2 (2,3)	53.3	36	41 (38,44)	59 (51,68)	43.1	43
Maryland	2 (2,2)	2 (2,2)	19.4	41	11 (9,12)	11 (10,12)	2.3	33	1 (1,1)	2 (1,2)	37.4	17	36 (34,39)	44 (39,50)	22.6	19
Massachusetts	2 (2,2)	2 (2,2)	10.7	16	11 (10,12)	11 (10,11)	-1.4	21	1 (1,1)	2 (2,2)	38.8	20	38 (35,40)	47 (41,53)	24.3	22
Michigan	2 (2,2)	2 (2,2)	15.1	29	9 (8,10)	9 (9,10)	1.7	30	1 (1,1)	2 (2,2)	47.5	32	37 (35,40)	49 (43,55)	31.1	32
Minnesota	2 (2,2)	3 (2,3)	16.2	34	12 (10,13)	11 (11,12)	-1.1	22	2 (1,2)	2 (2,2)	42.7	23	43 (40,47)	55 (48,63)	27.3	27
Mississippi	1 (1,2)	2 (2,2)	16.6	37	6 (6,7)	6 (6,7)	1	27	1 (1,1)	1 (1,2)	56.3	39	30 (27,33)	43 (37,49)	42.6	42
Missouri	2 (2,2)	2 (2,2)	19.6	42	8 (7,9)	8 (8,9)	0.6	25	1 (1,1)	2 (2,2)	53.2	35	35 (33,38)	47 (41,54)	35.2	34
Montana	2 (2,2)	2 (2,2)	15.8	31	9 (8,10)	8 (7,8)	-13.8	3	1 (1,2)	2 (2,3)	66.5	50	43 (39,48)	63 (54,72)	48.1	48
Nebraska	2 (2,2)	2 (2,2)	12.9	20	9 (8,10)	8 (7,8)	-14.8	2	1 (1,1)	2 (2,2)	45.6	29	38 (35,41)	48 (42,55)	28.6	28

© 2020 GBD 2017 US Neurological Disorders Collaborators. *JAMA Neurology*.

Nevada	1 (1,2)	2 (1,2)	4.6	3	7 (7,8)	7 (6,8)	-5.8	10	1 (1,1)	1 (1,1)	24.1	5	29 (27,32)	32 (28,36)	9.6	5
New Hampshire	2 (2,2)	2 (2,2)	10.6	14	11 (10,12)	10 (10,11)	-1.9	18	1 (1,2)	2 (2,2)	33.2	13	40 (37,43)	48 (42,54)	21.2	17
New Jersey	2 (2,2)	2 (2,2)	7.6	8	7 (7,8)	8 (7,9)	9.7	46	1 (1,1)	1 (1,1)	16.2	2	33 (31,36)	34 (30,39)	3.3	4
New Mexico	1 (1,2)	2 (2,2)	15	28	8 (7,8)	7 (7,8)	-3.6	14	1 (1,1)	2 (1,2)	51.1	33	32 (29,35)	43 (37,49)	36.6	37
New York	2 (1,2)	2 (2,2)	7.2	6	9 (8,10)	10 (9,11)	7.6	41	1 (1,1)	1 (1,1)	18.5	4	32 (30,35)	33 (29,37)	2.1	3
North Carolina	2 (2,2)	2 (2,2)	13.5	23	9 (8,10)	9 (8,10)	2.1	32	1 (1,1)	2 (1,2)	40.3	21	35 (33,38)	43 (38,49)	22.6	18
North Dakota	2 (2,2)	2 (2,3)	23.4	49	11 (10,12)	12 (11,14)	15.2	49	1 (1,1)	2 (2,2)	63.5	49	39 (36,43)	59 (53,67)	51	50
Ohio	2 (2,2)	2 (2,2)	18.7	39	9 (8,10)	9 (9,10)	1.9	31	1 (1,1)	2 (2,2)	58.7	42	37 (34,40)	52 (45,59)	40.5	40
Oklahoma	2 (2,2)	2 (2,2)	21.5	44	8 (7,8)	8 (7,8)	1.7	29	1 (1,1)	2 (2,2)	61.9	45	34 (31,37)	49 (43,56)	45.8	47
Oregon	2 (2,2)	2 (2,3)	14.5	27	10 (9,11)	10 (9,10)	-5.4	11	1 (1,2)	2 (2,2)	45.3	28	45 (41,50)	57 (49,64)	26.9	25
Pennsylvania	2 (2,2)	2 (2,2)	22.9	48	10 (9,12)	12 (11,13)	13.5	47	1 (1,1)	2 (2,2)	46.6	31	36 (33,38)	46 (40,52)	29.1	29
Rhode Island	2 (2,2)	2 (2,2)	7.7	9	9 (8,10)	8 (7,8)	-13.5	4	1 (1,1)	2 (1,2)	30.4	10	37 (34,40)	43 (38,49)	17	11
South Carolina	2 (2,2)	2 (2,2)	16.4	35	8 (7,9)	8 (8,9)	4.6	37	1 (1,1)	2 (1,2)	52.3	34	35 (32,38)	45 (39,51)	30.5	31
South Dakota	2 (2,2)	2 (2,2)	14.4	26	9 (8,10)	8 (7,9)	-9.8	6	1 (1,1)	2 (2,2)	59	43	40 (37,45)	57 (49,64)	42.4	41
Tennessee	2 (2,2)	2 (2,2)	19.7	43	8 (7,9)	8 (7,9)	2.8	35	1 (1,1)	2 (1,2)	56.8	41	34 (31,37)	46 (40,53)	36.5	36
Texas	2 (2,2)	2 (2,2)	9.4	10	8 (8,9)	9 (8,10)	5.9	39	1 (1,1)	1 (1,2)	26.9	7	32 (30,34)	37 (32,41)	12.9	6
Utah	2 (2,2)	2 (2,2)	11.9	18	10 (9,11)	9 (9,10)	-7.5	9	1 (1,2)	2 (2,2)	45.7	30	41 (38,44)	53 (46,60)	29.7	30
Vermont	2 (2,2)	3 (2,3)	13.4	22	13 (12,14)	15 (14,17)	17.2	50	2 (2,2)	2 (2,2)	26.5	6	47 (43,50)	55 (49,61)	18.1	13
Virginia	2 (2,2)	2 (2,2)	5.7	4	9 (8,10)	8 (7,9)	-9.7	7	1 (1,1)	2 (1,2)	30.9	11	37 (34,39)	42 (37,48)	15.9	10
Washington	2 (2,2)	3 (2,3)	10.6	15	12 (11,14)	11 (10,12)	-8.4	8	2 (1,2)	2 (2,2)	33.5	14	45 (42,49)	52 (45,59)	15.2	9
West Virginia	2 (2,2)	2 (2,2)	22.4	45	8 (7,9)	9 (8,9)	9.5	45	1 (1,1)	2 (2,2)	61.8	44	34 (31,38)	48 (42,56)	43.4	44
Wisconsin	2 (2,2)	2 (2,2)	16.6	36	10 (9,11)	10 (9,11)	-2.8	16	1 (1,1)	2 (2,2)	43.1	24	41 (38,44)	52 (45,58)	27.2	26

© 2020 GBD 2017 US Neurological Disorders Collaborators. *JAMA Neurology*.

eTable 7. Age-adjusted incidence, prevalence, mortality, and disability-adjusted life years (DALY) rates per 100,000 for migraine by US states in 1990 and 2017, and the percentage change between 1990 and 2017

Location	Incidence rates (95% UI)				Prevalence (95% UI)				Mortality rates (95% UI)				DALY rates (95% UI)			
	1990	2017	Change	Rank	1990	2017	Change	Rank	1990	2017	Change	Rank	1990	2017	Change	Rank
Alabama	1718 (1562,1866)	1739 (1594,1881)	1.2	20	20238 (18787,21800)	20451 (18925,22012)	1.1	19	-	-	-	-	705 (451,1030)	711 (454,1021)	0.8	11
Alaska	1679 (1526,1821)	1709 (1566,1850)	1.8	47	19555 (18137,21092)	19926 (18439,21449)	1.9	49	-	-	-	-	680 (432,989)	695 (443,1001)	2.2	48
Arizona	1705 (1549,1850)	1733 (1588,1874)	1.6	44	20030 (18594,21583)	20346 (18827,21900)	1.6	44	-	-	-	-	696 (446,1009)	708 (450,1018)	1.7	41
Arkansas	1714 (1559,1863)	1735 (1590,1876)	1.2	17	20188 (18741,21750)	20378 (18857,21934)	0.9	13	-	-	-	-	704 (456,1024)	711 (452,1020)	0.9	13
California	1696 (1542,1841)	1729 (1584,1870)	1.9	49	19921 (18493,21468)	20287 (18774,21835)	1.8	48	-	-	-	-	696 (445,1004)	712 (451,1028)	2.3	50
Colorado	1704 (1549,1850)	1729 (1584,1870)	1.4	30	20021 (18583,21576)	20277 (18761,21826)	1.3	32	-	-	-	-	698 (445,1009)	710 (448,1020)	1.7	40
Connecticut	1710 (1555,1857)	1733 (1588,1875)	1.3	27	20121 (18679,21681)	20372 (18854,21925)	1.2	29	-	-	-	-	702 (444,1016)	711 (450,1036)	1.2	26
Delaware	1716 (1560,1864)	1742 (1597,1883)	1.5	38	20174 (18727,21738)	20492 (18968,22052)	1.6	43	-	-	-	-	703 (448,1022)	714 (455,1025)	1.5	32
District of Columbia	1730 (1573,1881)	1756 (1613,1899)	1.5	40	20403 (18943,21976)	20644 (19098,22227)	1.2	25	-	-	-	-	712 (455,1033)	726 (462,1051)	2	46
Florida	1709 (1554,1856)	1735 (1590,1876)	1.5	34	20113 (18673,21669)	20385 (18865,21942)	1.4	35	-	-	-	-	700 (449,1018)	710 (451,1029)	1.4	31
Georgia	1713 (1557,1860)	1743 (1598,1885)	1.7	45	20172 (18725,21732)	20525 (18992,22095)	1.8	46	-	-	-	-	704 (451,1023)	717 (456,1033)	1.9	44
Hawaii	1691 (1536,1835)	1722 (1577,1863)	1.8	48	19838 (18415,21379)	20184 (18675,21723)	1.7	45	-	-	-	-	695 (443,1011)	710 (453,1028)	2.1	47
Idaho	1704 (1549,1850)	1730 (1586,1871)	1.5	39	19987 (18548,21543)	20285 (18770,21832)	1.5	40	-	-	-	-	697 (443,1017)	709 (451,1031)	1.6	37
Illinois	1709 (1554,1856)	1734 (1590,1876)	1.5	31	20121 (18679,21678)	20375 (18854,21930)	1.3	30	-	-	-	-	703 (447,1020)	713 (454,1028)	1.4	30
Indiana	1712 (1556,1859)	1734 (1589,1876)	1.3	25	20144 (18699,21705)	20362 (18841,21917)	1.1	20	-	-	-	-	702 (447,1016)	707 (448,1015)	0.7	9
Iowa	1710 (1554,1856)	1727 (1583,1869)	1	6	20094 (18652,21653)	20246 (18734,21790)	0.8	6	-	-	-	-	704 (446,1024)	709 (451,1023)	0.8	10
Kansas	1704 (1549,1850)	1729 (1584,1870)	1.5	32	20022 (18585,21576)	20281 (18765,21829)	1.3	33	-	-	-	-	699 (450,1011)	709 (447,1018)	1.4	29
Kentucky	1712 (1557,1860)	1731 (1587,1873)	1.1	12	20159 (18714,21718)	20323 (18807,21873)	0.8	7	-	-	-	-	701 (446,1013)	702 (448,1010)	0.2	3
Louisiana	1719 (1563,1868)	1734 (1589,1875)	0.9	5	20249 (18799,21814)	20357 (18841,21907)	0.5	4	-	-	-	-	704 (449,1015)	707 (452,1021)	0.3	5
Maine	1710 (1555,1857)	1732 (1587,1873)	1.3	23	20097 (18651,21654)	20347 (18828,21899)	1.2	28	-	-	-	-	702 (443,1017)	710 (453,1022)	1.2	24
Maryland	1711 (1573,1843)	1726 (1574,1871)	0.9	4	20027 (18695,21404)	20206 (18649,21805)	0.9	11	-	-	-	-	705 (448,1016)	713 (457,1035)	1.1	20
Massachusetts	1714 (1558,1862)	1740 (1595,1882)	1.5	36	20179 (18731,21741)	20460 (18935,22021)	1.4	37	-	-	-	-	704 (451,1016)	716 (451,1035)	1.7	42
Michigan	1480 (1334,1627)	1488 (1337,1646)	0.5	2	16810 (15267,18453)	16862 (15259,18507)	0.3	2	-	-	-	-	583 (366,860)	584 (367,860)	0.2	4
Minnesota	1709 (1553,1856)	1731 (1586,1872)	1.3	26	20057 (18615,21616)	20292 (18775,21841)	1.2	24	-	-	-	-	704 (448,1024)	715 (457,1039)	1.6	36
Mississippi	1720 (1565,1869)	1739 (1594,1881)	1.1	9	20285 (18832,21849)	20458 (18933,22020)	0.9	8	-	-	-	-	710 (455,1037)	717 (457,1035)	0.9	16
Missouri	1714 (1558,1862)	1734 (1589,1875)	1.2	15	20173 (18727,21736)	20364 (18843,21918)	0.9	14	-	-	-	-	703 (446,1020)	710 (449,1026)	0.9	15
Montana	1706 (1551,1852)	1724 (1579,1865)	1.1	8	20008 (18567,21564)	20186 (18678,21726)	0.9	10	-	-	-	-	698 (451,1008)	704 (444,1020)	0.9	14
Nebraska	1709 (1553,1856)	1729 (1584,1870)	1.2	14	20073 (18631,21632)	20267 (18752,21813)	1	16	-	-	-	-	702 (445,1021)	710 (450,1019)	1.1	22

© 2020 GBD 2017 US Neurological Disorders Collaborators. *JAMA Neurology.*

Nevada	1696 (1542,1841)	1730 (1585,1871)	2	50	19820 (18391,21372)	20265 (18748,21816)	2.2	50	-	-	-	-	690 (443,996)	705 (449,1024)	2.2	49
New Hampshire	1710 (1555,1857)	1732 (1588,1874)	1.3	24	20071 (18626,21633)	20319 (18801,21869)	1.2	27	-	-	-	-	701 (444,1015)	709 (450,1021)	1.1	21
New Jersey	1712 (1556,1859)	1733 (1589,1875)	1.3	22	20151 (18707,21711)	20384 (18865,21936)	1.2	22	-	-	-	-	703 (450,1019)	712 (455,1030)	1.3	27
New Mexico	1711 (1555,1858)	1730 (1586,1872)	1.1	11	20103 (18660,21662)	20303 (18791,21849)	1	18	-	-	-	-	700 (447,1021)	707 (452,1014)	1	17
New York	1715 (1559,1863)	1738 (1593,1880)	1.3	28	20216 (18770,21778)	20451 (18927,22012)	1.2	23	-	-	-	-	703 (451,1023)	712 (452,1033)	1.3	28
North Carolina	1709 (1554,1856)	1740 (1595,1882)	1.8	46	20132 (18690,21688)	20487 (18956,22054)	1.8	47	-	-	-	-	704 (450,1016)	717 (455,1039)	1.9	45
North Dakota	1698 (1543,1843)	1717 (1572,1857)	1.1	10	19914 (18482,21461)	20059 (18563,21589)	0.7	5	-	-	-	-	697 (446,1013)	702 (446,1013)	0.7	7
Ohio	1715 (1560,1864)	1735 (1590,1877)	1.1	13	20195 (18748,21758)	20384 (18862,21940)	0.9	12	-	-	-	-	703 (448,1018)	708 (452,1018)	0.7	8
Oklahoma	1708 (1553,1855)	1729 (1585,1871)	1.2	21	20100 (18660,21658)	20290 (18775,21838)	0.9	15	-	-	-	-	699 (443,1013)	704 (448,1015)	0.7	6
Oregon	1707 (1551,1853)	1733 (1588,1874)	1.5	42	20040 (18599,21597)	20341 (18821,21896)	1.5	41	-	-	-	-	700 (447,1016)	712 (452,1031)	1.7	39
Pennsylvania	1610 (1461,1757)	1617 (1468,1772)	0.5	1	18617 (17174,20208)	18621 (17130,20242)	0	1	-	-	-	-	648 (412,945)	646 (412,928)	-0.4	1
Rhode Island	1712 (1556,1860)	1738 (1593,1879)	1.5	35	20160 (18714,21719)	20438 (18917,21993)	1.4	36	-	-	-	-	703 (448,1021)	714 (456,1033)	1.5	33
South Carolina	1713 (1557,1860)	1737 (1592,1879)	1.4	29	20170 (18724,21728)	20425 (18904,21983)	1.3	31	-	-	-	-	702 (446,1020)	711 (450,1025)	1.2	25
South Dakota	1706 (1550,1852)	1724 (1579,1865)	1	7	20005 (18566,21560)	20181 (18674,21719)	0.9	9	-	-	-	-	701 (447,1019)	707 (450,1030)	0.9	12
Tennessee	1714 (1559,1862)	1740 (1595,1882)	1.5	37	20197 (18749,21757)	20461 (18933,22025)	1.3	34	-	-	-	-	704 (449,1019)	711 (453,1030)	1.1	19
Texas	1707 (1552,1853)	1733 (1589,1875)	1.5	41	20066 (18626,21622)	20360 (18839,21916)	1.5	39	-	-	-	-	700 (448,1007)	712 (454,1024)	1.6	38
Utah	1710 (1554,1857)	1731 (1586,1872)	1.2	18	20068 (18625,21629)	20288 (18773,21835)	1.1	21	-	-	-	-	700 (446,1012)	707 (449,1016)	1.1	18
Vermont	1710 (1555,1857)	1731 (1586,1872)	1.2	19	20084 (18639,21644)	20322 (18803,21872)	1.2	26	-	-	-	-	703 (448,1019)	714 (454,1028)	1.5	35
Virginia	1707 (1551,1853)	1734 (1590,1876)	1.6	43	20075 (18635,21631)	20384 (18863,21940)	1.5	42	-	-	-	-	700 (447,1019)	713 (454,1026)	1.8	43
Washington	1703 (1548,1849)	1728 (1584,1870)	1.5	33	19986 (18548,21542)	20270 (18756,21818)	1.4	38	-	-	-	-	698 (444,1014)	708 (449,1021)	1.5	34
West Virginia	1715 (1560,1862)	1729 (1584,1870)	0.8	3	20194 (18744,21755)	20271 (18756,21817)	0.4	3	-	-	-	-	702 (449,1016)	700 (444,1006)	-0.4	2
Wisconsin	1709 (1553,1856)	1729 (1585,1871)	1.2	16	20069 (18627,21628)	20268 (18753,21814)	1	17	-	-	-	-	701 (444,1018)	709 (451,1032)	1.2	23

© 2020 GBD 2017 US Neurological Disorders Collaborators. *JAMA Neurology*.

eTable 8. Age-adjusted incidence, prevalence, mortality, and disability-adjusted life years (DALY) rates per 100,000 for tension-type headache by US states in 1990 and 2017, and the percentage change between 1990 and 2017

Location	Incidence rates (95% UI)				Prevalence (95% UI)				Mortality rates (95% UI)				DALY rates (95% UI)			
	1990	2017	Change	Rank	1990	2017	Change	Rank	1990	2017	Change	Rank	1990	2017	Change	Rank
Alabama	12968 (11498,14380)	13017 (11606,14432)	0.4	21	34481 (31168,38243)	34653 (31355,38117)	0.5	22.5	-	-	-	-	97 (55,154)	96 (54,154)	-0.2	7
Alaska	12951 (11486,14364)	13003 (11587,14423)	0.4	46	34435 (31115,38195)	34611 (31320,38081)	0.5	36	-	-	-	-	95 (53,154)	96 (54,154)	0.6	50
Arizona	12963 (11493,14374)	13013 (11603,14431)	0.4	43	34465 (31148,38228)	34643 (31347,38105)	0.5	43	-	-	-	-	96 (54,155)	96 (55,154)	0.1	27
Arkansas	12967 (11496,14378)	13015 (11605,14432)	0.4	19	34477 (31159,38236)	34648 (31350,38114)	0.5	18	-	-	-	-	97 (54,155)	97 (54,154)	-0.1	9
California	12961 (11496,14373)	13013 (11601,14432)	0.4	48	34462 (31147,38224)	34641 (31343,38109)	0.5	47	-	-	-	-	96 (55,153)	97 (54,154)	0.4	48
Colorado	12963 (11494,14376)	13012 (11600,14431)	0.4	24	34468 (31152,38227)	34639 (31340,38104)	0.5	21	-	-	-	-	96 (54,154)	97 (54,153)	0.3	45
Connecticut	12966 (11497,14378)	13015 (11605,14434)	0.4	33	34474 (31159,38234)	34649 (31351,38115)	0.5	35	-	-	-	-	97 (55,155)	97 (54,155)	0.1	26
Delaware	12967 (11495,14380)	13017 (11608,14434)	0.4	40	34475 (31160,38239)	34654 (31360,38114)	0.5	45	-	-	-	-	97 (54,156)	97 (55,154)	0.2	33
District of Columbia	12973 (11501,14386)	13021 (11617,14431)	0.4	18	34491 (31184,38255)	34665 (31361,38129)	0.5	29	-	-	-	-	97 (55,154)	97 (54,157)	0.5	49
Florida	12965 (11494,14376)	13014 (11604,14431)	0.4	37	34470 (31153,38233)	34647 (31351,38110)	0.5	41	-	-	-	-	96 (54,153)	96 (54,153)	0.1	20
Georgia	12967 (11499,14379)	13018 (11608,14431)	0.4	44	34479 (31166,38241)	34659 (31362,38120)	0.5	46	-	-	-	-	97 (55,154)	97 (55,155)	0.3	44
Hawaii	12956 (11488,14366)	13010 (11598,14433)	0.4	50	34450 (31129,38217)	34634 (31336,38101)	0.5	49	-	-	-	-	96 (54,155)	97 (55,155)	0.4	47
Idaho	12961 (11490,14373)	13012 (11600,14432)	0.4	45	34461 (31142,38223)	34639 (31340,38104)	0.5	44	-	-	-	-	96 (54,155)	97 (54,154)	0.3	41
Illinois	12966 (11498,14378)	13015 (11604,14431)	0.4	27	34476 (31162,38235)	34648 (31348,38115)	0.5	22.5	-	-	-	-	97 (54,155)	97 (55,154)	0.2	39
Indiana	12967 (11497,14379)	13015 (11603,14431)	0.4	15.5	34477 (31163,38237)	34647 (31347,38114)	0.5	15	-	-	-	-	96 (54,155)	96 (55,153)	-0.1	8
Iowa	12965 (11495,14377)	13012 (11600,14432)	0.4	3	34473 (31159,38234)	34638 (31336,38106)	0.5	4	-	-	-	-	97 (55,156)	97 (55,154)	0	16
Kansas	12964 (11496,14376)	13012 (11600,14431)	0.4	22	34469 (31157,38231)	34640 (31339,38108)	0.5	17	-	-	-	-	96 (55,154)	97 (54,155)	0.1	31
Kentucky	12967 (11497,14378)	13014 (11603,14432)	0.4	7	34478 (31163,38236)	34645 (31343,38113)	0.5	6	-	-	-	-	96 (54,155)	96 (54,153)	-0.5	2
Louisiana	12968 (11496,14380)	13015 (11604,14433)	0.4	5	34481 (31167,38240)	34647 (31344,38118)	0.5	5	-	-	-	-	96 (54,154)	96 (55,155)	-0.3	5
Maine	12965 (11495,14377)	13015 (11604,14434)	0.4	35	34473 (31157,38230)	34647 (31350,38112)	0.5	32	-	-	-	-	97 (55,155)	97 (54,155)	0.1	19
Maryland	12941 (11519,14318)	12975 (11526,14352)	0.3	1	34281 (30987,37800)	34412 (31086,38237)	0.4	1	-	-	-	-	96 (54,153)	96 (54,154)	0.2	36
Massachusetts	12968 (11498,14381)	13017 (11607,14433)	0.4	31	34479 (31167,38242)	34654 (31356,38119)	0.5	34	-	-	-	-	97 (55,154)	97 (55,156)	0.2	37
Michigan	12967 (11496,14378)	13015 (11604,14432)	0.4	20	34476 (31160,38235)	34648 (31351,38112)	0.5	24	-	-	-	-	97 (54,154)	96 (54,155)	-0.1	10
Minnesota	12965 (11494,14377)	13012 (11600,14431)	0.4	14	34469 (31154,38231)	34639 (31338,38107)	0.5	16	-	-	-	-	97 (55,154)	97 (55,155)	0.2	35
Mississippi	12969 (11497,14380)	13017 (11607,14433)	0.4	17	34484 (31170,38245)	34655 (31355,38122)	0.5	19.5	-	-	-	-	97 (55,154)	97 (55,154)	0.1	22
Missouri	12967 (11497,14379)	13015 (11604,14432)	0.4	12	34478 (31163,38238)	34647 (31348,38114)	0.5	14	-	-	-	-	97 (54,154)	96 (54,154)	-0.1	11
Montana	12962 (11491,14373)	13009 (11598,14432)	0.4	9	34464 (31143,38219)	34631 (31338,38098)	0.5	8	-	-	-	-	96 (54,154)	96 (54,153)	0.1	21
Nebraska	12965 (11495,14376)	13012 (11600,14432)	0.4	15.5	34471 (31156,38232)	34639 (31339,38106)	0.5	11	-	-	-	-	97 (54,154)	97 (54,155)	0.2	32

© 2020 GBD 2017 US Neurological Disorders Collaborators. *JAMA Neurology*.

Nevada	12958 (11491,14371)	13011 (11599,14429)	0.4	49	34448 (31127,38214)	34636 (31338,38101)	0.5	50	-	-	-	-	96 (53,154)	96 (54,154)	0.2	34
New Hampshire	12965 (11494,14378)	13012 (11601,14432)	0.4	10	34470 (31155,38230)	34641 (31342,38106)	0.5	19.5	-	-	-	-	96 (54,153)	96 (55,155)	0.1	30
New Jersey	12967 (11498,14379)	13015 (11606,14434)	0.4	29	34477 (31163,38237)	34650 (31351,38116)	0.5	30	-	-	-	-	97 (54,154)	97 (54,155)	0.1	23
New Mexico	12964 (11492,14375)	13013 (11602,14432)	0.4	34	34467 (31148,38227)	34641 (31342,38108)	0.5	33	-	-	-	-	96 (54,155)	96 (54,155)	-0.1	12
New York	12968 (11499,14380)	13017 (11607,14433)	0.4	28	34481 (31169,38242)	34655 (31358,38122)	0.5	31	-	-	-	-	97 (54,154)	96 (54,154)	-0.1	13
North Carolina	12966 (11498,14378)	13017 (11606,14431)	0.4	47	34476 (31163,38240)	34656 (31359,38118)	0.5	48	-	-	-	-	97 (54,154)	97 (54,156)	0.4	46
North Dakota	12960 (11493,14372)	13007 (11594,14431)	0.4	4	34461 (31145,38222)	34623 (31331,38097)	0.5	3	-	-	-	-	96 (54,155)	96 (54,155)	0	15
Ohio	12968 (11497,14380)	13015 (11604,14432)	0.4	11	34479 (31165,38238)	34649 (31350,38115)	0.5	12	-	-	-	-	96 (55,155)	96 (54,153)	-0.2	6
Oklahoma	12965 (11496,14377)	13013 (11601,14431)	0.4	13	34474 (31158,38233)	34642 (31341,38111)	0.5	10	-	-	-	-	96 (54,155)	96 (54,153)	-0.3	4
Oregon	12963 (11493,14375)	13014 (11602,14431)	0.4	42	34466 (31147,38226)	34644 (31346,38107)	0.5	42	-	-	-	-	97 (55,155)	97 (54,154)	0.2	38
Pennsylvania	12968 (11498,14380)	13014 (11604,14432)	0.4	6	34479 (31167,38240)	34646 (31346,38114)	0.5	7	-	-	-	-	97 (54,154)	96 (53,153)	-0.4	3
Rhode Island	12968 (11498,14380)	13017 (11607,14435)	0.4	38	34478 (31168,38241)	34654 (31354,38121)	0.5	38	-	-	-	-	97 (54,155)	97 (54,155)	0.1	29
South Carolina	12966 (11497,14378)	13015 (11605,14432)	0.4	32	34477 (31164,38239)	34650 (31352,38115)	0.5	28	-	-	-	-	96 (54,155)	96 (54,154)	0	17
South Dakota	12962 (11492,14374)	13010 (11598,14432)	0.4	23	34463 (31149,38228)	34633 (31335,38102)	0.5	13	-	-	-	-	97 (54,155)	97 (54,153)	0	18
Tennessee	12968 (11498,14379)	13016 (11606,14431)	0.4	26	34480 (31165,38240)	34653 (31354,38117)	0.5	27	-	-	-	-	97 (55,155)	96 (54,154)	-0.1	14
Texas	12964 (11496,14377)	13014 (11603,14431)	0.4	36	34470 (31156,38232)	34646 (31349,38111)	0.5	37	-	-	-	-	96 (54,154)	97 (54,155)	0.1	28
Utah	12964 (11493,14377)	13013 (11601,14433)	0.4	25	34467 (31150,38234)	34639 (31341,38101)	0.5	26	-	-	-	-	96 (54,153)	96 (54,155)	0.1	25
Vermont	12965 (11493,14376)	13013 (11602,14433)	0.4	30	34471 (31154,38228)	34644 (31346,38110)	0.5	25	-	-	-	-	97 (54,155)	97 (54,154)	0.3	42
Virginia	12964 (11496,14377)	13015 (11604,14431)	0.4	41	34471 (31157,38232)	34648 (31350,38114)	0.5	40	-	-	-	-	96 (54,155)	97 (54,155)	0.3	43
Washington	12962 (11493,14375)	13012 (11601,14431)	0.4	39	34463 (31144,38223)	34639 (31341,38104)	0.5	39	-	-	-	-	96 (54,155)	97 (54,155)	0.3	40
West Virginia	12968 (11495,14379)	13012 (11601,14432)	0.3	2	34481 (31166,38237)	34640 (31337,38107)	0.5	2	-	-	-	-	96 (54,154)	96 (54,154)	-0.7	1
Wisconsin	12965 (11495,14377)	13012 (11600,14432)	0.4	8	34470 (31156,38231)	34638 (31336,38107)	0.5	9	-	-	-	-	96 (54,155)	97 (54,155)	0.1	24

© 2020 GBD 2017 US Neurological Disorders Collaborators. *JAMA Neurology.*

eTable 9. Age-adjusted incidence, prevalence, mortality, and disability-adjusted life years (DALY) rates per 100,000 for traumatic brain injury by US states in 1990 and 2017, and the percentage change between 1990 and 2017

Location	Incidence rates (95% UI)				Prevalence (95% UI)				Mortality rates (95% UI)				DALY rates (95% UI)			
	1990	2017	Change	Rank	1990	2017	Change	Rank	1990	2017	Change	Rank	1990	2017	Change	Rank
Alabama	424 (354,510)	289 (242,346)	-31.9	34	724 (689,757)	498 (475,522)	-31.1	32	-	-	-	-	-	-	-	-
Alaska	519 (436,627)	320 (267,386)	-38.3	49	911 (869,954)	551 (525,575)	-39.6	48	-	-	-	-	-	-	-	-
Arizona	439 (371,521)	310 (259,369)	-29.3	47	777 (742,810)	547 (521,573)	-29.6	46	-	-	-	-	-	-	-	-
Arkansas	454 (385,543)	354 (297,421)	-22.1	50	792 (755,829)	618 (589,647)	-22	50	-	-	-	-	-	-	-	-
California	427 (361,509)	310 (258,371)	-27.5	46	756 (721,790)	557 (532,585)	-26.3	49	-	-	-	-	-	-	-	-
Colorado	426 (359,511)	307 (255,366)	-28	45	743 (710,776)	532 (508,557)	-28.4	43	-	-	-	-	-	-	-	-
Connecticut	382 (320,460)	272 (225,326)	-28.8	11	671 (640,702)	485 (462,508)	-27.7	17	-	-	-	-	-	-	-	-
Delaware	386 (324,460)	278 (233,332)	-27.9	21	677 (645,707)	495 (472,518)	-26.9	26	-	-	-	-	-	-	-	-
District of Columbia	412 (346,493)	272 (227,326)	-33.9	12	672 (643,701)	474 (452,496)	-29.5	9	-	-	-	-	-	-	-	-
Florida	405 (343,479)	300 (251,358)	-25.8	44	718 (686,748)	534 (509,560)	-25.6	44	-	-	-	-	-	-	-	-
Georgia	424 (356,509)	284 (237,338)	-33.1	28	729 (695,762)	498 (474,522)	-31.8	30	-	-	-	-	-	-	-	-
Hawaii	365 (308,441)	262 (218,312)	-28.3	3	645 (615,675)	468 (447,490)	-27.4	6	-	-	-	-	-	-	-	-
Idaho	425 (357,515)	283 (235,339)	-33.5	26	742 (707,774)	495 (471,519)	-33.3	27	-	-	-	-	-	-	-	-
Illinois	397 (335,473)	275 (229,328)	-30.9	16	694 (662,726)	490 (464,514)	-29.5	23	-	-	-	-	-	-	-	-
Indiana	393 (328,470)	278 (233,332)	-29.1	22	683 (651,715)	489 (465,513)	-28.5	21	-	-	-	-	-	-	-	-
Iowa	390 (326,475)	274 (227,330)	-29.6	14	676 (643,707)	472 (448,494)	-30.2	8	-	-	-	-	-	-	-	-
Kansas	403 (337,484)	286 (238,342)	-28.9	31	706 (673,738)	505 (480,530)	-28.4	34	-	-	-	-	-	-	-	-
Kentucky	403 (337,489)	286 (239,342)	-29.1	30	688 (656,718)	491 (468,514)	-28.6	24	-	-	-	-	-	-	-	-
Louisiana	405 (339,485)	283 (237,337)	-30.1	27	698 (668,729)	496 (473,520)	-28.9	28	-	-	-	-	-	-	-	-
Maine	364 (304,439)	266 (222,319)	-27	4	631 (602,659)	461 (437,483)	-26.9	5	-	-	-	-	-	-	-	-
Maryland	410 (346,490)	314 (265,375)	-23.4	48	712 (679,745)	548 (522,575)	-23	47	-	-	-	-	-	-	-	-
Massachusetts	365 (306,440)	270 (225,324)	-26	7	637 (608,667)	480 (457,503)	-24.7	13	-	-	-	-	-	-	-	-
Michigan	394 (334,470)	294 (248,351)	-25.5	37	696 (664,727)	524 (498,550)	-24.7	41	-	-	-	-	-	-	-	-
Minnesota	391 (328,472)	275 (228,331)	-29.5	17	683 (650,713)	489 (465,512)	-28.5	22	-	-	-	-	-	-	-	-
Mississippi	424 (355,511)	288 (241,343)	-32.1	32	722 (689,756)	494 (471,517)	-31.5	25	-	-	-	-	-	-	-	-
Missouri	416 (351,501)	290 (242,346)	-30.4	35	718 (685,752)	505 (481,528)	-29.6	35	-	-	-	-	-	-	-	-
Montana	421 (353,508)	298 (248,359)	-29.2	42	731 (697,763)	516 (491,539)	-29.4	38	-	-	-	-	-	-	-	-
Nebraska	398 (334,482)	288 (241,344)	-27.7	33	695 (662,726)	511 (486,534)	-26.5	36	-	-	-	-	-	-	-	-

© 2020 GBD 2017 US Neurological Disorders Collaborators. *JAMA Neurology*.

Nevada	418 (355,497)	298 (250,354)	-28.7	43	743 (710,774)	535 (509,560)	-28	45	-	-	-	-	-	-	-	-
New Hampshire	371 (310,449)	271 (225,327)	-26.9	9	642 (612,671)	474 (453,497)	-26.1	10	-	-	-	-	-	-	-	-
New Jersey	370 (311,447)	276 (230,333)	-25.3	19	642 (611,672)	487 (463,511)	-24.1	19	-	-	-	-	-	-	-	-
New Mexico	431 (363,519)	297 (248,356)	-31.1	41	747 (712,780)	512 (488,537)	-31.4	37	-	-	-	-	-	-	-	-
New York	377 (317,455)	245 (205,294)	-34.8	1	648 (617,677)	424 (405,444)	-34.7	2	-	-	-	-	-	-	-	-
North Carolina	416 (351,496)	297 (249,354)	-28.7	40	731 (697,763)	529 (505,554)	-27.6	42	-	-	-	-	-	-	-	-
North Dakota	386 (322,469)	295 (246,355)	-23.6	38	671 (639,702)	519 (492,545)	-22.6	40	-	-	-	-	-	-	-	-
Ohio	384 (322,463)	280 (233,335)	-27.2	24	665 (634,695)	487 (464,511)	-26.7	18	-	-	-	-	-	-	-	-
Oklahoma	412 (349,496)	293 (245,350)	-29	36	714 (681,746)	505 (481,529)	-29.3	33	-	-	-	-	-	-	-	-
Oregon	410 (344,493)	270 (226,325)	-34.1	8	717 (683,748)	479 (455,501)	-33.2	12	-	-	-	-	-	-	-	-
Pennsylvania	381 (322,460)	278 (232,333)	-27.2	20	661 (630,692)	488 (464,510)	-26.2	20	-	-	-	-	-	-	-	-
Rhode Island	368 (309,445)	272 (226,326)	-26.2	10	636 (608,665)	478 (456,501)	-24.9	11	-	-	-	-	-	-	-	-
South Carolina	405 (340,484)	285 (238,341)	-29.6	29	698 (665,729)	498 (473,521)	-28.7	31	-	-	-	-	-	-	-	-
South Dakota	402 (337,486)	296 (246,353)	-26.4	39	695 (662,726)	518 (494,542)	-25.4	39	-	-	-	-	-	-	-	-
Tennessee	413 (346,496)	278 (232,333)	-32.6	23	710 (677,741)	483 (460,505)	-31.9	14	-	-	-	-	-	-	-	-
Texas	414 (347,495)	281 (234,334)	-32.2	25	721 (687,753)	497 (472,522)	-31	29	-	-	-	-	-	-	-	-
Utah	394 (332,474)	275 (229,329)	-30.3	15	694 (662,724)	483 (461,505)	-30.4	16	-	-	-	-	-	-	-	-
Vermont	384 (321,470)	266 (221,322)	-30.8	5	655 (625,686)	460 (439,482)	-29.8	4	-	-	-	-	-	-	-	-
Virginia	395 (331,477)	272 (228,326)	-31.2	13	684 (652,714)	483 (461,506)	-29.4	15	-	-	-	-	-	-	-	-
Washington	391 (329,472)	246 (203,297)	-37	2	680 (649,711)	417 (398,437)	-38.7	1	-	-	-	-	-	-	-	-
West Virginia	394 (330,474)	276 (231,332)	-29.9	18	674 (642,704)	471 (448,493)	-30.1	7	-	-	-	-	-	-	-	-
Wisconsin	391 (328,475)	270 (224,326)	-31.1	6	673 (643,705)	458 (436,481)	-32	3	-	-	-	-	-	-	-	-

© 2020 GBD 2017 US Neurological Disorders Collaborators. *JAMA Neurology*.

eTable 10. Age-adjusted incidence, prevalence, mortality, and disability-adjusted life years (DALY) rates per 100,000 for spinal cord injuries by US states in 1990 and 2017, and the percentage change between 1990 and 2017

Location	Incidence rates (95% UI)				Prevalence (95% UI)				Mortality rates (95% UI)				DALY rates (95% UI)			
	1990	2017	Change	Rank	1990	2017	Change	Rank	1990	2017	Change	Rank	1990	2017	Change	Rank
Alabama	30 (24,37)	21 (16,26)	-32	30	867 (783,966)	508 (471,548)	-41.4	10	-	-	-	-	-	-	-	-
Alaska	42 (34,51)	26 (21,33)	-37.4	50	1215 (1103,1346)	672 (620,728)	-44.6	50	-	-	-	-	-	-	-	-
Arizona	33 (27,42)	23 (18,30)	-30.2	47	976 (882,1096)	585 (542,629)	-40.1	46	-	-	-	-	-	-	-	-
Arkansas	33 (27,40)	25 (20,32)	-22.9	49	951 (864,1057)	630 (583,680)	-33.7	49	-	-	-	-	-	-	-	-
California	31 (25,38)	22 (17,28)	-28.8	42	919 (834,1032)	597 (553,644)	-35.1	47	-	-	-	-	-	-	-	-
Colorado	34 (27,43)	24 (19,31)	-28.9	48	1021 (920,1150)	622 (573,673)	-39.1	48	-	-	-	-	-	-	-	-
Connecticut	28 (23,35)	20 (15,25)	-30.9	6	854 (771,952)	528 (484,571)	-38.1	26	-	-	-	-	-	-	-	-
Delaware	28 (23,35)	20 (16,25)	-30.3	9	824 (747,915)	513 (473,556)	-37.8	13	-	-	-	-	-	-	-	-
District of Columbia	31 (25,39)	20 (16,26)	-36.1	15	781 (704,872)	509 (470,550)	-34.8	11	-	-	-	-	-	-	-	-
Florida	30 (24,37)	22 (18,28)	-26.3	44	877 (795,975)	574 (529,622)	-34.5	44	-	-	-	-	-	-	-	-
Georgia	31 (25,38)	20 (16,26)	-34.4	22	875 (792,971)	517 (477,556)	-40.9	16	-	-	-	-	-	-	-	-
Hawaii	27 (22,34)	19 (15,24)	-30.6	1	825 (749,919)	518 (477,561)	-37.2	19	-	-	-	-	-	-	-	-
Idaho	32 (26,39)	21 (17,27)	-33.9	33	946 (859,1055)	544 (503,588)	-42.5	33	-	-	-	-	-	-	-	-
Illinois	29 (23,36)	19 (15,25)	-33.3	4	850 (772,947)	514 (473,555)	-39.6	14	-	-	-	-	-	-	-	-
Indiana	29 (23,35)	20 (16,25)	-31.2	11	855 (774,948)	507 (469,547)	-40.7	8	-	-	-	-	-	-	-	-
Iowa	31 (24,38)	22 (17,28)	-29.3	39	926 (835,1040)	546 (505,592)	-41	34	-	-	-	-	-	-	-	-
Kansas	29 (23,36)	20 (16,26)	-29.7	25	873 (793,966)	532 (491,575)	-39.1	28	-	-	-	-	-	-	-	-
Kentucky	30 (24,37)	21 (16,26)	-30.2	31	859 (774,970)	506 (467,546)	-41.1	7	-	-	-	-	-	-	-	-
Louisiana	29 (23,35)	20 (16,25)	-31.5	7	811 (734,899)	496 (459,537)	-38.8	3	-	-	-	-	-	-	-	-
Maine	29 (23,36)	20 (16,26)	-30	14	864 (778,974)	517 (477,561)	-40.1	18	-	-	-	-	-	-	-	-
Maryland	31 (25,39)	23 (18,29)	-27.4	46	912 (825,1036)	578 (535,624)	-36.6	45	-	-	-	-	-	-	-	-
Massachusetts	28 (23,35)	20 (15,25)	-30.2	5	841 (761,938)	523 (483,567)	-37.8	23	-	-	-	-	-	-	-	-
Michigan	30 (24,37)	21 (17,27)	-27.7	36	872 (791,976)	559 (516,603)	-35.8	41	-	-	-	-	-	-	-	-
Minnesota	30 (24,37)	20 (16,26)	-32.4	19	896 (814,994)	542 (500,583)	-39.5	32	-	-	-	-	-	-	-	-
Mississippi	30 (24,37)	21 (16,26)	-31.9	29	852 (771,950)	501 (464,543)	-41.2	4	-	-	-	-	-	-	-	-
Missouri	31 (25,38)	21 (16,27)	-32.1	32	893 (809,995)	528 (487,567)	-41	24	-	-	-	-	-	-	-	-
Montana	32 (26,40)	22 (17,28)	-31.2	43	942 (859,1048)	552 (510,595)	-41.5	39	-	-	-	-	-	-	-	-
Nebraska	29 (23,36)	20 (16,26)	-29.4	27	866 (786,959)	540 (500,583)	-37.7	31	-	-	-	-	-	-	-	-

© 2020 GBD 2017 US Neurological Disorders Collaborators. *JAMA Neurology.*

Nevada	31 (25,38)	22 (17,27)	-29.4	40	896 (812,999)	572 (530,616)	-36.2	43	-	-	-	-	-	-	-	-
New Hampshire	29 (23,36)	20 (16,26)	-29.8	23	868 (786,969)	528 (488,569)	-39.1	25	-	-	-	-	-	-	-	-
New Jersey	29 (23,36)	20 (16,26)	-28.4	28	852 (766,956)	558 (516,605)	-34.5	40	-	-	-	-	-	-	-	-
New Mexico	33 (26,41)	22 (17,28)	-32.3	45	940 (852,1046)	549 (508,593)	-41.6	35	-	-	-	-	-	-	-	-
New York	30 (24,37)	19 (15,25)	-34.8	2	857 (771,974)	502 (462,542)	-41.4	5	-	-	-	-	-	-	-	-
North Carolina	31 (25,38)	22 (17,28)	-28.8	41	898 (815,1001)	569 (525,614)	-36.6	42	-	-	-	-	-	-	-	-
North Dakota	29 (23,36)	21 (17,27)	-26.5	34	869 (789,972)	550 (512,597)	-36.6	37	-	-	-	-	-	-	-	-
Ohio	29 (23,36)	20 (16,26)	-29.6	17	845 (761,945)	508 (468,551)	-39.9	9	-	-	-	-	-	-	-	-
Oklahoma	30 (25,38)	21 (17,27)	-29.9	35	889 (807,994)	521 (483,566)	-41.3	22	-	-	-	-	-	-	-	-
Oregon	30 (24,37)	20 (16,25)	-34.9	8	894 (812,995)	520 (481,561)	-41.9	20	-	-	-	-	-	-	-	-
Pennsylvania	29 (23,35)	20 (16,26)	-30.1	12	843 (760,933)	517 (477,558)	-38.7	17	-	-	-	-	-	-	-	-
Rhode Island	29 (23,36)	20 (16,26)	-30.4	16	862 (781,975)	529 (488,570)	-38.6	27	-	-	-	-	-	-	-	-
South Carolina	29 (24,36)	20 (16,26)	-31.2	18	838 (762,934)	506 (466,545)	-39.7	6	-	-	-	-	-	-	-	-
South Dakota	30 (24,37)	21 (17,27)	-28.5	37	884 (803,973)	551 (509,594)	-37.7	38	-	-	-	-	-	-	-	-
Tennessee	29 (24,36)	20 (15,25)	-32.5	10	838 (760,930)	492 (453,532)	-41.3	1	-	-	-	-	-	-	-	-
Texas	30 (24,37)	20 (16,25)	-33	13	872 (790,968)	521 (483,563)	-40.3	21	-	-	-	-	-	-	-	-
Utah	30 (24,38)	20 (16,26)	-32.6	26	910 (824,1019)	536 (496,578)	-41.2	30	-	-	-	-	-	-	-	-
Vermont	30 (24,38)	20 (16,26)	-32.4	24	887 (802,988)	533 (491,578)	-39.9	29	-	-	-	-	-	-	-	-
Virginia	29 (23,36)	19 (15,25)	-32.8	3	844 (766,942)	514 (474,556)	-39	15	-	-	-	-	-	-	-	-
Washington	31 (25,39)	20 (16,26)	-34.8	20	925 (837,1037)	512 (473,552)	-44.7	12	-	-	-	-	-	-	-	-
West Virginia	29 (24,37)	20 (16,26)	-31.2	21	851 (771,951)	493 (455,532)	-42.1	2	-	-	-	-	-	-	-	-
Wisconsin	31 (25,39)	22 (17,28)	-30.7	38	931 (836,1052)	550 (506,596)	-40.9	36	-	-	-	-	-	-	-	-

© 2020 GBD 2017 US Neurological Disorders Collaborators. *JAMA Neurology*.

eTable 11. Age-adjusted incidence, prevalence, mortality, and disability-adjusted life years (DALY) rates per 100,000 for brain and nervous system cancer by US states in 1990 and 2017, and the percentage change between 1990 and 2017

Location	Incidence rates (95% UI)				Prevalence (95% UI)				Mortality rates (95% UI)				DALY rates (95% UI)			
	1990	2017	Change	Rank	1990	2017	Change	Rank	1990	2017	Change	Rank	1990	2017	Change	Rank
Alabama	5 (4,6)	7 (5,8)	28.8	35	17 (14,22)	28 (21,35)	60.5	35	4 (3,4)	4 (3,5)	8.4	46	139 (111,151)	143 (115,166)	2.5	42
Alaska	5 (5,6)	7 (6,9)	32.9	42	20 (16,25)	35 (27,44)	77.2	46	4 (3,4)	3 (3,4)	-5.7	15	123 (113,145)	117 (101,138)	-5.2	25
Arizona	5 (5,6)	7 (6,8)	28.7	33	21 (17,26)	32 (25,40)	54.8	27	3 (3,4)	4 (3,4)	1.5	30	122 (108,136)	115 (101,135)	-5.4	24
Arkansas	6 (4,7)	7 (5,9)	25.6	22	19 (13,25)	30 (22,38)	54.7	26	4 (3,5)	4 (3,5)	4.9	37	148 (109,161)	148 (114,174)	0.1	37
California	6 (5,7)	7 (6,9)	18.8	7	23 (20,27)	35 (28,44)	50.3	22	4 (3,4)	3 (3,4)	-8.6	5	132 (110,140)	111 (96,133)	-16	2
Colorado	6 (5,7)	8 (6,10)	29.4	36	27 (22,32)	40 (32,49)	48.8	18	4 (3,4)	4 (3,4)	-1.2	27	126 (112,136)	119 (103,139)	-5.5	23
Connecticut	7 (6,8)	7 (6,9)	0.9	1	32 (27,39)	35 (28,44)	7.1	1	4 (3,4)	3 (3,4)	-5.7	16	125 (116,144)	108 (93,134)	-13.3	5
Delaware	5 (5,6)	7 (6,9)	28.3	30	20 (16,25)	33 (25,43)	65.6	38	4 (3,4)	3 (3,4)	-6.5	10	124 (114,148)	113 (98,143)	-8.2	18
District of Columbia	4 (4,9)	5 (4,10)	21.5	10	12 (10,24)	27 (19,51)	127.1	50	4 (3,6)	3 (2,5)	-21.3	1	137 (119,243)	101 (79,173)	-25.9	1
Florida	6 (5,7)	8 (6,9)	21.9	13	26 (20,32)	38 (30,47)	45.7	14	4 (3,4)	4 (3,4)	-7	9	137 (115,146)	120 (105,140)	-12.6	8
Georgia	5 (5,6)	7 (6,8)	25.8	23	18 (16,23)	31 (25,40)	69	40	4 (3,4)	4 (3,4)	-5.3	17	128 (113,145)	114 (99,140)	-11.1	12
Hawaii	4 (3,7)	6 (4,9)	35.3	44	19 (14,30)	30 (23,47)	58.5	31	2 (2,4)	2 (2,4)	0.9	29	86 (74,130)	85 (69,133)	-0.5	35
Idaho	6 (5,7)	7 (6,9)	24.8	19	23 (17,30)	33 (25,42)	41.6	9	4 (3,4)	4 (3,5)	4.6	36	134 (105,145)	132 (102,154)	-1.6	31
Illinois	5 (5,6)	7 (6,9)	31.1	39	19 (16,24)	33 (27,44)	72.3	43	4 (3,4)	3 (3,4)	-6.2	12	127 (117,145)	113 (99,140)	-11.3	10
Indiana	6 (5,7)	7 (6,9)	24.7	18	22 (18,27)	32 (26,40)	46.4	16	4 (3,4)	4 (3,5)	3.5	33	132 (113,141)	131 (112,152)	-0.5	36
Iowa	7 (5,8)	8 (6,10)	20.2	8	29 (22,35)	39 (30,48)	35.9	3	4 (3,4)	4 (3,5)	8.2	45	135 (109,145)	138 (110,160)	2.7	43
Kansas	6 (5,7)	8 (6,10)	27.1	26	27 (21,34)	38 (29,48)	41	8	4 (3,4)	4 (3,5)	6.9	40	136 (110,146)	139 (111,160)	2.1	41
Kentucky	6 (5,7)	7 (6,9)	24.9	20	23 (18,28)	33 (26,42)	44.6	11	4 (3,4)	4 (3,5)	2.2	32	136 (111,146)	134 (112,159)	-1	33
Louisiana	5 (4,6)	6 (5,8)	28.5	32	17 (14,21)	28 (22,36)	71.1	41	4 (3,4)	4 (3,4)	4.9	38	128 (112,150)	128 (110,157)	0.1	38
Maine	6 (5,7)	9 (7,11)	38.9	45	26 (21,32)	42 (33,53)	59.7	34	4 (3,4)	4 (3,5)	8.2	44	127 (112,137)	133 (111,156)	4.9	45
Maryland	5 (5,7)	7 (6,9)	28	28	21 (17,27)	35 (27,49)	64.4	37	4 (3,4)	3 (3,4)	-7.1	8	127 (117,152)	116 (100,154)	-8.5	17
Massachusetts	7 (6,8)	9 (7,11)	33.2	43	30 (25,38)	45 (36,57)	49.3	20	4 (4,4)	4 (3,5)	1.9	31	130 (120,146)	123 (107,147)	-5.1	26
Michigan	6 (5,7)	7 (6,9)	21.9	11	23 (18,28)	34 (28,43)	49.9	21	4 (3,4)	4 (3,5)	-1.3	25	139 (117,149)	130 (111,151)	-6.7	22
Minnesota	7 (6,8)	9 (7,11)	25.2	21	32 (26,39)	45 (36,55)	39.9	6	4 (3,4)	4 (3,4)	-2.8	21	132 (111,142)	126 (105,146)	-4.7	29
Mississippi	5 (4,6)	6 (5,8)	24.6	17	15 (11,19)	25 (19,31)	62	36	4 (3,4)	4 (3,5)	7	41	142 (109,155)	144 (113,169)	1.2	40
Missouri	6 (5,7)	7 (6,9)	27	25	22 (18,26)	34 (27,43)	57.1	30	4 (3,4)	4 (3,4)	-1.7	24	132 (114,143)	126 (109,148)	-5	28
Montana	6 (5,6)	9 (6,11)	52.2	49	22 (18,27)	42 (30,53)	87.9	49	4 (3,4)	4 (3,5)	14.5	50	129 (111,140)	143 (112,171)	10.4	48
Nebraska	7 (5,8)	8 (6,10)	28.2	29	28 (22,34)	40 (31,50)	45.2	13	4 (3,4)	4 (3,5)	4.2	34	140 (114,151)	138 (111,158)	-1	32

© 2020 GBD 2017 US Neurological Disorders Collaborators. *JAMA Neurology*.

Nevada	5 (4,6)	6 (5,8)	17.5	5	18 (15,24)	27 (21,36)	45.9	15	4 (3,4)	3 (3,4)	-6.3	11	120 (111,145)	105 (91,133)	-12.3	9
New Hampshire	6 (6,7)	9 (7,10)	32.7	41	28 (23,34)	43 (34,54)	53.7	25	4 (3,4)	4 (3,4)	-1.3	26	128 (115,140)	123 (106,142)	-4.4	30
New Jersey	6 (5,7)	7 (6,10)	20.7	9	24 (20,31)	38 (30,52)	56.3	28	4 (3,4)	3 (3,4)	-9.9	3	123 (114,150)	105 (90,139)	-14.4	4
New Mexico	4 (4,6)	6 (5,8)	40.1	46	16 (13,20)	29 (22,37)	80.2	47	3 (3,4)	3 (3,4)	4.3	35	113 (105,136)	113 (96,143)	-0.7	34
New York	5 (5,7)	7 (6,9)	31.6	40	20 (16,26)	35 (28,49)	76	45	4 (3,4)	3 (3,4)	-10	2	125 (117,154)	105 (91,141)	-15.5	3
North Carolina	5 (5,6)	6 (5,8)	18.7	6	19 (16,24)	29 (23,37)	52.7	24	4 (3,4)	4 (3,4)	-8.5	6	135 (113,146)	117 (102,139)	-13.1	6
North Dakota	6 (5,7)	10 (8,12)	52.6	50	27 (21,32)	49 (37,61)	83.7	48	4 (3,4)	5 (4,5)	11.7	48	138 (113,150)	154 (122,177)	11.4	49
Ohio	6 (5,6)	7 (6,9)	28.7	34	22 (18,27)	33 (26,41)	51.3	23	4 (3,4)	4 (3,5)	5.2	39	130 (114,140)	130 (113,153)	0.4	39
Oklahoma	6 (5,6)	7 (5,8)	22.8	14	22 (17,27)	30 (23,39)	39.1	4	4 (3,4)	4 (3,5)	7.8	42	132 (111,142)	139 (112,163)	5.1	46
Oregon	7 (5,8)	9 (7,11)	26.8	24	29 (22,37)	43 (34,53)	44.9	12	4 (3,5)	4 (3,5)	-2	22	144 (111,156)	134 (107,154)	-7.3	20
Pennsylvania	6 (5,7)	7 (6,9)	30	37	22 (18,28)	35 (28,46)	59.1	33	4 (3,4)	4 (3,4)	-1.8	23	128 (117,143)	120 (104,145)	-6.8	21
Rhode Island	7 (5,7)	8 (7,10)	21.9	12	27 (22,33)	39 (31,49)	43.8	10	4 (4,4)	4 (3,4)	-7.8	7	139 (122,149)	123 (107,144)	-11.2	11
South Carolina	5 (4,6)	7 (5,8)	24.5	15	17 (13,20)	28 (22,36)	71.4	42	4 (3,4)	4 (3,5)	-4.4	18	140 (112,152)	126 (108,152)	-9.9	16
South Dakota	6 (5,7)	9 (6,11)	44.1	48	25 (18,31)	43 (29,55)	74.4	44	4 (3,5)	5 (3,6)	13.1	49	146 (110,160)	163 (120,192)	11.4	50
Tennessee	6 (4,7)	7 (5,8)	14.2	2	21 (15,26)	29 (23,36)	40.2	7	4 (3,5)	4 (3,5)	-6.1	13	147 (110,159)	131 (112,152)	-10.4	13
Texas	6 (4,6)	6 (5,8)	16.6	4	20 (16,24)	30 (23,36)	47.7	17	4 (3,4)	4 (3,4)	-8.6	4	133 (108,142)	116 (102,134)	-12.7	7
Utah	5 (5,6)	8 (6,9)	40.9	47	22 (18,26)	36 (28,44)	66.7	39	3 (3,4)	4 (3,4)	8	43	118 (100,131)	122 (101,140)	2.9	44
Vermont	6 (5,7)	8 (7,10)	31	38	25 (20,30)	40 (32,49)	57.1	29	4 (3,4)	4 (3,4)	-4.4	19	129 (117,142)	119 (104,138)	-7.6	19
Virginia	5 (5,7)	7 (6,9)	27.9	27	21 (18,27)	34 (27,46)	58.7	32	4 (3,4)	3 (3,4)	-5.9	14	121 (113,143)	109 (94,140)	-10	15
Washington	7 (5,7)	8 (6,9)	14.5	3	27 (22,33)	37 (30,45)	35.6	2	4 (3,4)	4 (3,5)	-4.1	20	141 (113,151)	127 (102,144)	-10.3	14
West Virginia	5 (4,6)	6 (5,8)	28.4	31	18 (15,23)	27 (21,34)	49	19	4 (3,4)	4 (3,5)	10.7	47	122 (111,141)	130 (108,154)	6.7	47
Wisconsin	7 (5,8)	8 (7,10)	24.6	16	30 (24,37)	42 (33,51)	39.3	5	4 (3,4)	4 (3,5)	-0.8	28	137 (113,146)	130 (109,150)	-5	27

© 2020 GBD 2017 US Neurological Disorders Collaborators. *JAMA Neurology*.

eTable 12. Age-adjusted incidence, prevalence, mortality, and disability-adjusted life years (DALY) rates per 100,000 for meningitis by US states in 1990 and 2017, and the percentage change between 1990 and 2017

Location	Incidence rates (95% UI)				Prevalence (95% UI)				Mortality rates (95% UI)				DALY rates (95% UI)			
	1990	2017	Change	Rank	1990	2017	Change	Rank	1990	2017	Change	Rank	1990	2017	Change	Rank
Alabama	19 (17,22)	12 (11,14)	-35.3	15	40 (35,46)	20 (17,22)	-51.1	12	1 (1,1)	1 (0,1)	-53.6	47	73 (64,84)	32 (27,38)	-56.2	47
Alaska	21 (18,24)	16 (14,18)	-24.9	33	42 (36,49)	26 (23,29)	-39	39	1 (1,1)	0 (0,1)	-61.8	21	72 (59,85)	27 (22,32)	-62.6	27
Arizona	21 (18,24)	16 (14,18)	-22.6	37	44 (38,50)	27 (24,31)	-38.2	40	1 (1,1)	0 (0,0)	-61.5	23	62 (53,72)	22 (19,27)	-63.6	23
Arkansas	19 (17,22)	13 (12,15)	-31.6	21	41 (35,47)	21 (19,24)	-48.2	21	1 (1,2)	1 (0,1)	-60.5	26	87 (73,101)	31 (26,37)	-64.8	19
California	19 (16,22)	14 (12,16)	-27.1	32	40 (35,46)	23 (20,26)	-42.9	33	1 (1,1)	0 (0,0)	-74.8	2	70 (62,80)	16 (14,22)	-77.4	2
Colorado	17 (15,20)	11 (10,12)	-36.5	11	38 (33,45)	18 (16,21)	-52.4	8	1 (1,1)	0 (0,0)	-61.9	20	55 (47,66)	19 (16,22)	-65.6	17
Connecticut	24 (21,27)	19 (17,22)	-21.4	39	49 (43,56)	30 (27,35)	-38.1	41	1 (1,1)	0 (0,0)	-66.5	9	57 (46,66)	18 (16,22)	-68	11
Delaware	17 (14,20)	10 (9,11)	-40.1	3	36 (31,42)	16 (14,18)	-55.1	2	1 (1,1)	0 (0,0)	-61.5	22	58 (49,68)	21 (17,26)	-64	22
District of Columbia	30 (27,34)	20 (18,22)	-35.5	14	53 (46,62)	29 (25,33)	-45.8	27	4 (2,4)	1 (1,1)	-78.2	1	260 (148,323)	51 (38,64)	-80.5	1
Florida	20 (18,23)	16 (14,18)	-20.5	41	42 (36,48)	26 (23,30)	-37.9	42	1 (1,1)	0 (0,0)	-67.4	6	72 (62,83)	22 (18,27)	-70	6
Georgia	21 (18,24)	15 (13,17)	-29.6	28	42 (37,49)	24 (21,27)	-43.5	32	1 (1,2)	0 (0,1)	-64.7	13	80 (65,92)	26 (22,30)	-67.5	13
Hawaii	16 (13,20)	11 (10,14)	-30.4	26	37 (32,44)	20 (18,23)	-45.9	26	1 (1,1)	0 (0,0)	-61.4	24	47 (40,57)	17 (14,22)	-63.4	25
Idaho	15 (12,18)	9 (8,10)	-38.4	6	34 (29,41)	16 (14,18)	-54	5	1 (1,1)	0 (0,0)	-66.7	8	49 (43,58)	14 (12,22)	-70.9	5
Illinois	17 (15,20)	11 (10,13)	-36.6	10	37 (32,43)	18 (16,21)	-50.6	13	1 (1,1)	0 (0,0)	-67.1	7	65 (57,74)	20 (17,23)	-69.6	8
Indiana	20 (18,23)	15 (13,17)	-24.3	34	43 (37,49)	25 (22,29)	-41.8	34	1 (1,1)	0 (0,1)	-54.5	44	55 (49,63)	24 (21,30)	-56.8	45
Iowa	18 (16,21)	14 (13,16)	-20.5	42	40 (35,47)	24 (21,28)	-39.5	37	1 (1,1)	0 (0,0)	-56.4	40	43 (37,51)	18 (15,24)	-59	39
Kansas	18 (15,21)	14 (12,16)	-21.1	40	39 (34,45)	23 (20,26)	-40.9	36	1 (1,1)	0 (0,0)	-55	42	46 (40,54)	19 (16,25)	-58.8	41
Kentucky	18 (16,21)	13 (11,15)	-30	27	40 (34,46)	21 (18,24)	-46.9	24	1 (1,1)	0 (0,1)	-58.3	33	70 (62,80)	26 (22,33)	-63	26
Louisiana	20 (17,23)	10 (9,12)	-47.3	1	41 (36,48)	16 (14,19)	-60.4	1	2 (1,2)	1 (1,1)	-57.2	38	93 (69,108)	37 (31,43)	-60.4	35
Maine	16 (14,19)	11 (10,13)	-32.6	18	37 (32,43)	18 (16,21)	-49.7	17	1 (1,1)	0 (0,0)	-53.2	48	44 (39,51)	20 (17,27)	-54.1	50
Maryland	19 (16,22)	12 (11,14)	-35.9	12	38 (33,44)	19 (17,22)	-50.5	14	1 (1,1)	0 (0,0)	-64.4	14	70 (57,80)	24 (20,27)	-66.3	15
Massachusetts	26 (23,29)	21 (18,24)	-18.1	44	54 (47,62)	34 (30,39)	-36.9	45	1 (1,1)	0 (0,0)	-59.5	27	47 (40,54)	19 (17,22)	-58.6	44
Michigan	16 (13,19)	10 (9,12)	-35.1	16	34 (29,40)	17 (15,20)	-49.9	16	1 (1,1)	0 (0,0)	-59.2	32	51 (45,58)	20 (17,27)	-61.4	31
Minnesota	16 (14,19)	12 (10,13)	-28.1	30	36 (31,42)	20 (17,22)	-46.2	25	1 (1,1)	0 (0,0)	-63.4	16	42 (38,49)	15 (12,20)	-65.3	18
Mississippi	20 (18,23)	14 (12,16)	-30.9	25	42 (37,49)	22 (19,25)	-48	22	1 (1,2)	1 (1,1)	-58.1	34	94 (77,110)	36 (30,43)	-62.1	28
Missouri	20 (17,23)	15 (13,17)	-24	35	41 (36,48)	24 (21,28)	-41.2	35	1 (1,1)	0 (0,1)	-57.2	37	60 (52,70)	24 (20,29)	-60.2	36
Montana	17 (15,20)	10 (9,12)	-39.1	5	38 (33,45)	17 (15,20)	-55.1	3	1 (1,1)	0 (0,1)	-54.3	46	53 (46,62)	22 (18,28)	-59.3	38
Nebraska	16 (14,19)	11 (10,12)	-31.9	20	36 (31,42)	18 (16,21)	-49.4	18	1 (1,1)	0 (0,0)	-59.4	30	44 (38,52)	16 (13,23)	-63.5	24

© 2020 GBD 2017 US Neurological Disorders Collaborators. *JAMA Neurology*.

Nevada	22 (20,26)	18 (16,20)	-20.4	43	45 (40,52)	30 (26,34)	-33.8	49	1 (1,2)	0 (0,0)	-67.8	5	79 (63,93)	24 (20,28)	-70	7
New Hampshire	21 (18,24)	17 (15,20)	-17.8	46	45 (39,52)	28 (25,33)	-36.9	44	1 (1,1)	0 (0,0)	-58.1	35	39 (34,46)	16 (13,22)	-58.7	42
New Jersey	18 (16,21)	11 (9,12)	-41.2	2	38 (33,44)	17 (15,20)	-54.6	4	1 (1,1)	0 (0,0)	-69.8	4	66 (52,76)	19 (16,22)	-71.2	4
New Mexico	19 (17,22)	12 (11,14)	-37.1	7	41 (36,48)	20 (17,23)	-52.2	9	1 (1,1)	1 (0,1)	-57.5	36	73 (57,86)	29 (24,34)	-61	33
New York	21 (18,24)	14 (12,16)	-31.5	22	41 (36,47)	23 (20,26)	-45	30	1 (1,1)	0 (0,0)	-72.5	3	76 (59,87)	20 (18,24)	-73.3	3
North Carolina	19 (17,22)	13 (11,15)	-32.5	19	40 (35,47)	21 (18,24)	-48.2	20	1 (1,1)	0 (0,0)	-65.6	11	72 (62,85)	23 (20,28)	-67.9	12
North Dakota	16 (14,19)	12 (11,14)	-23.9	36	37 (32,43)	20 (18,23)	-45.6	29	1 (1,1)	0 (0,0)	-51.3	50	39 (34,48)	18 (15,26)	-54.3	49
Ohio	24 (21,28)	20 (18,23)	-16.9	47	50 (44,58)	33 (29,38)	-34.3	48	1 (1,1)	0 (0,1)	-54.7	43	54 (48,62)	24 (20,30)	-55.7	48
Oklahoma	18 (15,21)	11 (10,13)	-37	8	39 (34,45)	18 (16,21)	-53.4	7	1 (1,1)	1 (0,1)	-52.2	49	68 (57,80)	30 (25,36)	-56.5	46
Oregon	17 (15,20)	12 (10,13)	-31.2	24	37 (32,44)	20 (17,23)	-47.2	23	1 (1,1)	0 (0,0)	-62.9	18	53 (45,62)	18 (15,23)	-66	16
Pennsylvania	19 (16,22)	14 (12,15)	-27.4	31	40 (34,46)	22 (19,25)	-44.9	31	1 (1,1)	0 (0,0)	-59.5	28	56 (49,64)	22 (19,26)	-60.8	34
Rhode Island	22 (20,25)	18 (16,21)	-16.7	48	47 (41,55)	30 (26,34)	-36.9	46	1 (1,1)	0 (0,0)	-63	17	54 (45,64)	19 (16,23)	-64.4	21
South Carolina	19 (16,22)	12 (10,13)	-36.9	9	40 (34,46)	19 (17,22)	-51.9	11	1 (1,1)	1 (0,1)	-61.2	25	77 (64,91)	27 (23,32)	-64.8	20
South Dakota	17 (15,20)	12 (11,13)	-31.5	23	39 (33,45)	19 (17,22)	-50.1	15	1 (1,1)	0 (0,1)	-54.5	45	60 (50,71)	25 (21,30)	-58.8	40
Tennessee	25 (22,28)	20 (18,23)	-18	45	51 (45,59)	32 (28,37)	-37.1	43	1 (1,1)	0 (0,1)	-59.4	29	67 (58,76)	25 (21,32)	-62	29
Texas	19 (16,22)	12 (11,14)	-32.8	17	40 (34,46)	21 (18,24)	-48.3	19	1 (1,1)	0 (0,0)	-64.3	15	69 (56,79)	23 (20,27)	-67.2	14
Utah	17 (14,20)	11 (9,12)	-35.7	13	38 (33,44)	18 (16,21)	-52.1	10	1 (1,1)	0 (0,0)	-56.4	39	43 (38,53)	18 (15,22)	-59.6	37
Vermont	26 (23,30)	22 (20,26)	-14.8	50	56 (49,65)	37 (33,43)	-33.3	50	1 (1,1)	0 (0,0)	-62.3	19	49 (42,58)	19 (16,24)	-61.5	30
Virginia	18 (16,21)	11 (9,12)	-40	4	38 (33,45)	18 (16,20)	-53.7	6	1 (1,1)	0 (0,0)	-65.3	12	67 (55,77)	21 (18,25)	-68.1	10
Washington	19 (17,22)	15 (13,17)	-22.3	38	41 (36,47)	25 (22,28)	-39.3	38	1 (1,1)	0 (0,0)	-66.2	10	52 (46,61)	16 (14,22)	-68.9	9
West Virginia	25 (22,29)	21 (18,24)	-16.6	49	54 (47,61)	34 (30,39)	-35.8	47	1 (1,1)	0 (0,1)	-55.3	41	66 (56,78)	27 (23,36)	-58.6	43
Wisconsin	17 (14,20)	12 (11,14)	-28.3	29	37 (32,43)	20 (18,23)	-45.7	28	1 (1,1)	0 (0,0)	-59.3	31	44 (39,51)	17 (15,23)	-61.1	32

© 2020 GBD 2017 US Neurological Disorders Collaborators. *JAMA Neurology*.

eTable 13. Age-adjusted incidence, prevalence, mortality, and disability-adjusted life years (DALY) rates per 100,000 for encephalitis by US states in 1990 and 2017, and the percentage change between 1990 and 2017

Location	Incidence rates (95% UI)				Prevalence (95% UI)				Mortality rates (95% UI)				DALY rates (95% UI)			
	1990	2017	Change	Rank	1990	2017	Change	Rank	1990	2017	Change	Rank	1990	2017	Change	Rank
Alabama	5 (4,5)	5 (4,5)	0.1	28	7 (4,11)	6 (4,10)	-9.6	33	0 (0,0)	0 (0,0)	-10.1	44	13 (9,15)	11 (8,13)	-14.9	44
Alaska	4 (4,4)	4 (4,4)	-2.3	2	4 (3,6)	4 (3,5)	-11	22	0 (0,0)	0 (0,0)	-25.3	7	13 (10,15)	10 (8,11)	-26	17
Arizona	5 (5,5)	5 (5,5)	-0.1	24	8 (4,12)	7 (4,11)	-7.7	45	0 (0,0)	0 (0,0)	-22.7	12	11 (10,13)	8 (7,10)	-28.3	11
Arkansas	5 (5,5)	5 (5,5)	-0.1	25	8 (5,12)	7 (4,11)	-11.9	13	0 (0,0)	0 (0,0)	-12.1	37	13 (9,15)	11 (9,12)	-18.9	33
California	5 (5,5)	5 (5,5)	-0.7	8	7 (4,11)	6 (4,9)	-14	4	0 (0,0)	0 (0,0)	-27.7	4	8 (7,11)	5 (5,9)	-35	3
Colorado	5 (5,5)	5 (5,5)	-0.1	20	7 (4,11)	6 (4,9)	-11.5	17	0 (0,0)	0 (0,0)	-17.6	25	10 (9,12)	8 (7,9)	-24.3	24
Connecticut	5 (5,5)	5 (5,5)	0.3	34	7 (4,11)	6 (4,10)	-10.5	28	0 (0,0)	0 (0,0)	-26	6	10 (8,11)	7 (6,9)	-31.8	6
Delaware	4 (4,4)	4 (4,4)	-0.3	13	6 (3,8)	5 (3,8)	-7.2	46	0 (0,0)	0 (0,0)	-21.7	15	11 (9,12)	8 (7,10)	-25.6	19
District of Columbia	6 (6,6)	6 (6,6)	-0.2	17	7 (4,11)	6 (4,9)	-9.2	37	1 (0,1)	0 (0,0)	-48	1	41 (15,50)	18 (10,23)	-55	1
Florida	5 (5,5)	5 (5,5)	1	49	7 (4,11)	7 (4,11)	-6.2	49	0 (0,0)	0 (0,0)	-23.8	9	10 (9,11)	7 (6,9)	-29.3	7
Georgia	5 (5,5)	5 (5,5)	0.7	44	7 (4,11)	6 (4,10)	-6.9	48	0 (0,0)	0 (0,0)	-23.3	11	13 (9,14)	9 (8,10)	-29.2	9
Hawaii	3 (2,3)	3 (2,3)	-2.5	1	2 (2,4)	2 (1,3)	-21.5	1	0 (0,0)	0 (0,0)	-19.9	22	10 (9,11)	8 (7,9)	-23.9	25
Idaho	5 (5,5)	5 (5,5)	0.2	31	7 (4,11)	7 (4,10)	-9.4	35	0 (0,0)	0 (0,0)	-17	28	9 (8,10)	7 (6,9)	-25.5	20
Illinois	5 (5,5)	5 (5,5)	0.5	39	8 (4,12)	7 (4,11)	-7.9	44	0 (0,0)	0 (0,0)	-24.3	8	11 (9,12)	8 (7,9)	-29.3	8
Indiana	5 (5,5)	5 (5,5)	0.5	37	7 (4,12)	7 (4,10)	-9.9	30	0 (0,0)	0 (0,0)	-11.3	38	10 (9,11)	8 (8,10)	-15.9	40
Iowa	4 (4,4)	4 (4,5)	0.7	43	7 (4,10)	6 (3,9)	-10.7	26	0 (0,0)	0 (0,0)	-10.6	42	8 (7,10)	7 (5,10)	-16.2	39
Kansas	5 (5,5)	5 (5,5)	-1	6	8 (4,12)	7 (4,11)	-12.2	11	0 (0,0)	0 (0,0)	-10.1	45	9 (8,10)	8 (6,9)	-17	37
Kentucky	6 (6,7)	6 (6,7)	0.1	27	11 (6,17)	9 (5,15)	-11.1	20	0 (0,0)	0 (0,0)	-10.1	43	11 (9,12)	9 (8,11)	-16.5	38
Louisiana	5 (4,5)	5 (4,5)	-0.1	23	8 (4,13)	7 (4,11)	-13.6	5	0 (0,0)	0 (0,0)	-12.5	36	14 (10,15)	12 (9,14)	-14.7	45
Maine	5 (5,5)	5 (5,5)	0.5	38	7 (4,11)	6 (4,10)	-8.9	40	0 (0,0)	0 (0,0)	-11	40	8 (7,10)	7 (6,9)	-13.8	47
Maryland	6 (6,6)	6 (6,6)	-0.4	11	8 (5,12)	7 (4,11)	-9.2	38	0 (0,0)	0 (0,0)	-23.3	10	13 (9,14)	10 (8,11)	-25.2	21
Massachusetts	6 (6,6)	6 (6,6)	0.2	30	7 (4,11)	6 (4,10)	-12.3	10	0 (0,0)	0 (0,0)	-20.6	18	11 (9,12)	8 (7,9)	-24.5	23
Michigan	5 (5,5)	5 (5,5)	0.7	45	6 (4,9)	6 (4,9)	-8.1	43	0 (0,0)	0 (0,0)	-15.2	30	10 (9,10)	8 (7,9)	-19	32
Minnesota	4 (4,4)	4 (4,4)	-0.1	21	6 (4,10)	6 (3,9)	-11	21	0 (0,0)	0 (0,0)	-14.8	31	7 (6,9)	6 (5,9)	-18.3	35
Mississippi	4 (3,4)	4 (3,4)	-1.1	5	6 (3,9)	5 (3,8)	-12.8	9	0 (0,0)	0 (0,0)	-10.8	41	13 (9,15)	11 (9,13)	-15.7	41
Missouri	5 (5,6)	5 (5,6)	0.8	46	8 (5,12)	7 (4,11)	-9	39	0 (0,0)	0 (0,0)	-13.4	34	10 (9,11)	8 (7,9)	-18.4	34
Montana	6 (5,6)	6 (6,6)	0.7	41	9 (5,14)	8 (5,12)	-11.9	12	0 (0,0)	0 (0,0)	-8.6	47	10 (9,12)	9 (8,10)	-14.6	46
Nebraska	4 (4,4)	4 (4,4)	-0.5	9	5 (3,8)	5 (3,7)	-11.5	18	0 (0,0)	0 (0,0)	-13.5	33	9 (8,10)	7 (6,10)	-20.6	30

© 2020 GBD 2017 US Neurological Disorders Collaborators. *JAMA Neurology*.

Nevada	5 (5,5)	5 (5,5)	0.5	40	6 (4,10)	6 (4,9)	-3.9	50	0 (0,0)	0 (0,0)	-26.4	5	12 (9,13)	8 (7,9)	-32	5
New Hampshire	5 (5,5)	5 (5,5)	0.9	48	7 (4,10)	6 (4,9)	-11.9	14	0 (0,0)	0 (0,0)	-17.1	27	8 (7,10)	6 (5,9)	-22.2	27
New Jersey	5 (5,5)	5 (5,5)	0.3	32	7 (4,10)	6 (4,9)	-8.2	42	0 (0,0)	0 (0,0)	-30.4	3	12 (9,13)	8 (7,10)	-35	4
New Mexico	6 (6,6)	6 (6,6)	-0.2	18	10 (6,15)	9 (5,14)	-11.1	19	0 (0,0)	0 (0,0)	-17.2	26	13 (10,15)	11 (9,12)	-21.3	29
New York	6 (6,6)	6 (6,6)	0.7	42	8 (5,12)	7 (4,11)	-7.1	47	0 (0,0)	0 (0,0)	-33.9	2	10 (9,11)	6 (5,10)	-37	2
North Carolina	5 (4,5)	5 (4,5)	-0.2	16	7 (4,11)	6 (4,10)	-8.4	41	0 (0,0)	0 (0,0)	-21.7	16	11 (9,13)	8 (7,9)	-27.4	13
North Dakota	4 (4,4)	4 (4,4)	-0.4	10	7 (4,11)	6 (3,9)	-17.1	2	0 (0,0)	0 (0,0)	-4.5	49	9 (8,10)	8 (7,11)	-8	50
Ohio	5 (5,5)	5 (5,5)	0.3	33	7 (4,11)	6 (4,10)	-9.3	36	0 (0,0)	0 (0,0)	-11.1	39	9 (8,10)	8 (7,10)	-15.5	42
Oklahoma	5 (5,5)	5 (5,5)	0.4	35	7 (4,11)	6 (4,10)	-13.3	7	0 (0,0)	0 (0,0)	-3.3	50	10 (9,11)	9 (8,11)	-8.3	49
Oregon	3 (3,4)	3 (3,3)	-1.8	3	5 (3,8)	4 (3,7)	-10.1	29	0 (0,0)	0 (0,0)	-19.4	23	9 (8,10)	6 (5,9)	-27.1	14
Pennsylvania	5 (5,5)	5 (5,5)	0.2	29	8 (4,12)	7 (4,11)	-10.7	25	0 (0,0)	0 (0,0)	-20	21	10 (9,11)	8 (7,9)	-23.2	26
Rhode Island	4 (4,5)	4 (4,5)	-0.2	14	6 (4,10)	6 (3,9)	-9.5	34	0 (0,0)	0 (0,0)	-21.9	14	12 (9,13)	8 (7,10)	-29.2	10
South Carolina	5 (5,5)	5 (5,5)	0.1	26	7 (4,12)	7 (4,10)	-9.7	32	0 (0,0)	0 (0,0)	-19.2	24	13 (9,15)	10 (8,11)	-25.8	18
South Dakota	4 (4,4)	4 (4,4)	-0.2	15	5 (3,8)	5 (3,7)	-13.2	8	0 (0,0)	0 (0,0)	-8.6	48	11 (9,12)	9 (8,11)	-12.5	48
Tennessee	5 (5,5)	5 (5,5)	0.5	36	8 (4,12)	7 (4,10)	-11.5	16	0 (0,0)	0 (0,0)	-14.7	32	10 (9,11)	8 (7,10)	-21.5	28
Texas	5 (5,6)	6 (5,6)	0.9	47	8 (5,13)	7 (4,11)	-10.8	24	0 (0,0)	0 (0,0)	-20	20	12 (9,13)	8 (7,10)	-26.6	15
Utah	4 (4,4)	4 (4,4)	-1.3	4	6 (4,9)	5 (3,8)	-13.4	6	0 (0,0)	0 (0,0)	-12.6	35	10 (9,11)	8 (7,10)	-17.5	36
Vermont	4 (4,4)	4 (4,4)	-0.1	19	4 (3,6)	4 (3,5)	-14.7	3	0 (0,0)	0 (0,0)	-20.5	19	8 (7,10)	6 (5,9)	-24.8	22
Virginia	4 (4,5)	4 (4,5)	-0.1	22	6 (4,10)	6 (3,9)	-9.8	31	0 (0,0)	0 (0,0)	-22.5	13	12 (9,13)	9 (7,10)	-26.1	16
Washington	4 (3,4)	4 (3,4)	-0.8	7	5 (3,8)	4 (3,7)	-11.6	15	0 (0,0)	0 (0,0)	-20.8	17	8 (7,10)	6 (5,9)	-27.6	12
West Virginia	6 (5,6)	6 (5,6)	1	50	9 (5,13)	8 (4,12)	-10.5	27	0 (0,0)	0 (0,0)	-8.9	46	11 (10,13)	10 (8,11)	-15.1	43
Wisconsin	4 (4,4)	4 (4,4)	-0.3	12	6 (4,9)	5 (3,8)	-10.9	23	0 (0,0)	0 (0,0)	-15.9	29	8 (8,10)	7 (6,9)	-20.5	31

© 2020 GBD 2017 US Neurological Disorders Collaborators. *JAMA Neurology*.
